# Photocontrolled Isomerization Pathways of Tolylsulfonyl- and Pyrene-Linked Norbornadiene Systems Driven by Absorption Band Targeting

**DOI:** 10.1021/jacs.6c14776

**Published:** 2026-08-25

**Authors:** Dazhong Sun, Daniel Krappmann, Christoph Oleszak, Jonas Lienert, Chavdar Slavov, Andreas Hirsch, Josef Wachtveitl

**Affiliations:** † Institute of Physical and Theoretical Chemistry, Goethe University, Max-von-Laue-Straße 7, 60438 Frankfurt Am Main, Germany; ‡ Department of Chemistry and Pharmacy, Friedrich-Alexander Universität Erlangen Nürnberg, Nikolaus Fiebiger Straße 10, 91058 Erlangen, Germany; § Department of Chemistry, 7831 University of South Florida, Tampa, Florida 33620, United States

## Abstract

Norbornadiene (NBD) photoswitches are promising for molecular solar energy and information storage, as they isomerize to quadricyclane (QC) after absorbing irradiation energy. Here, we report pyrene-linked tolylsulfonyl-substituted NBD hybrids that enable wavelength-selective back-isomerization from QC to NBD. Using femtosecond-to-microsecond transient absorption spectroscopy in the deep-UV range, direct observation of QC formation could resolve the temporal windows of competing mechanisms, which confirm that the wavelength-dependent isomerization proceeds through two ultrafast charge-transfer (CT) pathways and longer-lived radical ion pair intermediates. Furthermore, chromophore coupling converts charge recombination from a loss channel into a productive process by enabling triplet energy transfer, leading to comparatively high quantum yields in both the switching directions. In a symmetric bis-NBD architecture, early CT productivity is reduced, and the mechanism shifts toward triplet-dominated isomerization, demonstrating how molecular symmetry redistributes pathway branching. Selective excitation of the QC unit or the pyrene chromophore biases singlet versus triplet channels, providing wavelength-dependent control over reaction efficiency. These results establish pathway engineering across singlet and triplet manifolds as a strategy for designing NBD systems for solar energy storage and optically addressable molecular information technologies.

## Graphical abstract

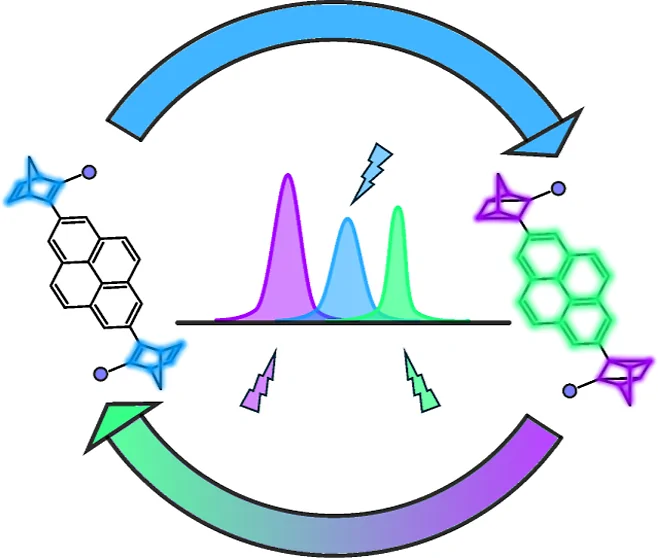

## Introduction

Norbornadiene (NBD) undergoes photoinduced isomerization to quadricyclane (QC), a higher-energy valence isomer, similar to classical photoswitches such as azobenzenes and spiropyrans. After molecular rearrangement, the QC has a different geometry and possesses higher energy in its gourd state. Owing to its high energy storage density, synthetic accessibility, and reversible photochemistry, the NBD/QC system has emerged as a promising platform for molecular solar thermal (MOST) energy storage,^1,2^ photocontrolled information processing,^3^ bioimaging,^4^ and semiconductor-related applications.^5^ Despite these promising properties, achieving efficient and selective photocontrol over the forward and backward isomerization processes remains a central challenge. In particular, a mechanistic understanding of how charge-transfer (CT) states, radical ion pairs, and triplet intermediates compete or cooperate during isomerization is essential for rationally controlling the QC ↔ NBD interconversion.

A major limitation of parent NBD is its absorption in the deep-ultraviolet (DUV) region, which restricts practical implementation under solar irradiation and in biological environments. To overcome this challenge, extensive synthetic efforts have focused on introducing electron-donating and electron-withdrawing substituents to shift the absorption spectrum into the red region.^2,6–8^ These push–pull architectures promote CT excited states and provide an alternative to previously reported approaches employing catalysts or sensitizers, in which electron transfer generates NBD radical ion pairs that evolve through triplet NBD/QC intermediates to mediate isomerization.

To date, molecular design strategies have largely emphasized either (i) CT-driven isomerization via push–pull substitution^9–11^ or (ii) radical ion pair formation followed by isomerization through the triplet state (Figure 1).^12–15^ Systems integrating both features within a single molecular architecture remain largely unexplored, and comprehensive time-resolved studies capable of resolving both CT-mediated and radical ion pair pathways are still lacking.

**Figure 1:**
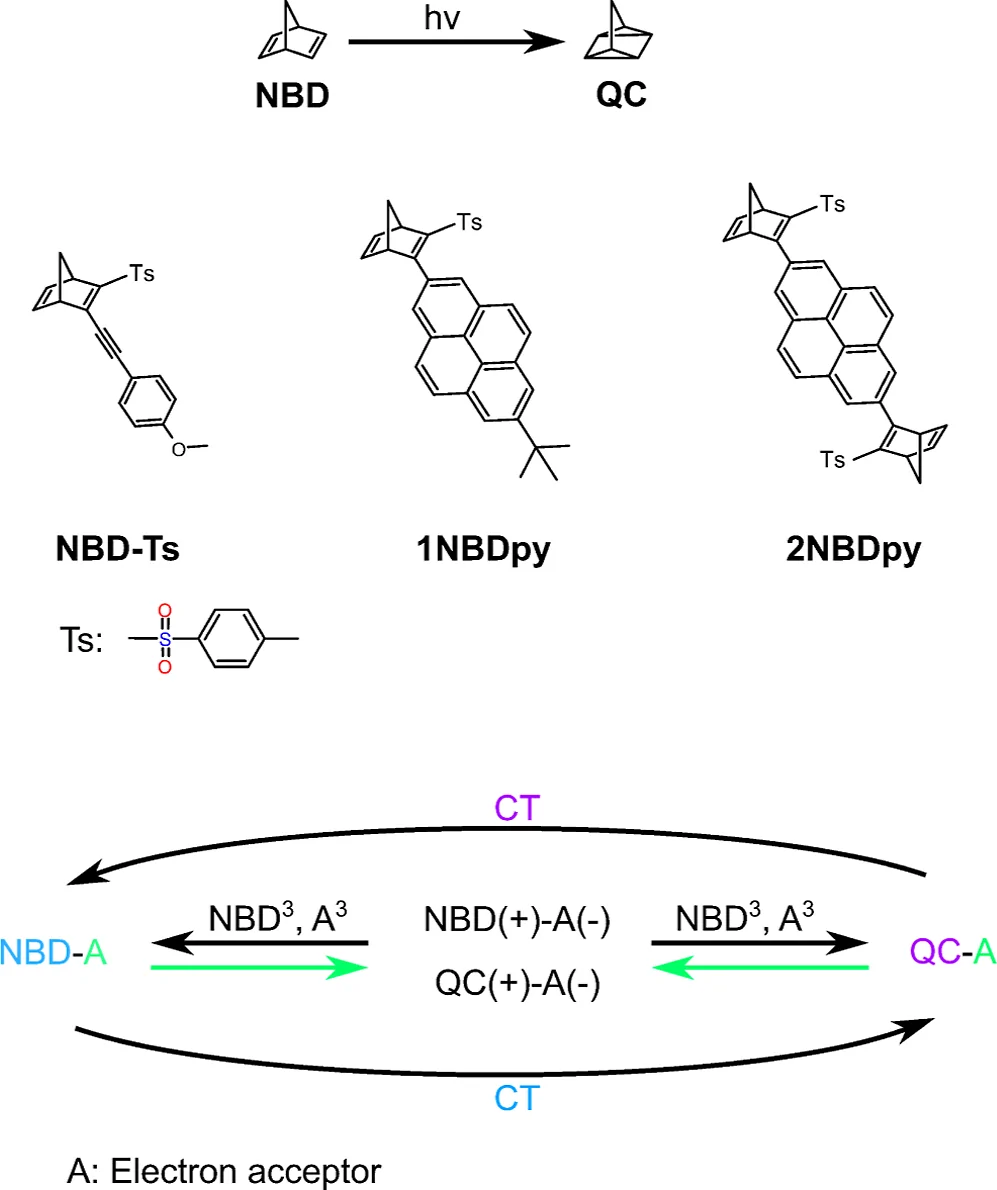
Investigated norbornadiene systems in this work (above) and typical norbornadiene isomerization pathways. The colored arrows and labels indicate the local excitation of different chromophores.

Because CT and radical ion pair mechanisms operate on distinct temporal regimes—from femtoseconds^9,16^ to nanoseconds^12,17^ —their coexistence could provide a powerful strategy for pathway-selective photocontrol.^4^ However, ultrafast spectroscopic evidence directly resolving mixed isomerization dynamics in substituted or chromophore-linked NBD systems remains scarce.^9,15,16^ Most mechanistic insights have instead been inferred indirectly from fluorescence quenching^17–19^ or calorimetric measurements.^20^ Furthermore, direct spectroscopic observation of QC formation has been hindered by its characteristic DUV absorption and the probing limitations of conventional supercontinuum sources. Consequently, previous transient absorption studies have predominantly monitored excited-state absorption without direct identification of product formation, leaving the time scales of QC generation and decay in substituted NBD systems unresolved.

Here, we report newly synthesized NBD derivatives covalently linked to a tolylsulfonyl group and pyrene (1NBDpy and 2NBDpy, Figure 1) designed to enable dual isomerization pathways through CT states and radical ion pairs. We compare their excited-state dynamics with those of the tolylsulfonyl-substituted NBD reference compound (NBD-Ts) to elucidate the role of chromophore coupling. In particular, the multi-NBD architecture of 2NBDpy provides an opportunity to evaluate how the covalent integration of multiple NBD units influences isomerization dynamics and potentially enhances energy storage density.

By directly probing in the DUV region, we resolve the characteristic absorption signatures of QC and fully map the excited-state evolution from femtoseconds to microseconds. This direct observation enables mechanistic assignment of CT-mediated and triplet-mediated isomerization pathways and rationalizes the comparatively high quantum yields observed in the chromophore-linked systems. Most notably, we demonstrate that the QC → NBD back-isomerization can be steered through wavelength-selective excitation, providing a strategy for pathway-selective photocontrol. These findings establish mechanistic design principles for NBD-based systems with enhanced photoresponsiveness and controllable energy storage behavior.

## Results and Discussion

The synthesis of all the NBD-Ts was reported in the work of Krappmann et al.,^10^ and the pyrene-linked NBDs were synthesized as described in the work from Oleszak et al. with the same pyrene precursor.^21^ The synthesis routine is included in the Supporting Information (Figure S1).

### Steady-State Characterization

#### NBD-Ts

NBD-Ts exhibit a broad absorption band centered at 340 nm (Figure 2A), which is assigned to the intramolecular CT transition introduced by the tolylsulfonyl substituent.^10^

**Figure 2:**
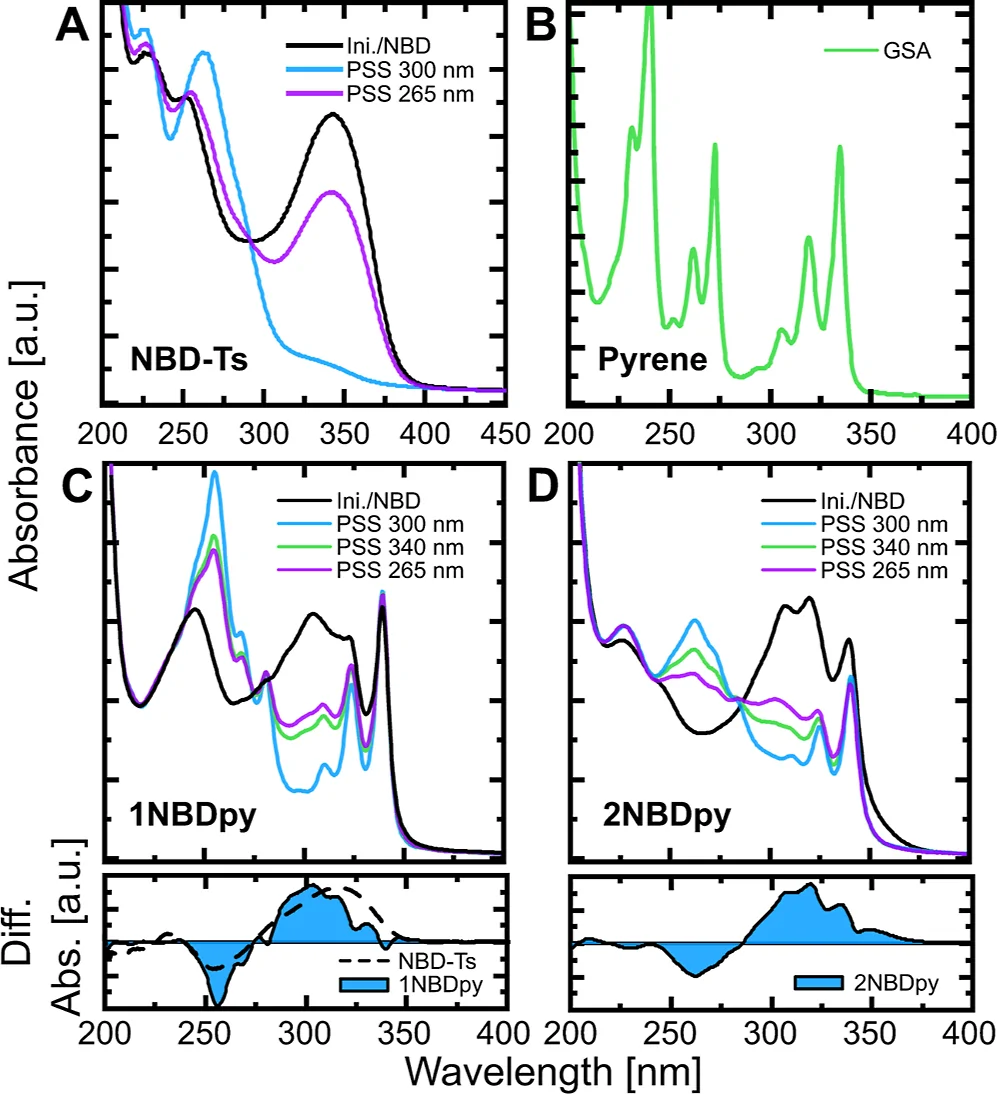
Steady and photostationary state (PSS) absorption spectra of all the investigated systems in acetonitrile. A: Photostationary States of NBD-Ts; B: Ground state absorption (GSA) spectrum of pyrene; C: Photostationary states of 1NBDpy (top) and its difference spectrum (solid blue) overlaid with difference spectrum of NBD-Ts (dash line); D: photostationary states of 2NBDpy (top) and difference spectrum between NBD form (bottom) and PSS 300 nm.

Upon irradiation at 340 nm (Figure 2A), a pronounced decrease of the CT band is observed, indicating efficient forward isomerization to QC-Ts. The local NBD absorption band at 251 nm concomitantly shifts to 263 nm, consistent with formation of the quadricyclane product.^9^ Three well-defined isosbestic points at 280, 250, and 200 nm confirm a clean two-state interconversion without detectable side products with a reported photostationary state conversion ratio of 94% with the same irradiation wavelength.^10^

Back-isomerization from QC-Ts to NBD-Ts could be induced by irradiation at 265 nm (Figure 2A), corresponding to the QC absorption maximum. The reappearance of the original spectral features and preservation of the same isosbestic points confirmed reversibility. However, complete back conversion could not be achieved due to spectral overlap between NBD and QC at the excitation wavelength.

The quantum yield for forward isomerization (NBD → QC) was determined to be 79% under 340 nm irradiation, while the back-isomerization (QC → NBD) proceeds with a quantum yield of 44% upon 265 nm excitation (See Supporting Information, Figures S8, S9), which is comparable to a reported similarly system.^22^

### NBD-Pyrene

The absorption spectra of 1NBDpy and 2NBDpy exhibit a superposition of pyrene-centered transitions and charge-transfer (CT) absorption of the substituted NBD unit (Figure 2C,D). The characteristic pyrene S_2_ vibronic progression at 335, 325, and 310 nm overlaps with the NBD CT band centered at ∼305 nm. In the deep-UV region, localized NBD absorption dominates, with 1NBDpy displaying a strong band at 245 nm, whereas 2NBDpy shows two weaker features. Upon excitation of the CT band at 300 nm, 1NBDpy undergoes efficient forward isomerization, evidenced by a decrease in the CT absorption and concomitant growth of a QC-associated band at 255 nm. The presence of multiple isosbestic points confirms a clean two-state conversion, and the difference spectra closely resemble those of NBD-Ts, indicating that the fundamental electronic reorganization is preserved upon chromophore linkage (Figure 2C). In contrast, 2NBDpy exhibits a similar decrease of the CT band and formation of QC features at 225 and 266 nm but lacks well-defined isosbestic points (Figure S13), reflecting more complex photochemical behavior. Note that the difference spectra all present a cutoff of the vibronic peaks from the pyrene absorption for both pyrene-linked NBDs (Figure 2C,D,bottom). This relative increase of the pyrene vibronic absorption is due to reduced electronic coupling between the NBD unit and pyrene upon the formation of QC. The maximum conversions are estimated to be 84% for 1NBDpy but only 67% for 2NBDpy (Supporting Information, Tables S1 and S2 in chloroform rather than acetonitrile), suggesting that the symmetric bis-NBD architecture reduces isomerization efficiency, although detailed mechanistic models require time-resolved analysis (vide infra). The quantum yield of forward isomerization for 1NBDpy is 45% (Supporting Information, Figure S10), exceeding values reported for related chromophore-linked systems.^14,19^

QC-to-NBD isomerization in both systems can be induced either by excitation of the pyrene chromophore at 340 nm or by direct excitation of the QC absorption at 265 nm. In both cases, recovery of the NBD CT band confirms reversibility, although a complete restoration of the initial spectrum is not achieved. Notably, excitation at 265 nm consistently yields a higher degree of back-conversion than excitation at 340 nm, as evidenced by the more pronounced recovery of the CT absorption around 305 nm (Figure 2C,D). This wavelength dependence indicates that distinct excited-state pathways are accessed, depending on the excitation channel. The effect is more pronounced in 2NBDpy (Figure 2C,D, difference between the green and purple lines), suggesting that multi-NBD coupling enhances pathway selectivity. For 1NBDpy, the quantum yield of back-isomerization is 17% upon 340 nm excitation and 22% upon 265 nm excitation.

### Time-Resolved Measurements

Femtosecond-to-microsecond transient absorption measurements were performed to capture all possible excited-state evolution of NBDs, including charge-transfer (CT) states, radical ion pairs, and triplet intermediates. Broadband probing was conducted in both the DUV (250–390 nm) and UV–vis (350–650 nm) regions to enable direct monitoring of excited-state absorption (ESA), ground-state bleach (GSB), and product formation (PA). The excited states of each species were populated by excitation pulses chosen as in the steady-state measurements: 340, 308, and 267 nm (see Figure 2). The transient data sets were analyzed using global lifetime analysis implemented in the OPTIMUS software package.^23^ The measurement data and analysis are presented as combined sets and scaled according to the edge of the difference in absorption data and lifetime amplitude.

### NBD-Ts to QC-Ts Isomerization

Immediately following photoexcitation, a broad transient absorption spanning 370–650 nm appears, which is assigned to a CT excited state characteristic of push–pull substituted NBD systems (Figure 3, top).^9^ Global analysis yields a lifetime of approximately 110 fs for this component (Figure 3, bottom). Concurrent with the decay of the CT signal, positive absorption features emerge in the 260–280 nm region corresponding to the characteristic absorption of quadricyclane (QC). The prompt buildup of this product band demonstrates that the CT state directly populates the isomerized ground-state surface on an ultrafast time scale.

**Figure 3:**
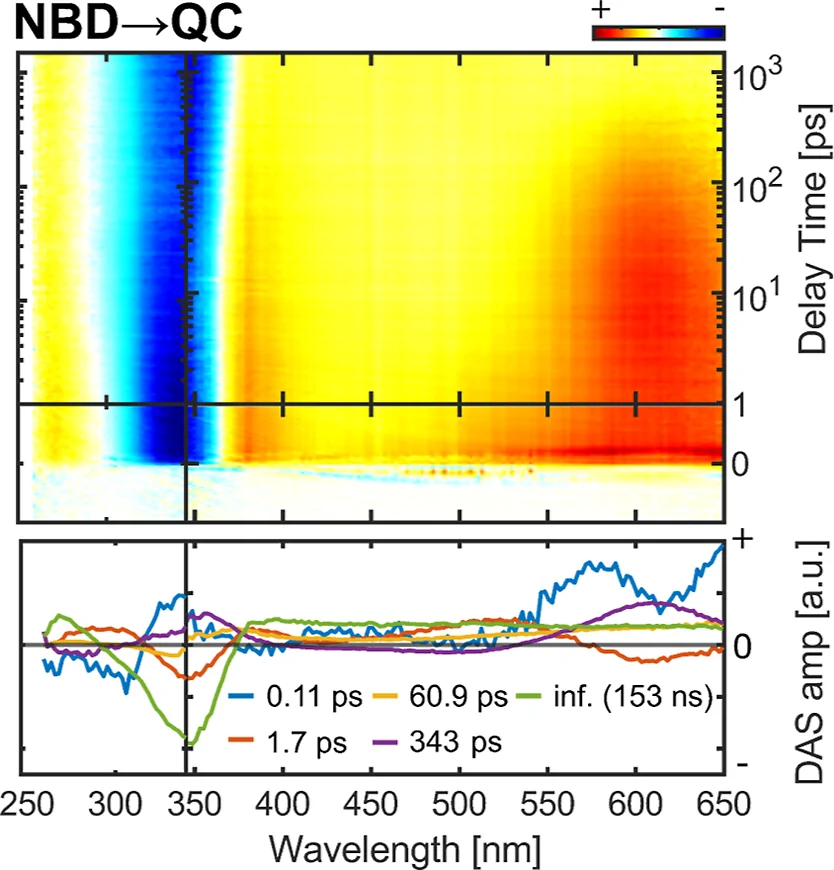
Transient absorption data (above) and the corresponding decay-associated spectra (below) of NBD-Ts in acetonitrile excited by 340 nm pulses. In the transient data, the positive signals present the excited state absorption or product formation, and the negative signals present the ground state bleach. The measurements from the UV–vis and DUV regions are separated by a vertical line (top) at 345 nm, as well as the corresponding decay-associated amplitudes (bottom). The scaling is based on the last (infinitive) amplitude component from the decay-associated spectra.

The decay of the CT state is followed by a spectral evolution on a 1.7 ps time scale (Figure 3, bottom) characterized by continued growth and blue-shifting of the QC absorption band (toward 270–280 nm) together with the recovery of the NBD ground-state bleach in the 360–400 nm region. This component is assigned to vibrational relaxation of a hot ground-state species formed after passage through a conical intersection. The combined observation of ground-state bleach recovery and stabilization of the QC absorption supports an internal conversion mechanism consistent with ultrafast valence isomerization.

In parallel with the productive ultrafast channel, a distinct charge-separated pathway was observed. A transient absorption feature centered at 605 nm develops with delayed kinetics relative to the CT decay. This band is assigned to the NBD radical cation based on its spectral position according to the literature reports.^24^ This charge-separated intermediate requires three kinetic components for adequate description: the 1.7 and 60 ps components, attributed to structural and solvent relaxation of the ion pair, present clear cooling signatures which are the narrowing of the NBD cation band at 605 nm and narrowing of the 385 nm band toward the ground-state bleach. The longer 343 ps decay component represents the charge recombination process. Notably, the decay of this intermediate is accompanied by little concurrent growth of QC absorption in the deep-UV region. This observation indicates that back electron transfer dominates and that the radical ion pair pathway does not significantly contribute to productive QC formation on this time scale.

At the end of the detection time, the positive background evolves to a broad absorption in the nanosecond range (375–550 nm) and persists with a lifetime of approximately 311 ns (see Supporting Information, Figure S17), which is assigned to the triplet state of NBD.^9^ In contrast to the prior report^9^ on related push–pull systems, partial recovery of the ground-state bleach is observed during triplet decay. This behavior suggests that the triplet state relaxes predominantly back to the NBD ground state and may also contribute to minor QC formation rather than leading primarily to irreversible photodegradation.

Overall, the ultrafast dynamics of NBD-Ts reveal two competing pathways originating from the initially formed CT state: (i) a dominant ultrafast productive channel proceeding through a conical intersection to yield QC within the first few picoseconds and (ii) a slower charge-separation channel forming a radical ion pair that evolves to the triplet manifold and predominantly relaxes back to NBD. The relative amplitudes of these pathways determine the overall efficiency of the isomerization efficiency.

### QC-Ts to NBD-Ts Isomerization

The back-isomerization dynamics were investigated by selectively exciting the preformed QC-Ts at 267 nm. In contrast to the forward process, the backward reaction is dominated by rapid relaxation through a CT-like excited state with minimal contribution from the radical ion pair pathway.

Upon excitation of QC-Ts, two principal transient features are observed: (i) a pronounced ground-state bleach in the 250–280 nm region corresponding to depletion of QC and (ii) a broad positive absorption extending from approximately 300 to 450 nm consistent with regeneration of NBD.

Immediately after excitation, a broad ESA spanning the full detection window appears and decays with a lifetime of 100 fs (Figure 4). This component is assigned to a CT-type excited state formed from the QC. Notably, formation of the NBD absorption band near 350 nm occurs essentially concomitantly with a decay of this CT state, indicating direct access to the NBD ground-state surface on an ultrafast time scale. Following CT decay, spectral evolution is observed over two characteristic time scales (4.6 and 44 ps). During this period, the initially broad absorption narrows and converges toward the steady-state CT absorption profile of NBD (Figure 2A). This behavior is assigned to vibrational relaxation and structural reorganization within the hot NBD ground state formed after internal conversion.

**Figure 4:**
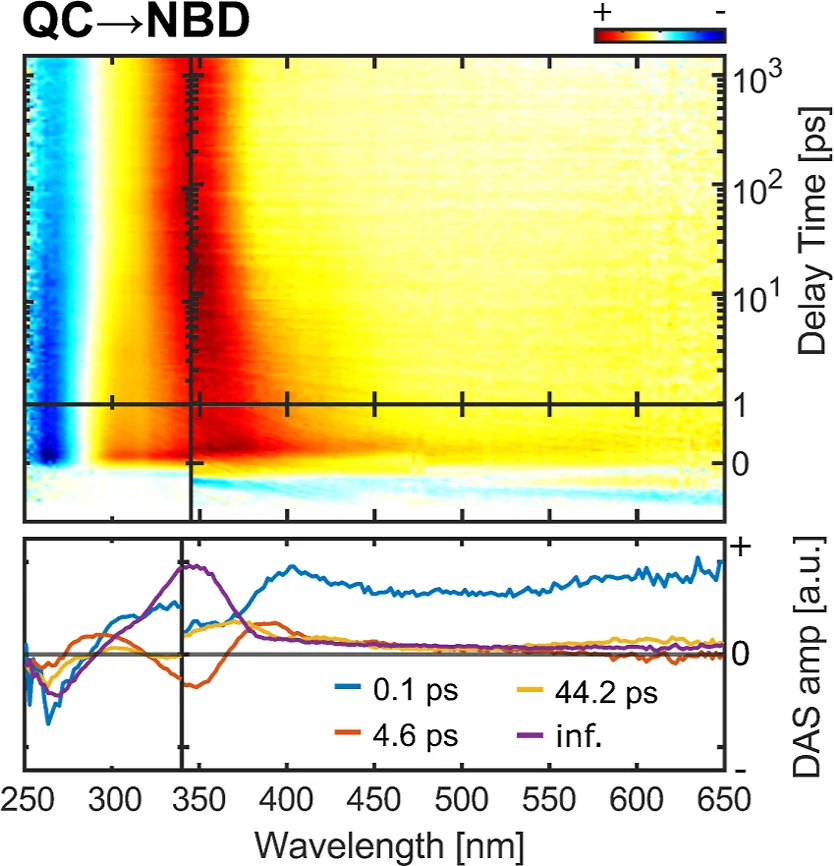
Transient absorption data (above) and the corresponding decay-associated spectra (below) of QC-Ts in acetonitrile excited by 267 nm pulses. The measurements from the UV–vis and DUV regions are separated by a vertical line (top) at 340 nm, as well as the corresponding decay-associated amplitudes (bottom).

In contrast to the forward reaction, only a very weak transient signal is detected near 605 nm, indicating minimal formation of the NBD radical cation during back-isomerization. Consistent with this observation, the infinite-time decay-associated spectrum exhibits only a faint residual absorption extending toward 550 nm, which can be attributed to a minor population of the NBD triplet state formed via the weakly populated radical ion pair channel.

Thus, the QC → NBD back-isomerization proceeds predominantly through a direct CT-mediated pathway involving ultrafast internal conversion, with only a negligible contribution from charge separation and triplet formation.

### Excited State Dynamics of Pyrene

To provide a reference for interpreting the chromophore-linked systems, the excited-state dynamics of pyrene were measured under identical conditions. While the photophysics of pyrene has been extensively characterized,^25–27^ DUV probing of its evolution into the nanosecond-microsecond regime remains limited. The present measurements therefore serve as a direct reference for comparison with the NBD-pyrene dyads. The measurement of the picosecond excited-state dynamics of pyrene can be found in the Supporting Information (Figure S18).

After excitation, the S_1_ state (ESA absorption at 360 and 470 nm) persists with a lifetime of 15.4 ns before undergoing intersystem crossing to the triplet manifold with absorption peaks at 380 and 410 nm (Figure 5).^28^ The resulting triplet absorption displayed a lifetime of 176 ns under the present experimental conditions. This lifetime is shorter than values reported for rigorously degassed samples,^29^ indicating partial oxygen quenching of the triplet state.

**Figure 5:**
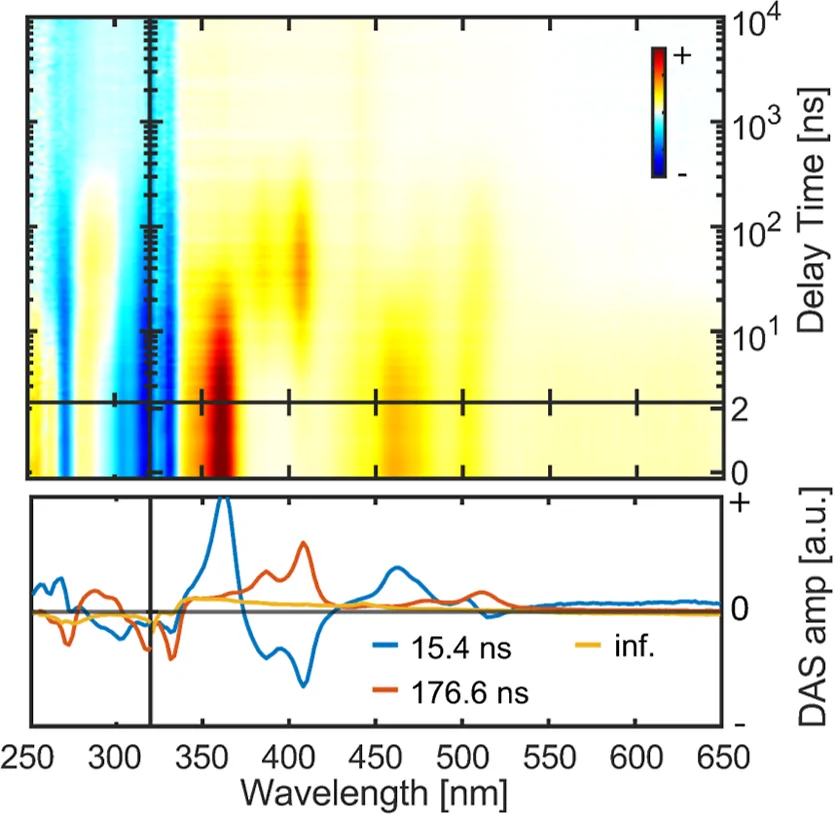
Transient absorption data (top) and the corresponding decay-associated spectra (bottom) of pyrene in acetonitrile excited by 340 nm pulses. The measurements from the UV–vis and DUV regions are separated by a vertical line (top) at 320 nm, as well as the corresponding decay-associated amplitudes (bottom).

### Isomerization of 1NBDpy (NBD → QC)

In contrast to NBD-Ts, the pyrene-linked system 1NBDpy exhibits coupled charge-transfer and interchromophoric charge-separation dynamics, leading to the population of both NBD- and pyrene-centered triplet states.

Immediately following excitation of the NBD CT band, a broad excited-state absorption spanning 340–650 nm is observed, closely resembling that of NBD-Ts (Figure 6B). Superimposed on this feature is an overlap from the pyrene S_2_ absorption around 575 nm. Global lifetime analysis yields an ultrafast component of 300 fs (Figure 6D). Because this lifetime approaches the instrument response function, the initially excited CT state and locally excited pyrene states cannot be kinetically separated and are treated as a single early component (Figure 6D).

**Figure 6:**
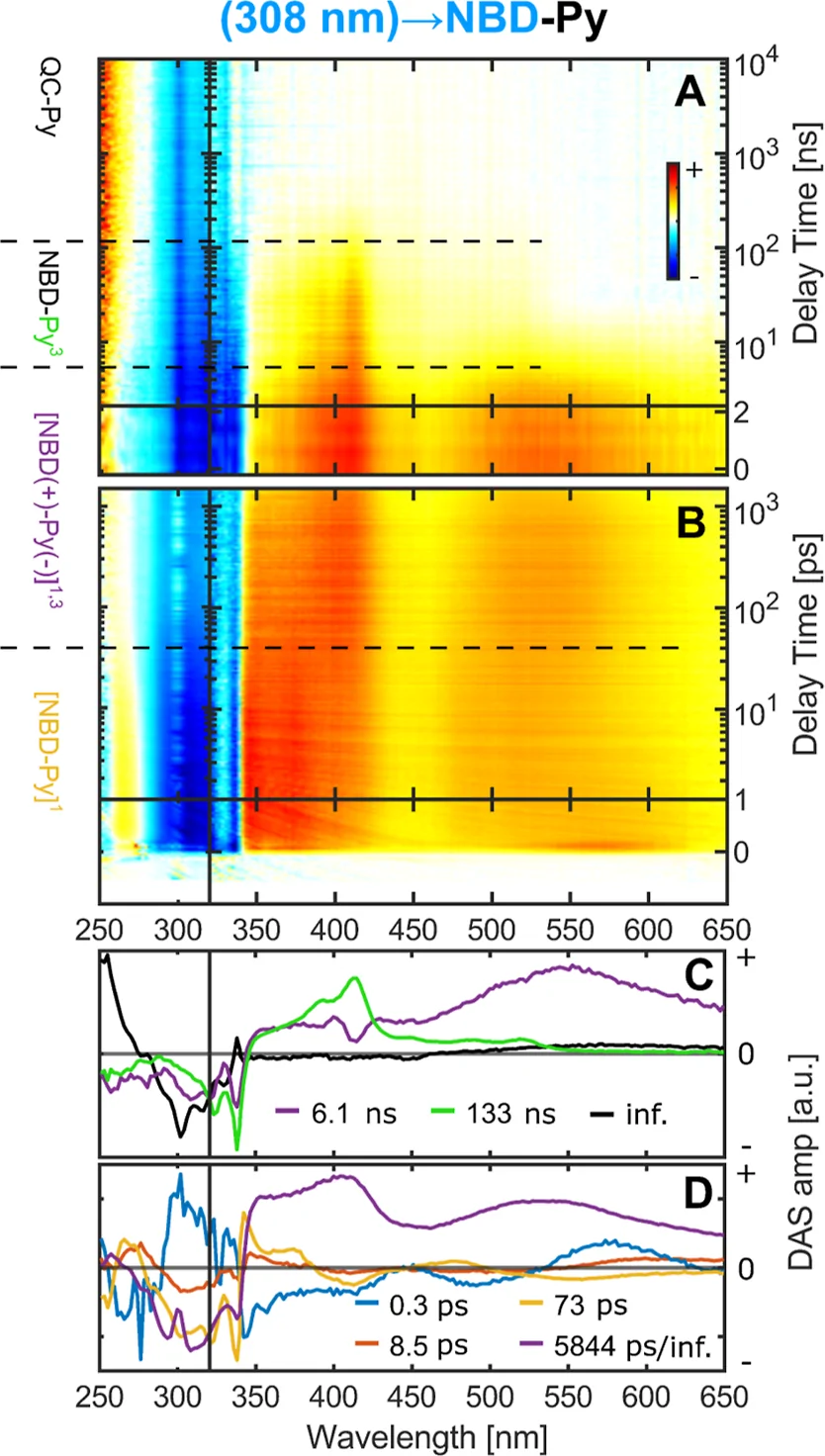
Transient absorption data of 1NBDpy in acetonitrile from nano- to microsecond (A) and femto- to nanosecond range (B), excited by 308 nm pulses, and the corresponding decay-associated spectra (C for A and D for B) of 1NBDpy. The measurements from the UV–vis and DUV regions are separated by the vertical line (top) at 345 nm, as well as the corresponding decay-associated amplitudes (bottom). The color scaling of the nanosecond measurements is aimed at clearly visualizing the absorption features rather than exact match of the ending of the picosecond measurements. The dominate excited species are noted near the transient data and separated by dash lines.

Subsequent spectral evolution revealed two concurrent processes. First, a blue-shifting positive signal emerges in the deep-UV region (260–280 nm), converging toward the characteristic QC absorption band, consistent with ultrafast isomerization via a conical intersection as in the case of NBD-Ts. Second, two broad absorption clusters develop in the visible region: one spanning 455–625 nm and another between 345 and 425 nm. The former feature evolves into a broad band centered near 525 nm and is assigned to the hot charge-separated radical–ion pair involving NBD and pyrene.^30^ The latter cluster is attributed to 2 overlapping contributions: (i) the hot NBD radical cation absorption tilted toward the ground-state bleach, analogous to the radical ion pair observed in NBD-Ts; and (ii) weak and broad absorption from the pyrene anion at 375 nm. Global fitting requires two components to describe the evolution. The faster (8.5 ps) component is primarily associated with vibrational relaxation of the hot ground-state species formed during QC/NBD ground state generation, as in the case of NBD-Ts. The slower (73 ps) component corresponds to electron recombination within the singlet radical ion pair and transition to the metastable singlet/triplet radical ion pair (Figure 6D).

At longer time scales (Figure 6A), this long-lived radical ion pair species persists with characteristic absorption peaks at approximately 410 and 525 nm and a lifetime of 5–8 ns. This metastable singlet/triplet ion pair decays predominantly into the triplet state manifold and partially undergoes direct singlet recombination to the ground state, as indicated by the ground-state bleach recovery during its decay (Figure 6A).

The ensuing triplet-state dynamics are dominated by pyrene-centered triplet absorption features at 390, 410, 480, and 515 nm, accompanied by weaker NBD triplet contributions spanning 350–550 nm (Figure 6A,C). The decay traces can be adequately described by a single lifetime of 133 ns (Figure 6C), suggesting strongly coupled triplet states. Notably, this lifetime is shorter than that of isolated pyrene under identical conditions, indicating possible intramolecular triplet energy transfer.

Importantly, growth of the QC absorption band (Figure 6A, around 260 nm) is observed during decay of the triplet states, demonstrating that the triplet state mediates additional NBD → QC isomerization. Note that the infinitive lifetime amplitude (Figure 6C, black line) matches well the difference absorption spectrum in steady-state measurement (Figure 2C), indicating a complete isomerization reaction. This secondary pathway provides a productive channel even when singlet recombination of the radical ion pair is inefficient, thereby contributing to the comparatively high quantum yield observed for 1NBDpy.

### Back-Isomerization of 1NBDpy (QC → NBD)

The QC → NBD back-isomerization of 1NBDpy follows a mechanistic sequence similar to the forward reaction, involving evolution from a CT state to a radical ion pair and subsequently to the triplet manifold. A key feature of the back reaction is the rapid formation of a QC-centered radical ion pair consisting of a QC cation and pyrene anion, as evidenced by the well-structured, characteristic pyrene radical anion absorption bands at 275, 375, and 485 nm (Figure 7B,F), which could be expressed by lifetimes of 43 to 62 ps (Figure 7D,H).^26,31^ Note that the clearer observation of the pyrene radical anion during QC → NBD back-isomerization likely reflects the reduced π-conjugation between QC and pyrene relative to NBD, leading to more localized charge separation. This intermediate subsequently evolves into the NBD-pyrene triplet ion pair previously observed in the forward isomerization (Figure 7, purple line in the DAS amplitudes). A major difference between the two excitation schemes is the wavelength-dependent population of the CT state. Excitation at 340 nm, which primarily addresses the pyrene chromophore, favors formation of the radical ion pair, as reflected by stronger GSB recovery below 260 nm (Figure 7A,B) and more intense NBD triplet ion pair absorption on the nanosecond time scale. In contrast, excitation at 267 nm directly accesses the QC absorption band and preferentially populates the CT state, resulting in the immediate appearance of the NBD product absorption on the femtosecond time scale (Figure 7F) and a significantly stronger amplitude of the subsequent hot state (Figure 7H, red line). These differences explain the higher back-isomerization conversion ratio observed under 267 nm excitation in steady-state measurements. Notably, decay of the pyrene triplet state is accompanied by additional growth of the NBD absorption around 310 nm, indicating that the QC → NBD isomerization can also be mediated by the triplet state of pyrene and norbornadiene. This bidirectional role of the triplet pathway also provides a mechanistic explanation for the incomplete photostationary recovery observed under steady-state conditions.

**Figure 7:**
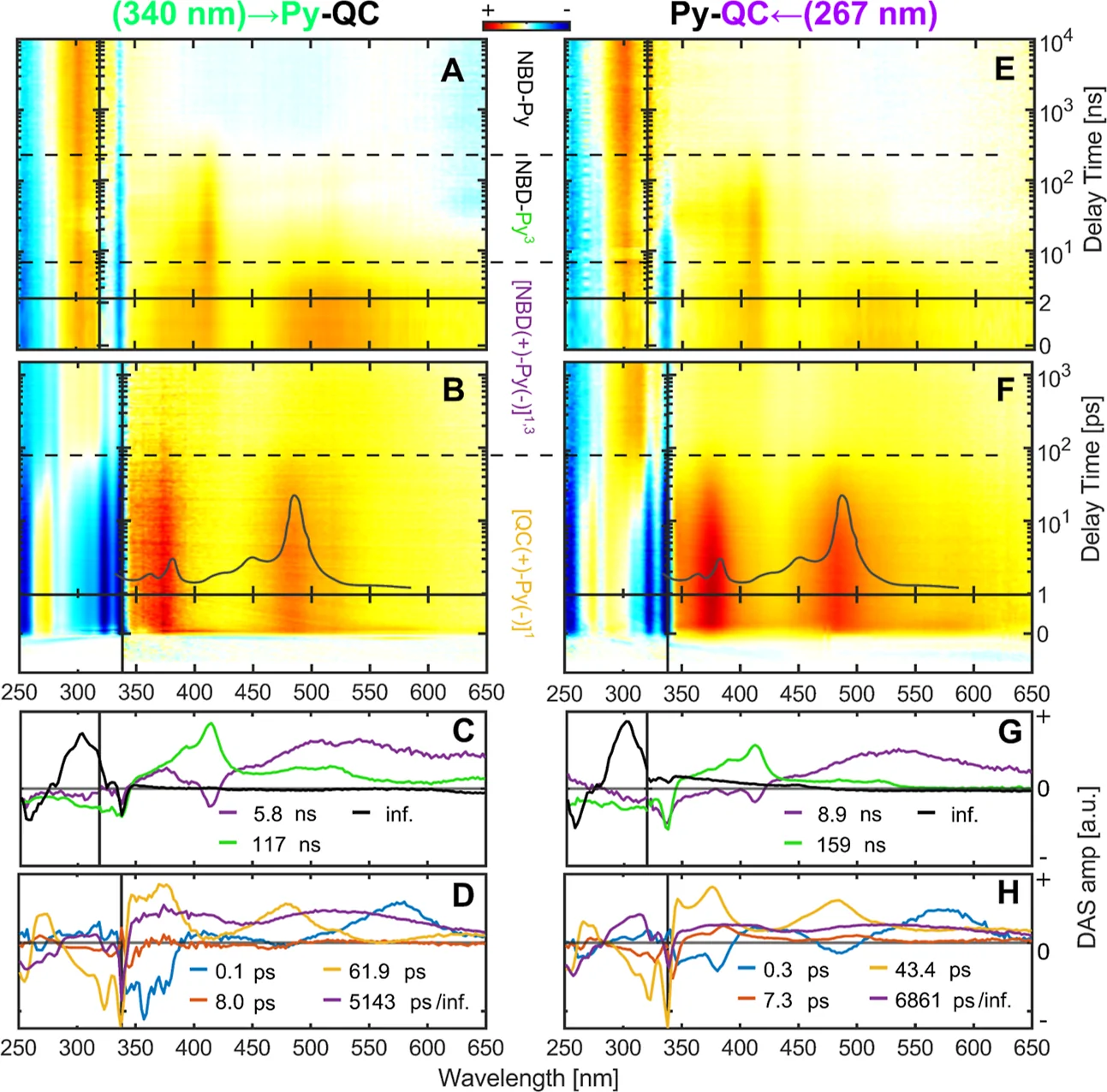
Transient absorption data of 1QCpy in acetonitrile excited by 340 and 267 nm pulses from nano- to microsecond­(A, E) and femto- to nanosecond range­(B, F). Corresponding decay-associated spectra (C for A, D for B, G for E, and H for F). The measurements from DUV and UV–vis region are separated by vertical lines at 320 nm for the nanosecond range and at 338 nm for the picosecond range, as well as the corresponding decay-associated spectra. The absorption spectra of pyrene anion^26,31^ are also presented in B and F for comparison (solid dark lines).

### Isomerization 2NBDpy (NBD → QC)

The excited-state dynamics of 2NBDpy were investigated under identical experimental conditions to those of 1NBDpy to enable direct comparison. The symmetric architecture containing two covalently linked NBD units significantly alters the early time branching behavior and redistributes the relative contributions of singlet and triplet pathways.

Following excitation of the CT band, 2NBDpy exhibits initial dynamics analogous to those of 1NBDpy, including formation of a charge-transfer state that evolves toward a radical ion pair intermediate. However, clear differences emerge within the first few picoseconds.

Although CT-like excited-state absorption spanning 350–650 nm is observed, the buildup of the QC absorption band in the deep-UV region is markedly weaker during the early picosecond time window (Figure 8B, below 260 nm). This behavior contrasts with 1NBDpy, where QC formation is clearly resolved on the subpicosecond time scale (Figure 6B). Considering the great change in the difference spectra in the steady-state measurements (Figure 2C), the absence of a correspondingly strong early QC signal therefore suggests reduced productivity of the CT-mediated isomerization channel. The residual tilted DUV signal resembling the hot ground-state feature observed in 1NBDpy remains detectable but is partially compensated by ground-state bleach recovery, indicating only minor ultrafast isomerization.

**Figure 8:**
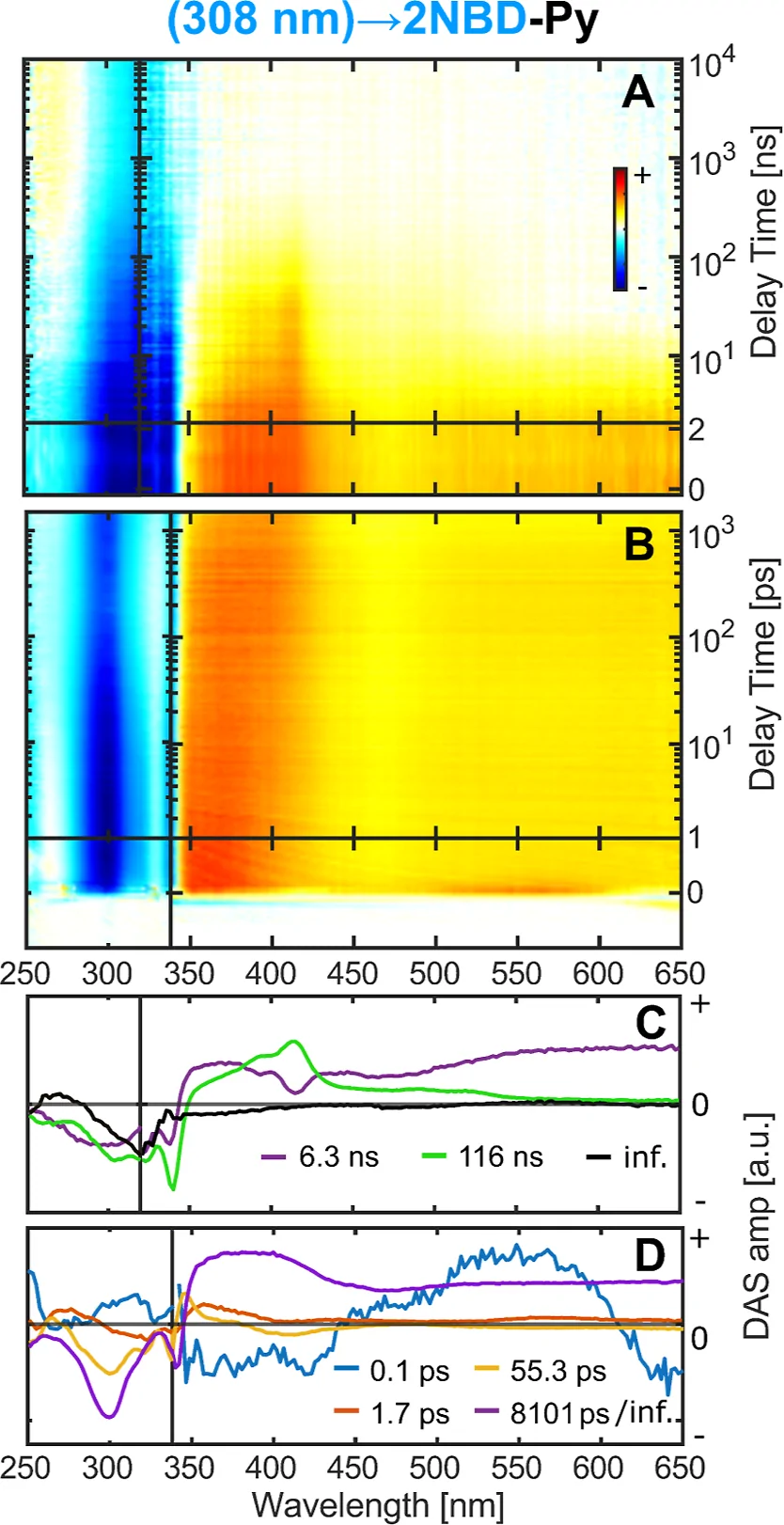
Transient absorption data of 2NBDpy in acetonitrile excited by 308 nm pulses from nano- to microsecond (A) and femto- to nanosecond range (B) and the corresponding decay-associated spectra (C for A and D for B). Note that there is a slight artifact at the early nanosecond range, which results in discontinuity of the ground state bleach at 320 nm between the picosecond and nanosecond measurements.

Simultaneously, the radical ion pair absorption in the visible region appears broader and less structured (Figure 8B) compared to 1NBDpy (Figure 6B), extending continuously from 350 to 650 nm. This spectral broadening is consistent with increased charge delocalization across the extended π-system of the symmetric bis-NBD architecture.

At longer time scales, QC formation becomes clearly correlated with the decay of the pyrene triplet state rather than with the early CT relaxation (Figure 8A). Growth of the QC absorption band is observed predominantly following population and decay of the triplet manifold, indicating that the triplet pathway constitutes the dominant productive channel in 2NBDpy. Overall, the NBD-to-QC isomerization of 2NBDpy is shifted toward a triplet-dominated regime with suppressed direct CT productivity in the early picosecond range.

### Back-Isomerization of 2NBDpy (QC → NBD)

The QC → NBD back-isomerization dynamics of 2NBDpy display even more pronounced wavelength dependence than observed for 1NBDpy.

As in the monosubstituted system, excitation at 267 nm (direct QC excitation) more efficiently populates the CT-like state compared with 340 nm excitation of the pyrene chromophore. However, in 2NBDpy, the CT-mediated formation of NBD is further suppressed, and the difference between excitation wavelengths becomes more substantial.

Upon 267 nm excitation, in the early nanosecond range, the formation of NBD absorption is detectable, whereas under 340 nm excitation, little to no NBD product signal is observed within the first nanoseconds, as positive NBD absorption around 300 nm becomes clearly visible at 2 ns for 267 nm excitation (Figure 9F) but remains negligible for 340 nm excitation (Figure 9B). This enhanced wavelength selectivity is consistent with the steady-state photoconversion data (Figure 2D). Decay-associated spectra also reveal a significant hot-ground-state contribution following CT relaxation (Figure 9H, red line) for 267 nm excitation, indicating appreciable population of the CT state even in the symmetric architecture.

**Figure 9:**
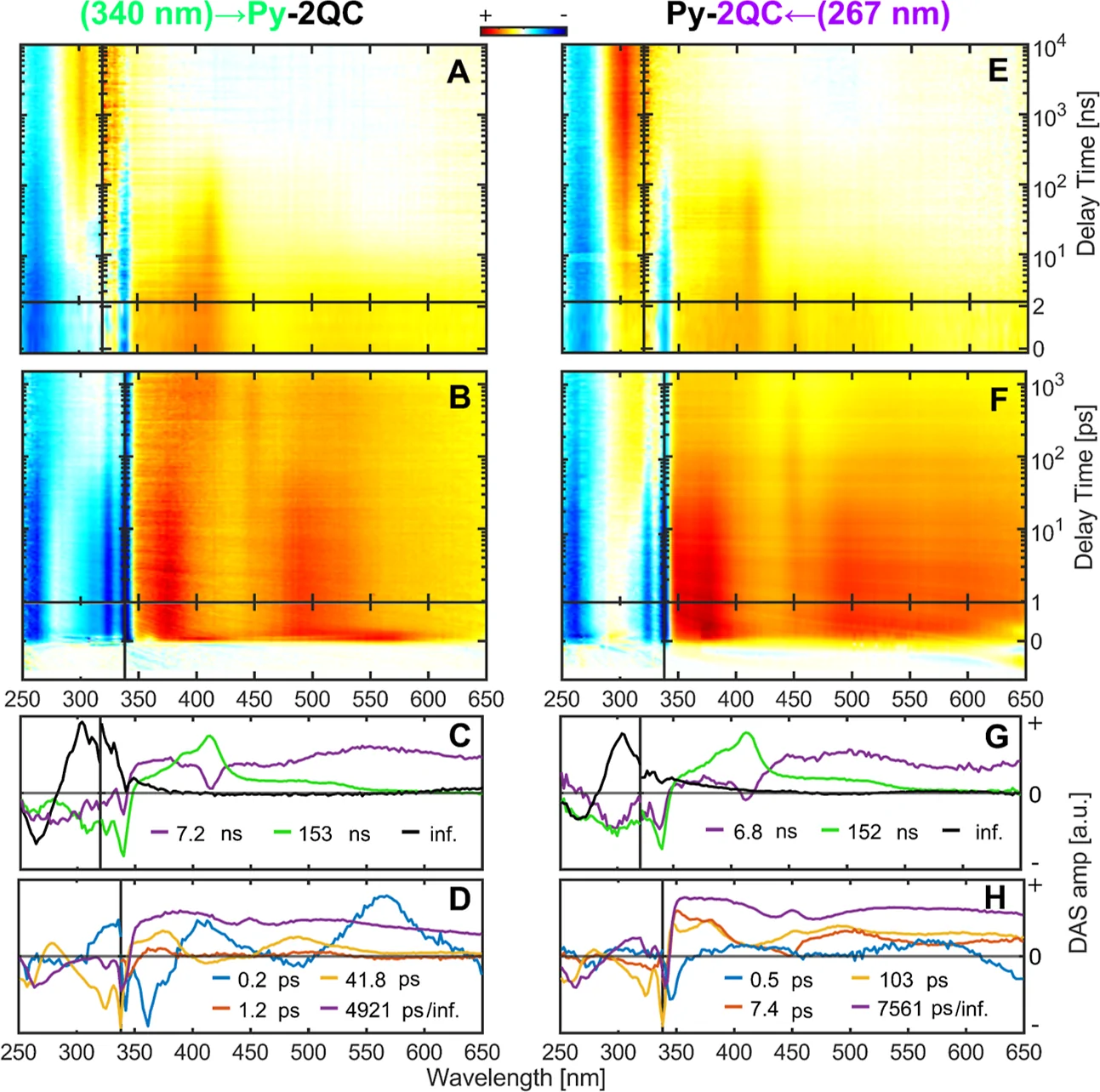
Transient absorption data of 2QCpy in acetonitrile excited by 340 and 267 nm pulses from nano- to microsecond (A, E) and femto- to nanosecond range (B, F) and the corresponding decay-associated spectra (C for A, D for B, G for E, and H for F). The measurement A has noticeable noise around 320 nm, so the scaling of the lifetime components is based on the second last component rather than the infinitive component.

Nevertheless, most of the NBD formation occurs on the nanosecond time scale and correlates with the decay of the triplet manifold (Figure 9A,E). As in the forward reaction, the triplet pathway therefore represents the dominant productive channel for back-isomerization in 2NBDpy.

In addition to the expected pyrene radical anion signatures, transient features assignable to pyrene radical cation absorption near 450 nm^26^ are observed in both excitation schemes (Figure 9B,F). The simultaneous presence of cationic and anionic pyrene signatures suggests the possibility of intermolecular charge separation or excited-state interactions beyond a simple intramolecular QC-pyrene radical ion pair. Given the extended π-system and the excimer formation observed in steady-state fluorescence measurements (see supporting information Figure S17), transient intermolecular interactions cannot be excluded. Such interactions may contribute to broadened radical ion pair features and could potentially influence triplet decay dynamics. However, further studies would be required for definitive assignment.

## Discussion and Conclusion

The combined steady-state and ultrafast measurements reveal a systematic redistribution of isomerization pathways across NBD-Ts, 1NBDpy, and 2NBDpy, demonstrating how chromophore coupling and molecular symmetry govern branching between singlet and triplet channels.

In NBD-Ts, isomerization (NBD → QC) proceeds predominantly through an ultrafast charge-transfer (CT)-mediated pathway that accesses the product ground state within a few picoseconds via a conical intersection. A competing radical ion pair branch evolves toward the triplet manifold and contributes only minimally to productive QC formation. In contrast, back-isomerization (QC → NBD) is largely confined to the CT-mediated channel, revealing intrinsic mechanistic asymmetry between the two directions (Figure 10).

**Figure 10:**
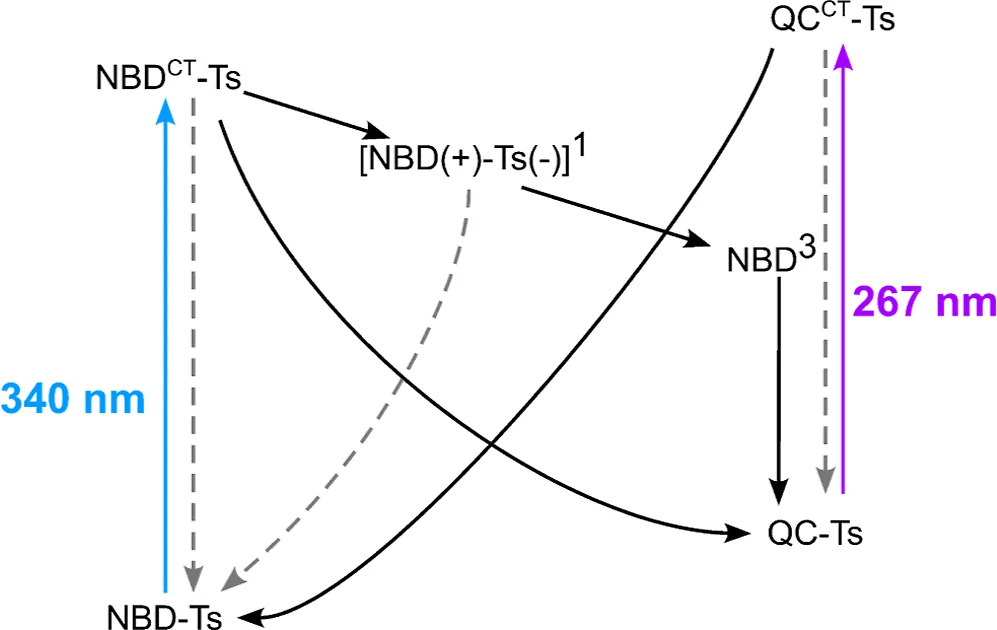
Productive reaction pathways of NBD/QC-Ts for isomerization in both directions. The colored arrows represent the excitation wavelengths (340 and 267 nm). The black solid arrows indicate the productive pathways, while the gray dashed arrows depict the nonproductive pathways. The apparent rate constants are given in the Supporting Information (Figure S25).

Covalent attachment of pyrene in 1NBDpy fundamentally alters this balance. While ultrafast CT-mediated isomerization remains operative, the radical ion pair becomes longer-lived and efficiently populates coupled NBD and pyrene triplet states. Decay of the triplet manifold is temporally correlated with additional product growth, establishing triplet relaxation and energy transfer as a productive secondary pathway (Figure 11). Thus, in 1NBDpy, charge separation is no longer merely a loss channel but is partially redirected toward isomerization through triplet mediation. This explains the relatively high quantum yield compared to previously reported chromophore-linked systems^17,18^ and is consistent with a similar system reported by Ghasemi et al., which also contains pyrene as a chromophore.^4^ Furthermore, our work provides a convincing mechanism to explain the QC-to-NBD isomerization observed in Ghasemi’s work and supports the use of pyrene in NBD systems. Furthermore, such a reaction mechanism involving 2 reaction pathways could also explain the isomerization conversion ratio for recent chromophore-linked systems,^14,32^ as the population of the CT and radical-ion-pair states could be altered by the length of the covalent alkyl chain and the polarity of solvents.

**Figure 11:**
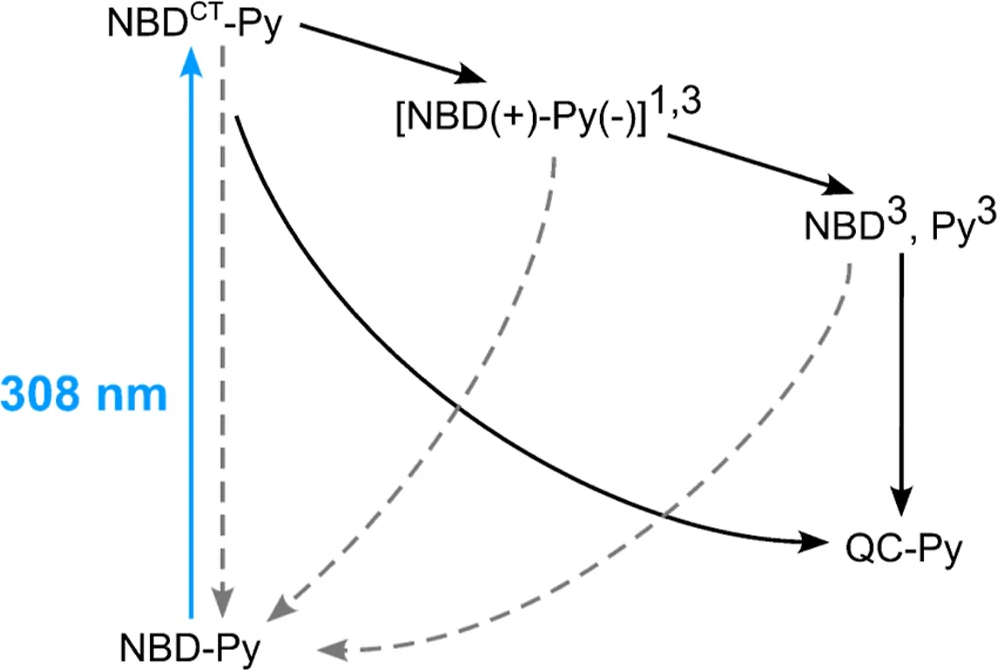
Reaction pathways of pyrene-linked NBD for photoinduced NBD-to-QC isomerization. The blue arrow represents the excitation. The solid black arrows indicate the major productive pathways, while the gray dashed arrows depict the nonproductive pathways. The apparent rate constants are given in the Supporting Information (Figure S26).

In the symmetric bis-NBD system 2NBDpy, early CT productivity is significantly reduced. Despite efficient CT-state formation, ultrafast QC generation is suppressed, and product formation correlates primarily with triplet decay. The broadened radical ion pair signatures and delayed product growth indicate enhanced charge delocalization within the extended π-system, which stabilizes charge-separated configurations and shifts the dominant pathway toward triplet-mediated switching.

A key finding is the pronounced wavelength dependence of back-isomerization in the chromophore-linked systems. Direct excitation of the QC absorption band preferentially accesses the CT-mediated pathway, yielding faster NBD formation (Figure 12), whereas pyrene excitation enhances charge separation and delays the productive conversion. This excitation-dependent branching becomes more pronounced in 2NBDpy, highlighting molecular symmetry as a lever for tuning the pathway selectivity.

**Figure 12:**
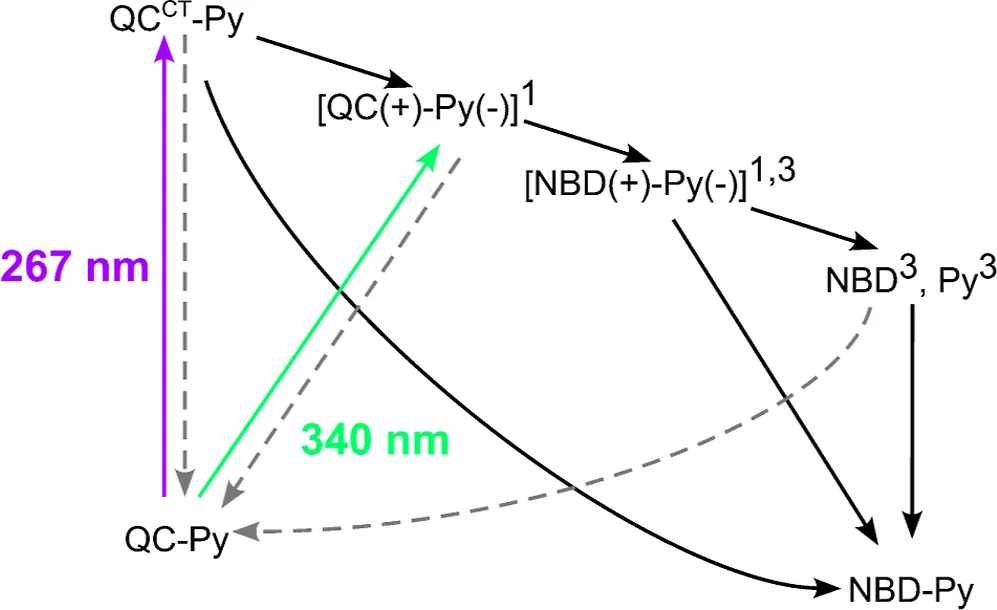
Reaction pathways of pyrene linked NBD for photoinduced QC-to-NBD isomerization. The colored arrows indicate the excitation processes with different wavelengths. The solid black arrows indicate the major productive pathways, while the gray dashed arrows depict the nonproductive pathways. The apparent rate constants are given in the Supporting Information (Figure S27).

This work provides a unified mechanistic framework for substituted norbornadienes spanning femtosecond to microsecond time scales. By directly resolving CT states, radical ion pairs, and triplet intermediates in both UV–vis and DUV regions, we demonstrate how pyrene coupling introduces a two-stage isomerization process on the picosecond and nanosecond scale with 2 distinct reaction mechanisms by directly probing the product absorptions of NBD and QC and relative excited-state species.

For molecular solar thermal energy storage, triplet-assisted pathways provide a mechanism to extend reaction time scales and enhance overall switching efficiency.^33^ For optical information storage, excitation wavelength-dependent pathway selection offers a strategy for external control over reaction directionality and kinetics. By revealing how singlet and triplet channels compete and cooperate within multichromophore NBD architectures, this study provides mechanistic guidance for the rational design of next-generation photoresponsive energy and information storage materials.

## Supplementary Material



## Notes

Synthetic procedures, NMR characterization of pyrene-linked NBDs, irradiation measurements with NMR monitoring, details on UV–vis steady-state measurements including absorption spectra measurements and photoconversion through irradiation, details on the calculation of quantum yields, explanation of the experimental setup for the transient absorption measurements, triplet state dynamics of NBD-TS with recorded difference spectra and global lifetime analysis, lifetime analysis based on the sequential model, and transient absorption data (PDF)
