# Supplementary material for: Photocontrolled Isomerization Pathways of Tolylsulfonyl- and Pyrene-Linked Norbornadiene Systems Driven by Absorption Band Targeting
Source: J Am Chem Soc. 2026 Aug 25;148(35):38500–10. doi: 10.1021/jacs.6c14776 (PMC13564436; doi:10.1021/jacs.6c14776)
Supplement: Supplementary file 1 [file ja6c14776_si_001.pdf]

Supporting Information
Photocontrolled Isomerization Pathways of Tolylsulfonyl- and Pyrene-linked Norbornadiene Systems Driven by Absorption Band Targeting

Dazhong Sun,† Daniel Krappmann,‡ Christoph Oleszak,‡ Jonas Lienert,† Chavdar Slavov,¶ Andreas Hirsch,‡ and Josef Wachtveitl*,†

†*Institute of Physical and Theoretical Chemistry, Goethe University, Max-von-Laue-Straße 7, 60438 Frankfurt am Main, Germany*

‡*Department of Chemistry and Pharmacy, Friedrich-Alexander Universität Erlangen Nürnberg, Nikolaus Fiebiger Straße 10, 91058 Erlangen, Germany*

¶*Department of Chemistry, University of South Florida, Tampa, Florida 33620, United States*

E-mail: wveitl@theochem.uni-frankfurt.de

Synthesis

All reactions involving moisture or oxygen sensitive compounds were carried out under an inert gas atmosphere of nitrogen or argon while using anhydrous solvents and standard Schlenk techniques. All used solvents were distilled on a rotary evaporator before usage. All required chemicals were purchased from commercial suppliers and used directly without

further purification. TLC analysis was performed on aluminum plates coated with 0.20 mm Merck® silica gel 60 containing a fluorescent indicator (Machery-Nagel, ALUGRAM®, SILG/UV254). Spots on TLC plates were visualized by exposure to ultraviolet light (λ = 254 nm and 366 nm). Purification via column chromatography was performed on silica gel 60M deactivated (0.04-0.063 mm / 230-400 mesh ASTM) from Macherey-Nagel®, Düren, Germany.

Flash column chromatography: Flash column chromatography was performed with a Biotage® Selekt, with the software SELEKT 1.1.1.-13044 using pre-packed columns purchase from Büchi (St. Gallen, Switzerland).

NMR: All NMR spectra were recorded on a BRUKER Avance gX (1H-NMR: 300 MHz 400 MHz; 13C NMR: 75 MHz or 100 MHz). Chemical shifts δ are reported in ppm and were referenced to the residual solvent signal as an internal reference (Table 6). Solvent impurities were determined according to the work of Fulmer et. al.

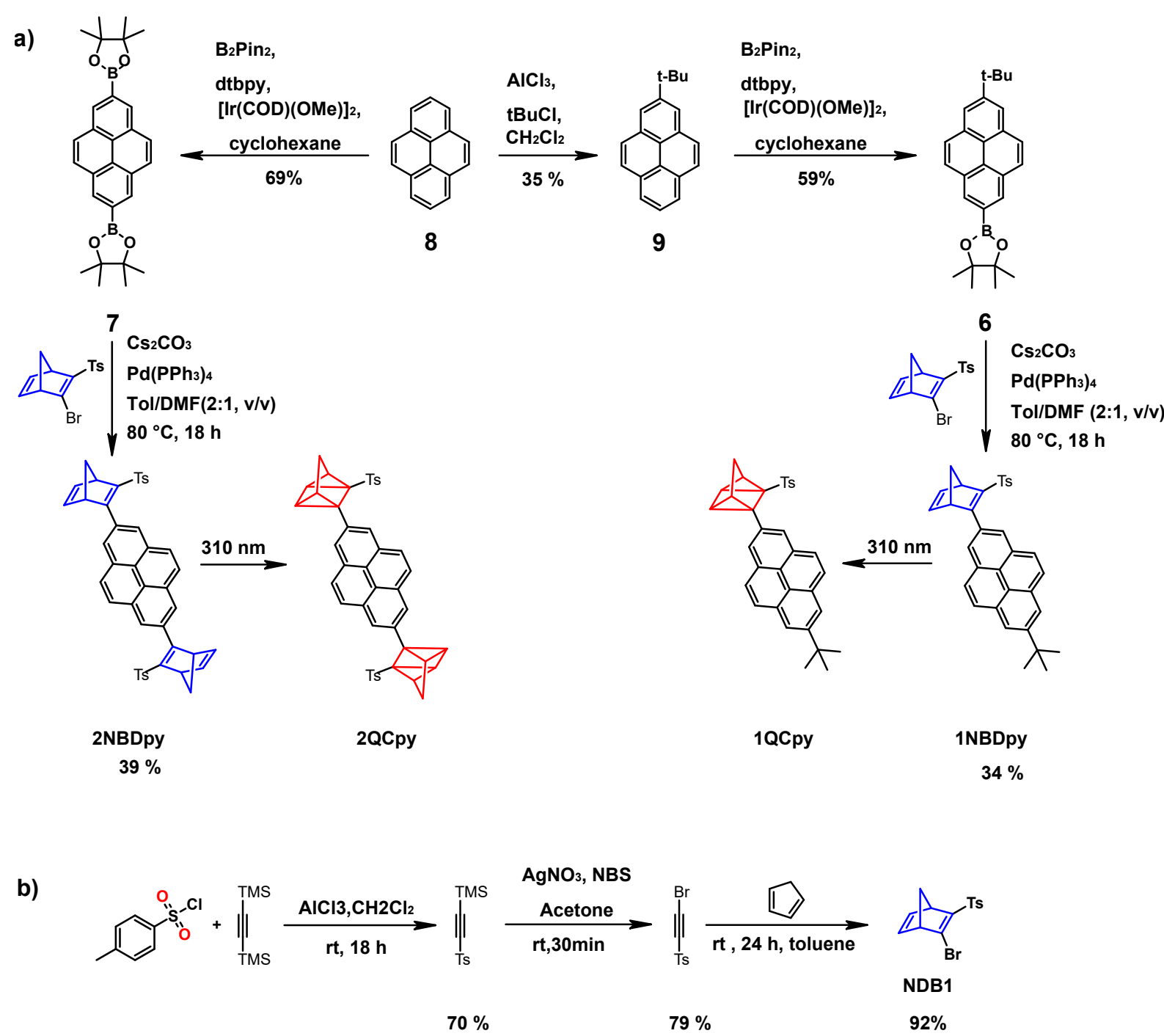

Figure S1: a) Synthesis of the precursor molecules 6 and 7 and the subsequent conversion to the target compounds 1NBDpy and 2NBDpy. b) Synthetic protocol towards cross-coupling partner NBD1.

NMR Measurements

General switching procedure A (NMR)

Photoisomerization towards molecule QC was achieved using a home-made irradiation apparatus and verified via 1H NMR spectroscopy. The respective NBD (approx. 7.5 mg) was dissolved in 650 ul of the respective deuterated solvent and transferred into a NMR test tube. One initial NMR was measured of each freshly prepared sample prior to the irradiation studies. The NMR test tube was irradiated at the given wavelength for a specific period of time. After each irradiation step, an additional 1H NMR spectrum was measured to verify the conversion. This procedure was carried on until full conversion, no further conversion or photodecomposition could be observed.

General switching procedure B (UV/Vis)

Photoisomerization towards molecule QC was achieved using a home-made irradiation apparatus. The respective NBD was dissolved in MeCN to obtain a salution which provides absoprtion less than 1 (approx. concentration of 5x10-5 M). The prepared sample was transferred into a quartz glass cuvette and irradiated using the given LED lamp for the stated period of time. UV/Vis spectra were recorded prior irradiation and after each irradiation step. This procedure was carried on until full conversion, no further conversion or photodecomposition could be observed.

NMR Data of Pyrene-linked NBDs

1NBDpy: 2-bromo-3-tosylbicyclo[2.2.1]hepta-2,5-diene[3] (50.0 mg, 154 umol, 1.0 equiv.), boronic ester 7 (59.5 mg, 154 umol, 1.0 equiv.), Cs_2CO_3 (151 mg, 462 umol, 3.0 equiv.), and $Pd(PPh_3)_4$ (8.90 mg, 7.70 umol,5 mol%) were submitted to a flame dried, Ar containing pressure tube. The vessel was sealed and the atmosphere again cycled three times. A mixture of toluene (2 mL) and DMF (1 mL) were purged with Ar for 30 min and added to the vial.

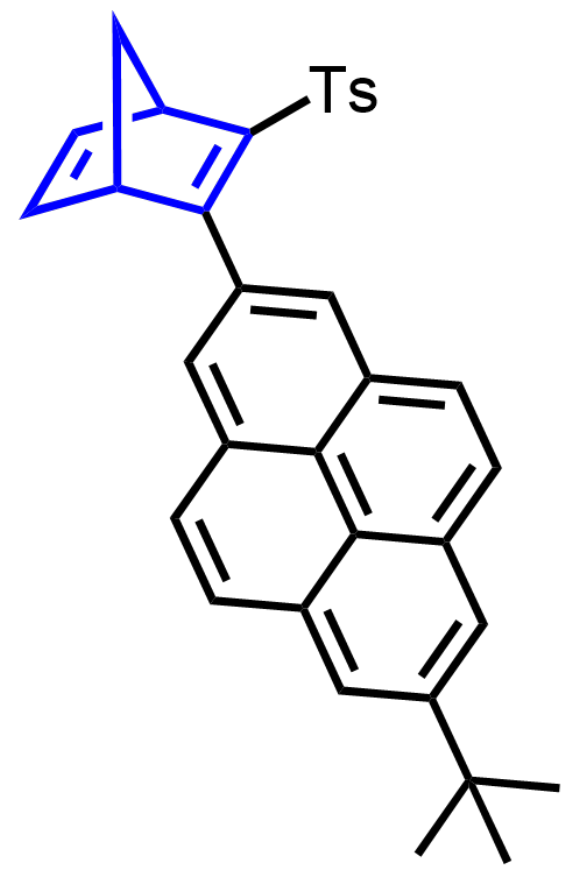

Chemical Formula: $C_{34}H_{30}O_2S$
Molecular Weight: 502.67

The obtained reaction mixture was heated to 80°C for 18 h. Afterwards, the reaction was cooled to room temperature and the solvents removed under reduced pressure. The residue was adsorbed on SiO2 and subjected to automized flash column chromatography (15% EtOAc in hexanes). Subsequent washing of the obtained solid with Et_2O and recrystallization from DCM/pentane yielded the product as pale-yellow crystals.

Yield: 26.4 mg, 52.5 umol, 34%.

Rf = 0.65 (hexanes/EtOAc 4:1)

^{1}H NMR (400 MHz, CDCl3, 25°C) δ_H [ppm]: 8.22 (s, 2H), 8.16 (s, 2H), 8.08 – 8.01 (m, 4H), 7.48 – 7.44 (m, 2H), 7.03 – 7.00 (m, 2H), 6.99 – 6.96 (m, 1H), 6.77 – 6.74 (m, 1H), 4.16 – 4.10 (m, 2H), 2.50 – 2.47 (m, 1H), 2.19 (s, 3H), 2.16 – 2.12 (m, 1H), 1.59 (s, 9H).

^{13}C NMR (101 MHz, CDCl3, 25°C): (δ) [ppm] = 164.5, 149.7, 147.3, 143.9, 143.1, 139.8, 136.9, 131.3, 131.0, 130.4, 129.4, 128.2, 127.7, 127.5, 124.8, 124.2, 122.8, 122.6, 70.6, 60.4, 54.3, 35.4, 32.1, 21.5.

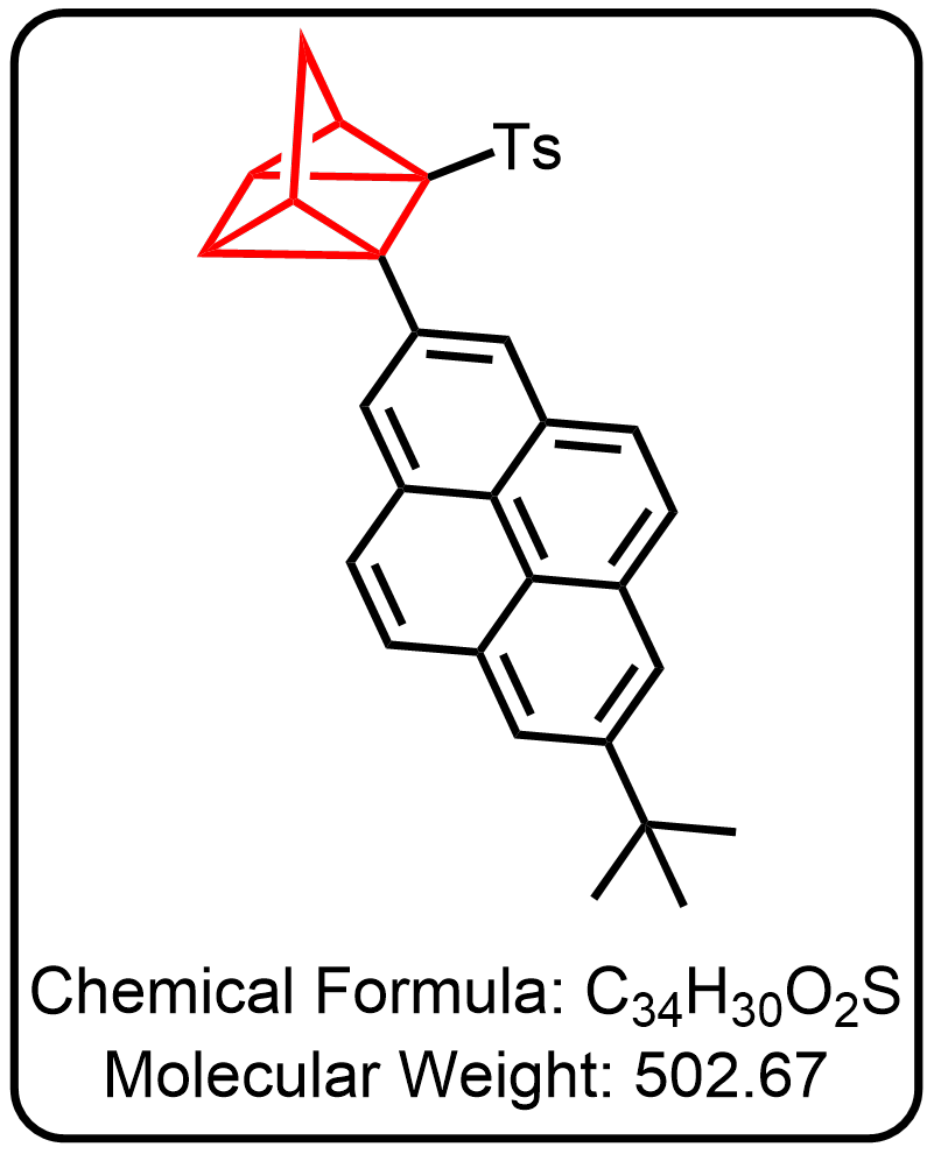

1QCpy:The conversion of 1NBDpy to 1QCpy was conducted according to general procedure A and B.

Yield (UV/Vis): assumed quantitively (MeCN, 310 nm, 30 s)

Yield (NMR): max. conversion of 84% after 110 h (CDCl3, 310 nm)

^{1}H NMR (400 MHz, CDCl3, 25°C) δ_H [ppm]: 8.22 (s, 2H), 8.08 – 7.99 (m, 3H)*, 7.86 (s, 1H), 7.84 (s, 1H), 7.74 (s, 2H), 7.09 –7.05 (m, 2H), 6.78 – 6.71 (m, 2H), 2.97 – 2.88 (m, 2H), 2.63 – 2.58 (m, 1H), 2.51 (dd, J = 4.9, 2.3 Hz, 1H), 2.31 – 2.26 (m, 1H), 2.23 (s, 3H), 1.91 – 1.85 (m, 1H), 1.60 (s, 9H). *Integration not possible due to overlapping signals. The integration value was found as 3 but must be 2.

^{13}C NMR (101 MHz, CDCl3, 25°C): (δ) [ppm] = 149.2, 143.6, 137.8, 131.2, 131.1, 130.8, 129.2, 128.0, 127.8, 127.2, 126.3, 123.8, 122.9, 122.4, 48.0, 35.4, 35.4, 34.5, 33.6, 32.1, 31.9, 27.2, 21.6, 19.7.

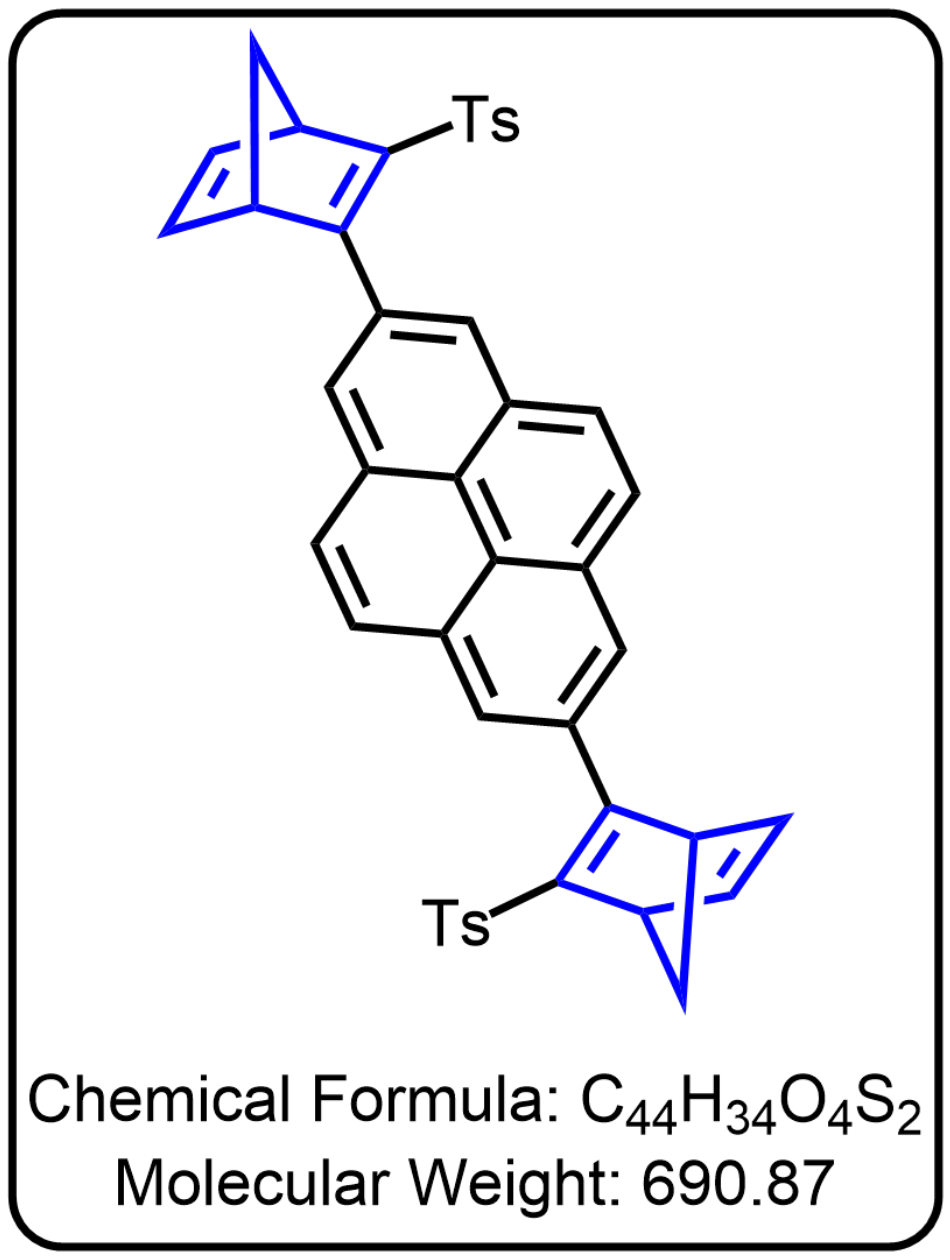

2NBDpy: In the dark, 2-bromo-3-tosylbicyclo[2.2.1]hepta-2,5-diene[3] (287 mg, 882 umol, 2.0 equiv.), boronic ester 9 (200 mg, 441 umol, 1.0 equiv.), Cs2CO3 (431 mg, 1.32 mmol, 3.0 equiv.), and Pd(PPh3)4 (25.5 mg, 22.0 umol, 5 mol%) were submitted to a flame dried, Ar containing pressure tube. The vessel was sealed and the atmosphere again cycled three times. A mixture of toluene (8 mL) and DMF (4 mL) were purged with Ar for 30 min and added to the vial. To exclude any light exposition, the pressure tube was wrapped with multiple layers of aluminum foil. The obtained reaction mixture was heated to 80°C for 18 h. Afterwards, the reaction was cooled to room temperature and the solvents removed under reduced pressure. The residue was adsorbed on SiO_2 and subjected to automized flash column chromatography (15% to 25% EtOAc in hexanes). Subsequent washing of the obtained solid with Et_2O and recrystallization from DCM/hexanes yielded the product as pale-yellow crystals. *Exclusion of any external light source during the synthesis and purification is essential to obtain the bis-NBD derivative! Exposure to ambient light leads to immediate QC formation.

Yield: pale yellow solid 119 mg, 173 umol, 39%.

Rf = 0.45 (hexanes/EtOAc 2:1)

^{1}H NMR (400 MHz, $CDCl_3$, 25 °C) δ_H [ppm]: 8.20 (s, 4H), 8.07 (s, 4H), 7.50 – 7.46 (m, 4H), 7.08 – 7.04 (m, 4H), 7.00 – 6.97 (m, 2H), 6.76 – 6.72 (m, 2H), 2.51 – 2.47 (m, 2H), 2.23 (s, 6H), 2.17 – 2.13 (m, 2H).

^{13}C NMR (101 MHz, $CDCl_3$, 25 °C): (δ) [ppm] = 164.2, 164.2, 147.7, 147.7, 144.0, 144.0, 143.2, 139.8, 139.8, 136.8, 131.9, 130.8, 129.4, 128.4, 128.1, 127.8, 124.6, 124.6, 70.7, 60.3, 54.4, 21.6.

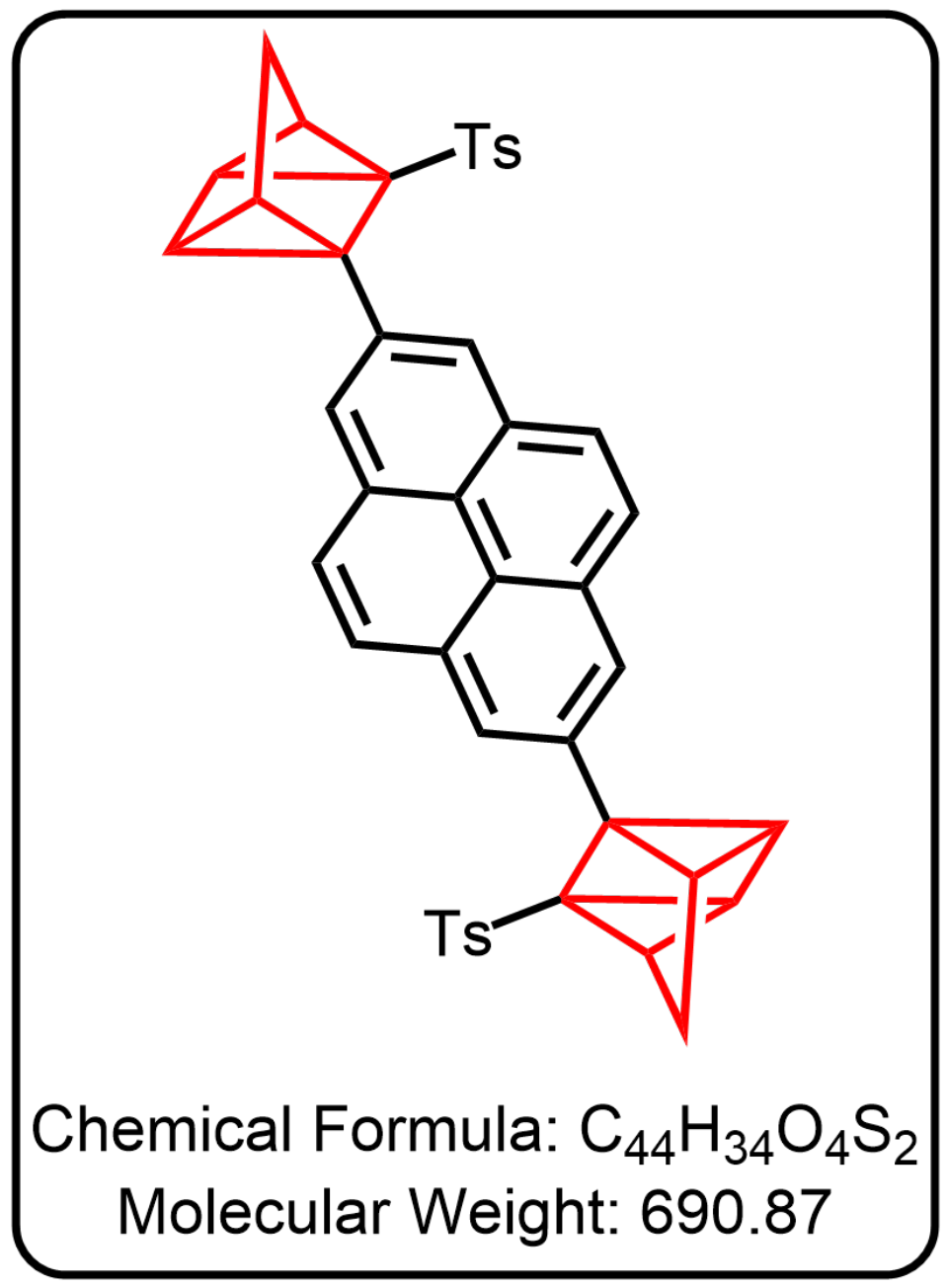

2QCpy: The conversion of PAH-NBD1 to PAH-QC1 was conducted according to general procedure A and B.

Yield (UV/Vis): assumed quantitively (MeCN, 310 nm, 10 s). Prolonged irradiation led to photodecomposition.

Yield (NMR): 68% (toluene-d8, 310 nm)

^{1}H NMR (400 MHz, $CDCl_3$, 25 °C) δ_H [ppm]: 7.86 (s, 4H), 7.78 (d, J = 1.0 Hz, 4H), 7.13 – 7.09 (m, 4H), 6.81 – 6.78 (m, 5H), 2.96 – 2.92 (m, 4H), 2.63 – 2.62 (m, 1H), 2.60 – 2.59 (m, 1H), 2.56 – 2.53 (m, 2H), 2.32 – 2.31 (m, 1H), 2.29 – 2.28 (m, 1H), 2.26 (s, 6H), 1.92 – 1.89 (m, 2H).

^{13}C NMR (101 MHz, $CDCl_3$, 25 °C): (δ) [ppm] = 143.7, 137.9, 131.8, 130.9, 129.2, 129.2,

128.0, 127.4, 126.6, 126.5, 123.7, 48.0, 35.5, 34.5, 33.7, 33.7, 31.9, 27.2, 21.6, 19.9, 19.8.

Charaterization

1NBDpy

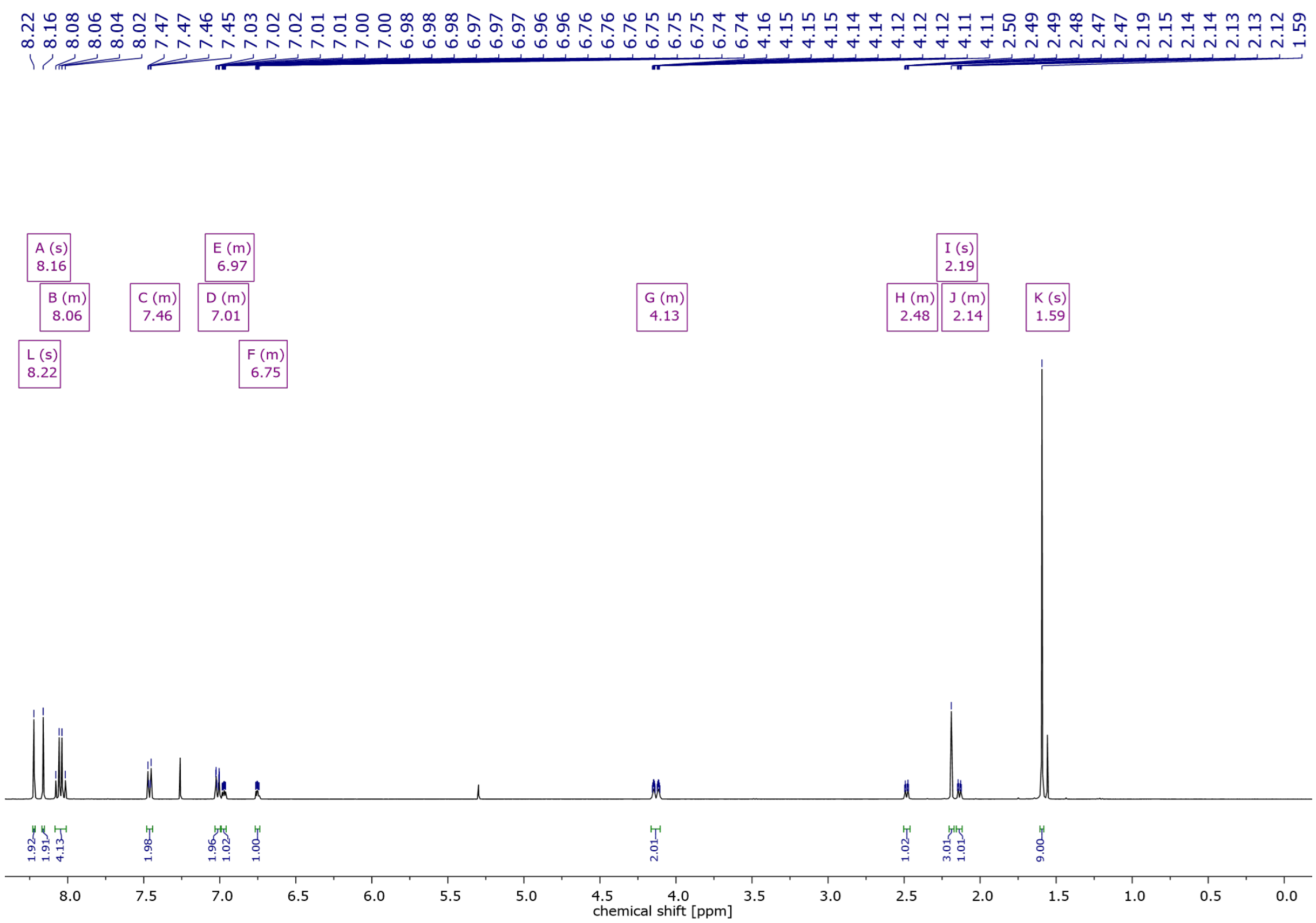

Figure S2: 1H NMR spectrum of 1NBDpy measured in $CDCl_3$ (400 MHz, rt).

1QCpy Formation by illumination

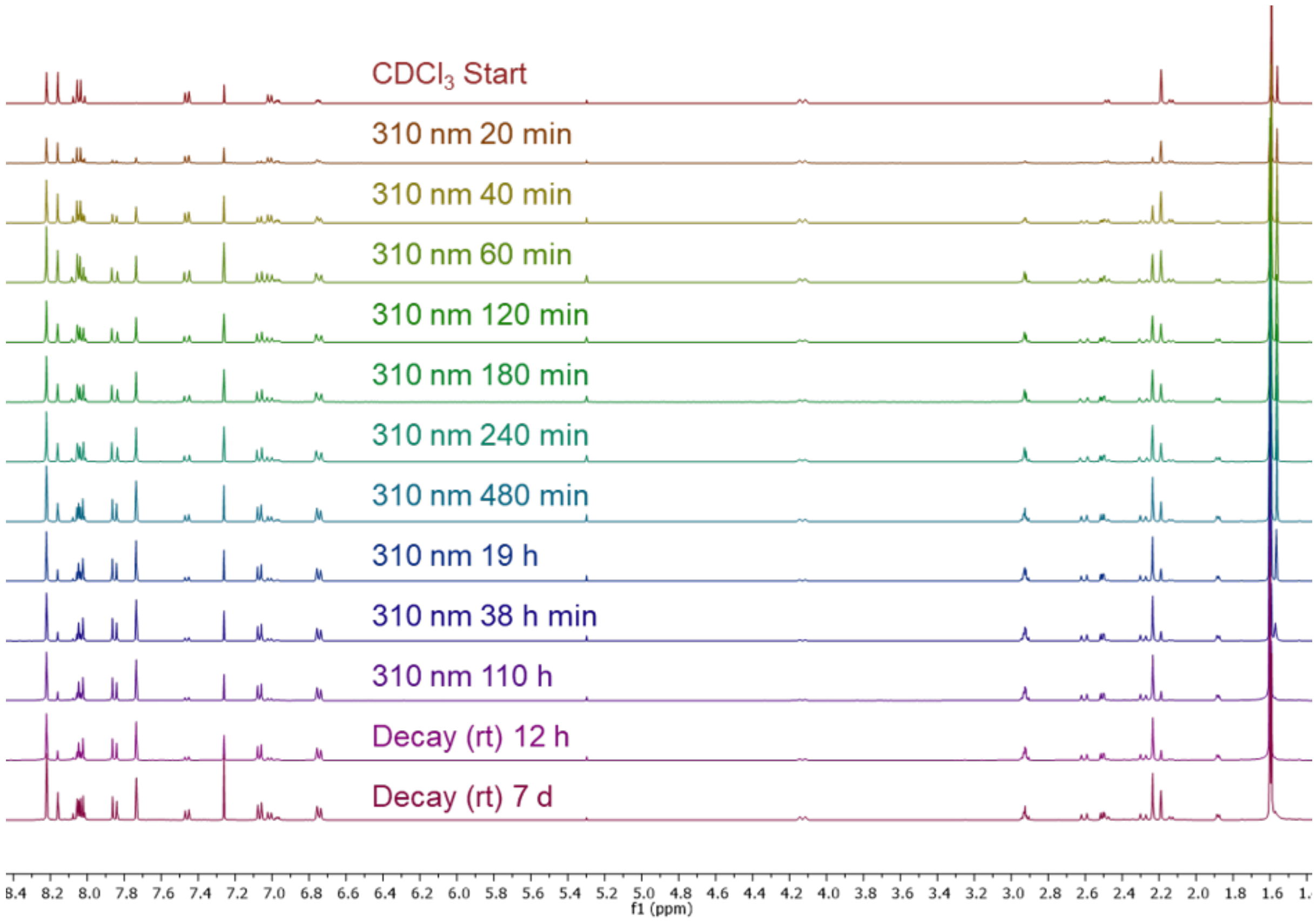

Figure S3: Interconversion of 1NBDpy to 1QCpy measured in $CDCl_3$ (400 MHz). For irradiation, a 310 nm LED was utilized. After 110h illumination, the sample was left to rest at ambient condition, partially inducing the back-conversion after 7 days at rt.

Table S1: NMR conversion of 1NBDpy to 1QCpy using $CDCl_3$ as solvent.

Irradiation time	QC [%]
0	22
20 min	37
40 min	48
60 min	59
120 min	64
240 min	67
480 min	70
19 h	79
38 h	83
110 h	84
12 h @ rt	82
7 d @ rt	62

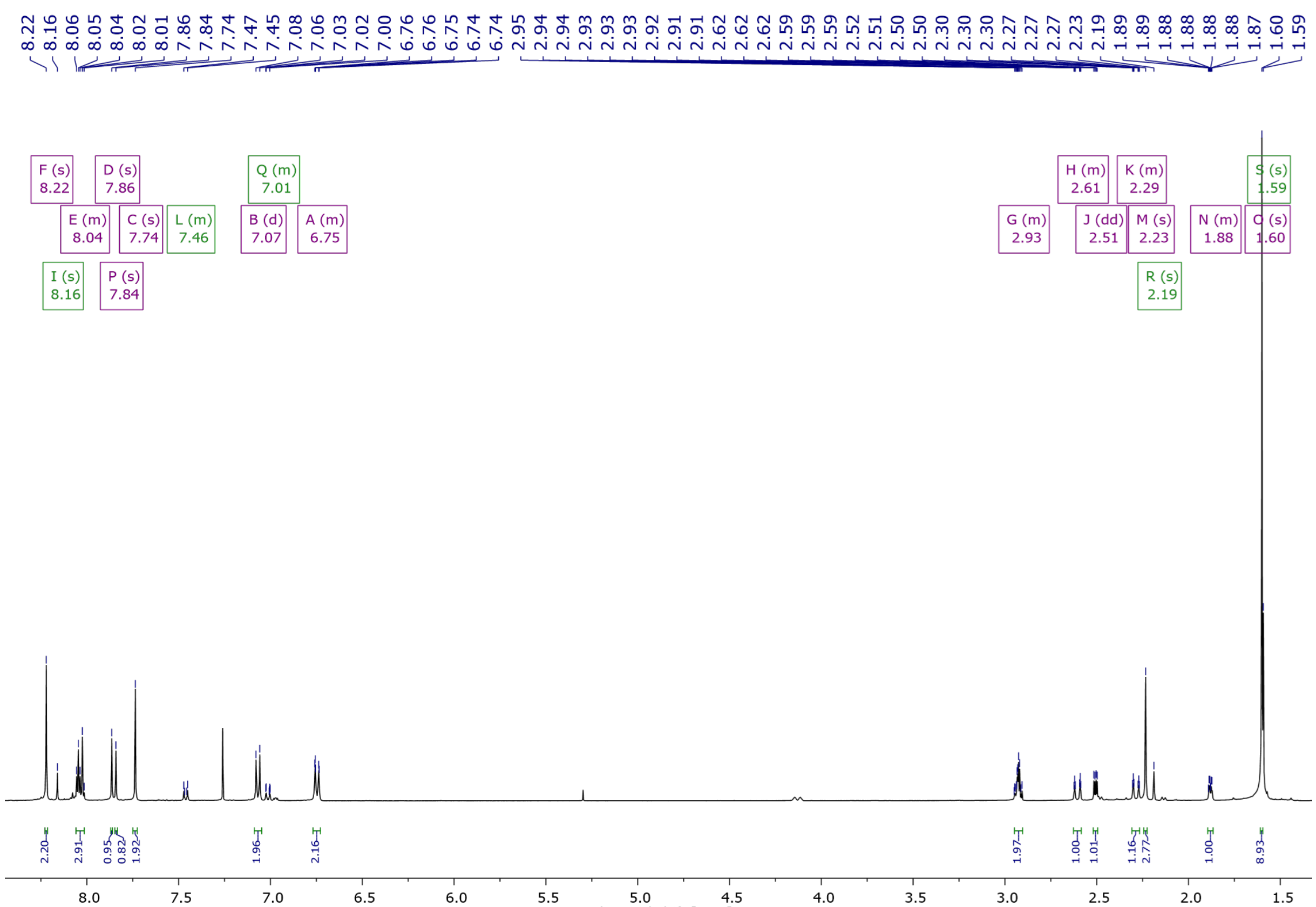

Figure S4: 1H NMR spectrum of 1QCpy measured in $CDCl_3$ (400 MHz, rt). Residual signals of the leftover PAH-NBD1 species can be found disturbing proper integration (signals highlighted in green).

2NBDpy

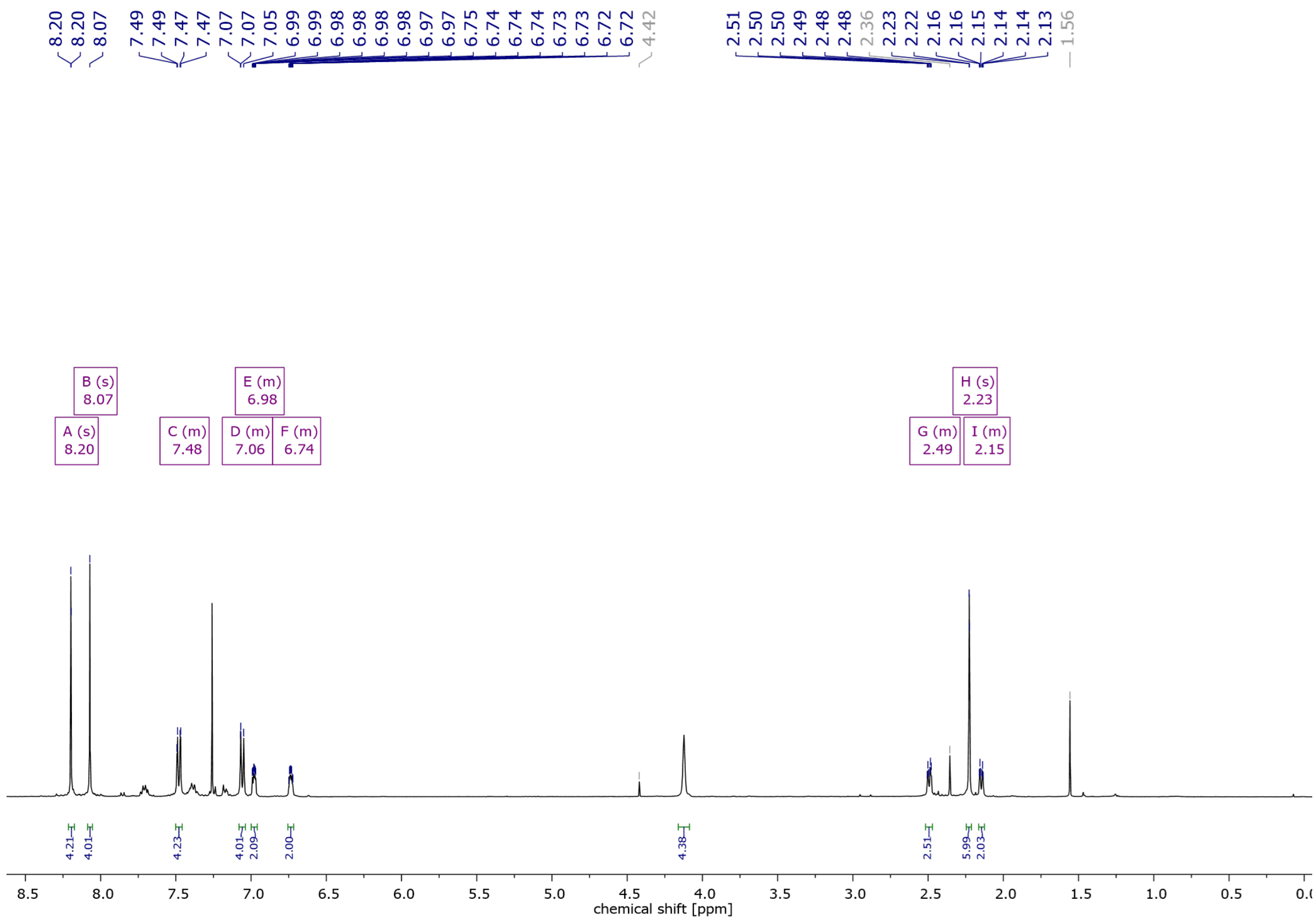

Figure S5: 1H NMR spectrum of 2NBDpy measured in $CDCl_3$ (400 MHz, rt).

2QCpy Formation by illumination

Depending on the resolution of the spectrum and the presence of 2 diastereoisomers of 2NBDpy which were not separated, some of the signals are split to 2 signals per carbon atom. In most cases, both signals are overlapping resulting in a single signal.

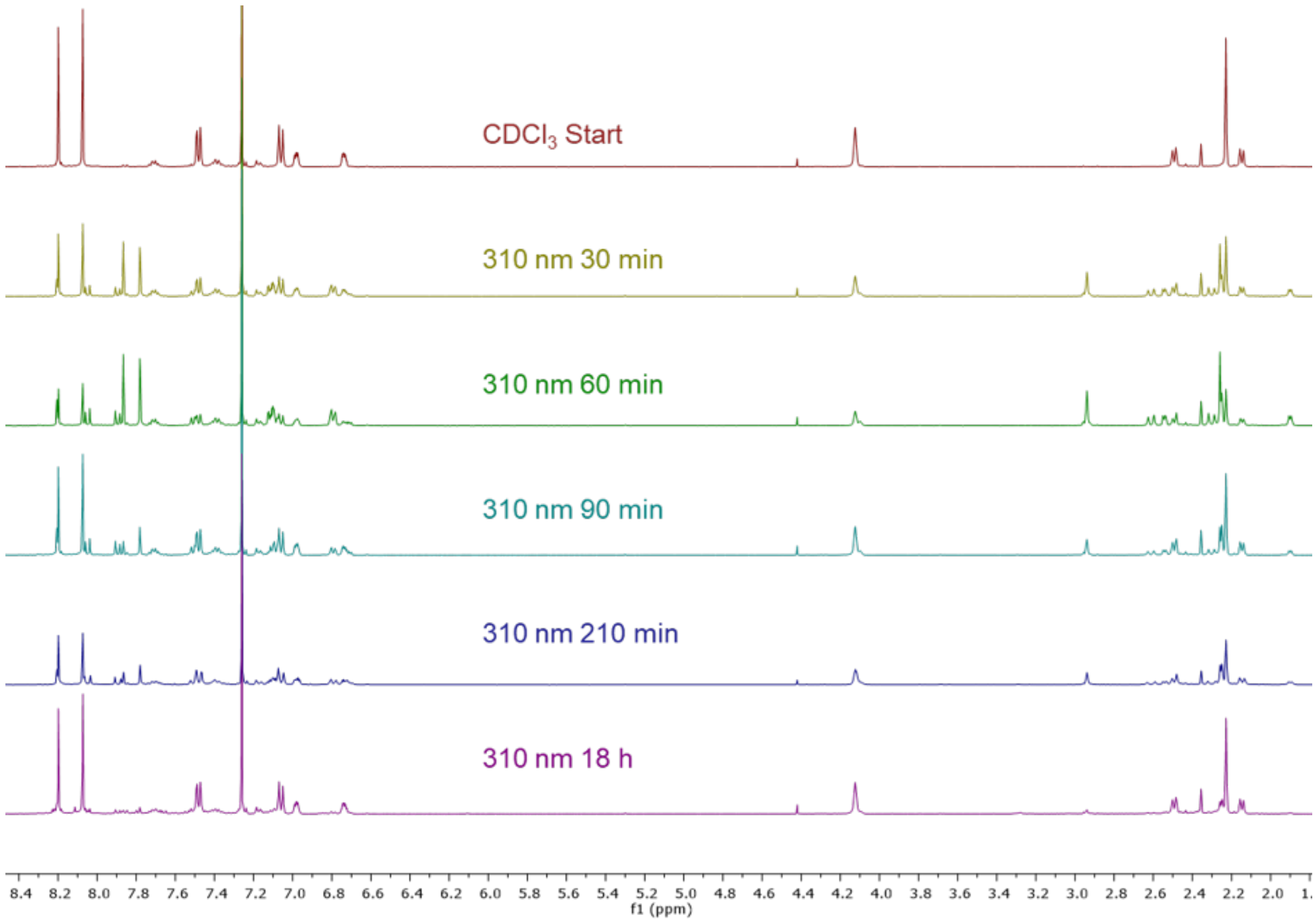

Table S2: NMR conversion of 2NBDpy to 2QCpy using $CDCl_3$ as solvent.

**Irradiation time**	**QC (%)**
0	0
30 min	42
60 min	57
90 min	24
210 min	30
18 h	10

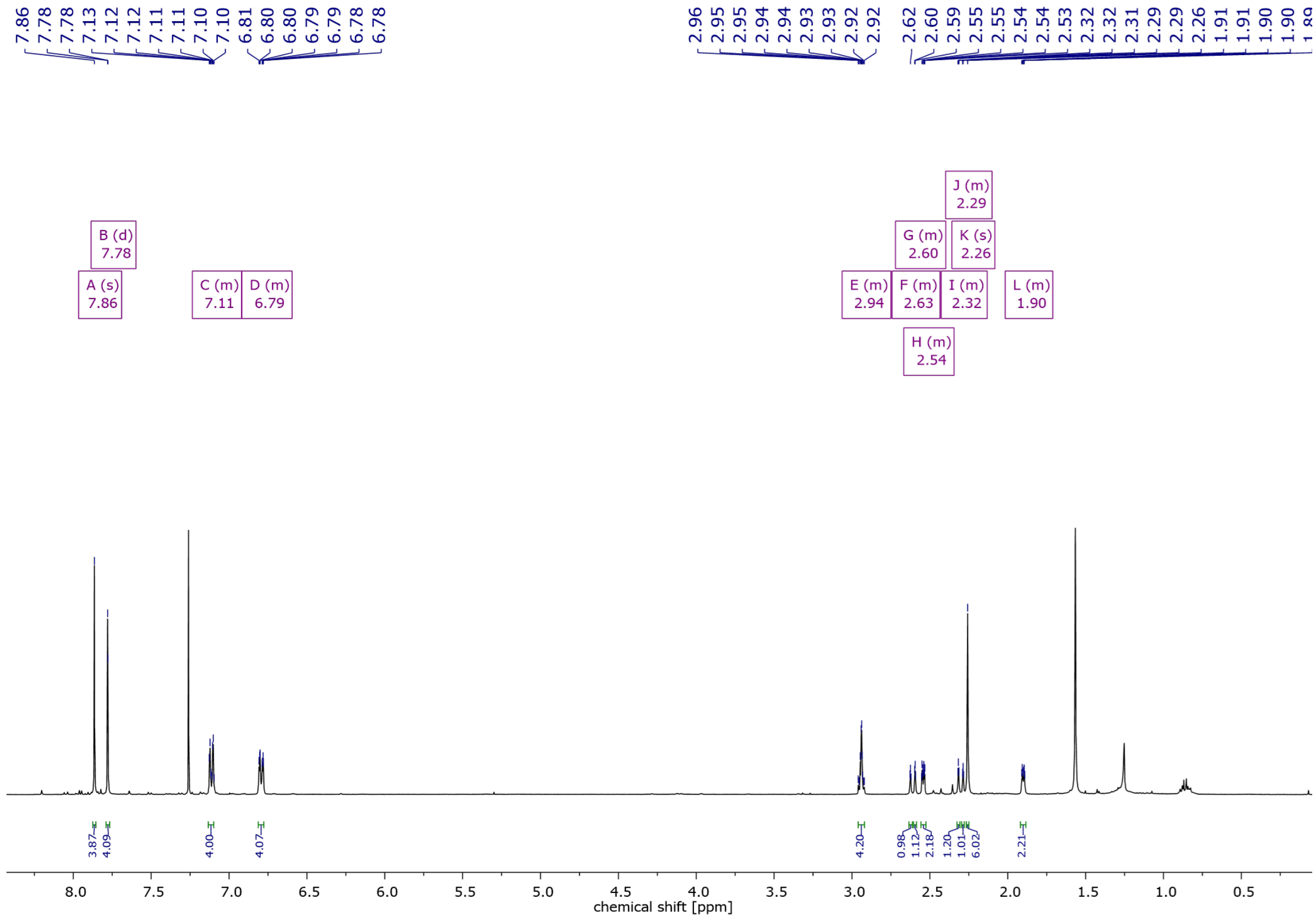

Figure S6: 1H NMR spectrum of 2QCpy in$CDCl_3$ ([400 MHz, rt])

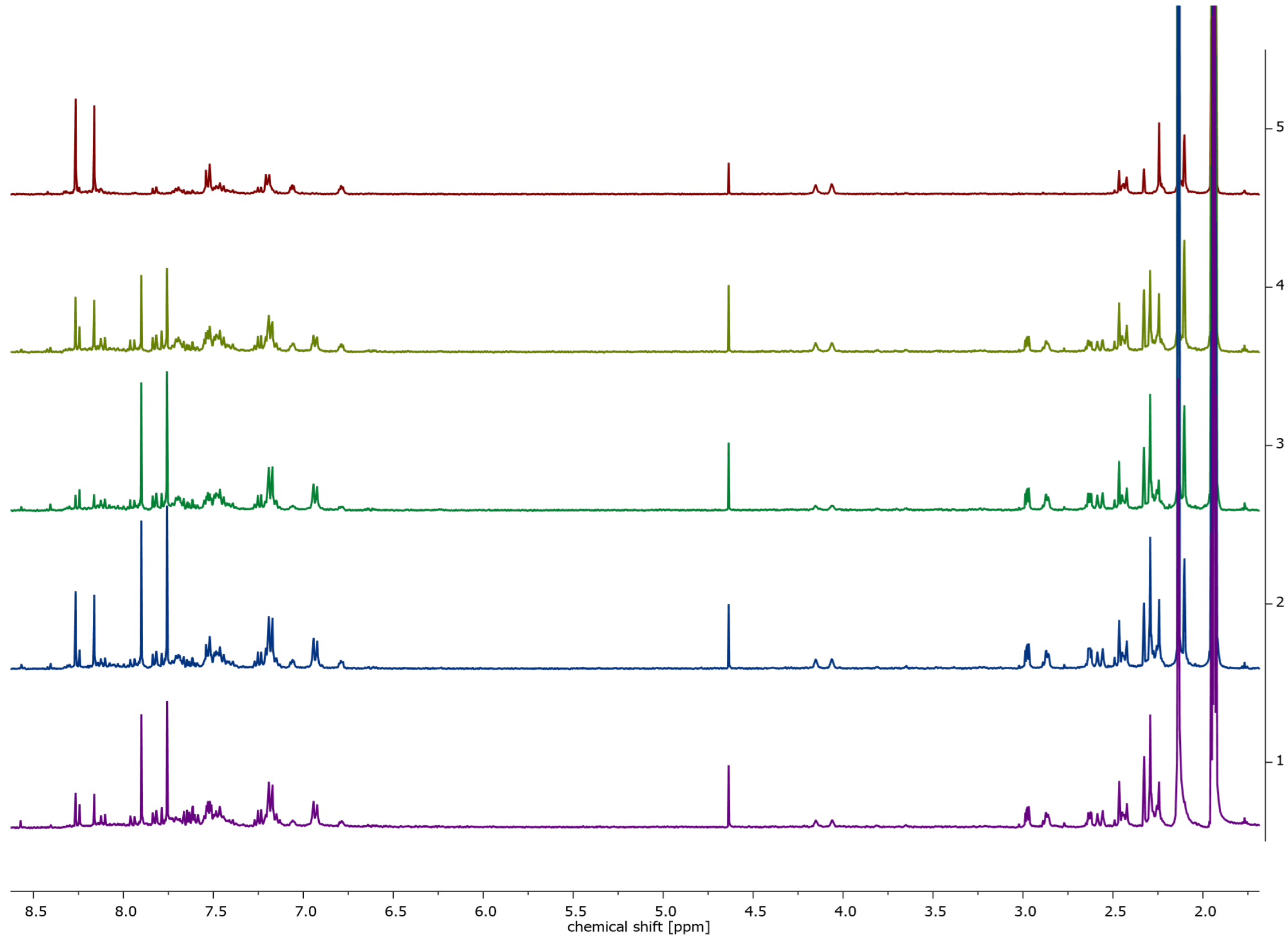

Figure S7: 1H NMR spectrum of 2NBDpy to 2QCpy in MeCN$-$d$_3$. For the conversion a 310 nm LED was used. The respective spectra can be assigned as follows: 5 = initial NBD spectrum, 4 = after 30 minutes irradiation, 3 = after 60 minutes irradiation, 2 = after 90 minutes irradiation, 1 = after 18h irradiation. During the irradiation experiment, massive precipitation occurred resulting in the worse resolution over time.

In the irradiation experiments with 2NBDpy in CDCL$_3$, clear photodegradation was observed after long exposure time (Table S3). Thus, experiments in acetonitrile was carried out, however, smilar degradation was observed.

Table S3: NMR conversion of 2NBDpy to 2QCpy using MeCN$-$d$_3$ as solvent.

**Irradiation time**	**QC (%)**
0	0
30 min	52
60 min	67
90 min	61
18 h	62

UV-Vis Steady States Measurements

Absorption Spectrum and illumination Experiments

For determining the absorption spectra of the presented all norbornadienes and quatricyclanes, a Specord S600 from Analytik Jena was used, operated with the accompanying Specord software (Version 2.0.2). The wavelength range covered was from 180 nm to 960 nm. The illumination experiments were conducted with LEDs from Thorlabs (M265L5, M300L4, M340L4 for 265 nm, 300 nm and 340 nm irradiation). The samples were prepared in a 1 cm x 1 cm x 4 cm quartz cuvette in acetonitrile. In contrast to the NMR measurements, no clear signs of degradation or precipitation were observed in the UV-Vis measurements, as the experiment was conducted at a far lower concentration and power within a considerably shorter time frame.

Quantum Yield Measurements

The quantum yield measurements were conducted with spectroscopic methods as described in the reports of Stranius et al.[1] All the samples were prepared in the same 1 cm x 1 cm x 4 cm quartz cuvette in acetonitrile in a volume of 3 mL. The optical density (OD) was adjusted to be around 1 at the excitation wavelength (λ_{ex}) The samples were illuminated from top of the cuvette to reach a optical depth of 3 cm, which equaled to the absorption $A_0 = 3$.

The quantum yield is calculated acorrding to Eq.1 by measuring decrease of the NBD absorption band $\Delta\mathrm{OD}_{(\lambda_{\mathrm{detect}})}$ at 310 nm, as clean conversion from NBD to QC and vise versa are confirmed by isosbestic points and the NMR measurements. The volume V is 3 mL and power P is in the range of microwatt to ensure linear change of the ΔOD at the beginning of illumination.

$$\Phi = \frac{N_A \cdot V \cdot \Delta\mathrm{OD}_{(\lambda_{\mathrm{detect}})} \cdot h \cdot c}{\varepsilon_{(\lambda_{\mathrm{detect}})} \cdot l \cdot P \cdot (1 - 10^{-A_0}) \cdot t \cdot \lambda_{(\lambda_{\mathrm{ex}})}} \tag{1}$$

N_A: Avogadro constant

V: Volume

ΔOD: Change in optical density

h: Planck constant

c: Speed of light

ε: Molar extinction coefficient

l: Path length

P: Light power

t: Irradiation time

λ_{detect}: Detection wavelength

λ_{ex}: Excitation wavelength

NBD-TS

The quantum yield for the isomerization from NBD-Ts to QC-Ts were measured by irradiating the sample with 340 nm LED (Figure S8, left). The isosbestic points were well observed, so the the formation of QC was equivalent to the absence of NBD. The decrese of the population of the NBD form is monitored by the change of the absorption at 343 nm(extinction coefficient $\varepsilon = 16400 \mathrm{M}^{-1} \cdot \mathrm{cm}^{-1}$) according to the Lambert-Beer law. Only the early 9 points after irradiation were applied to fit Eq.1 to ensure little absorption from QC molecules, thus a linear fit could be used to estimate the quantum yields (Figure S8, right).

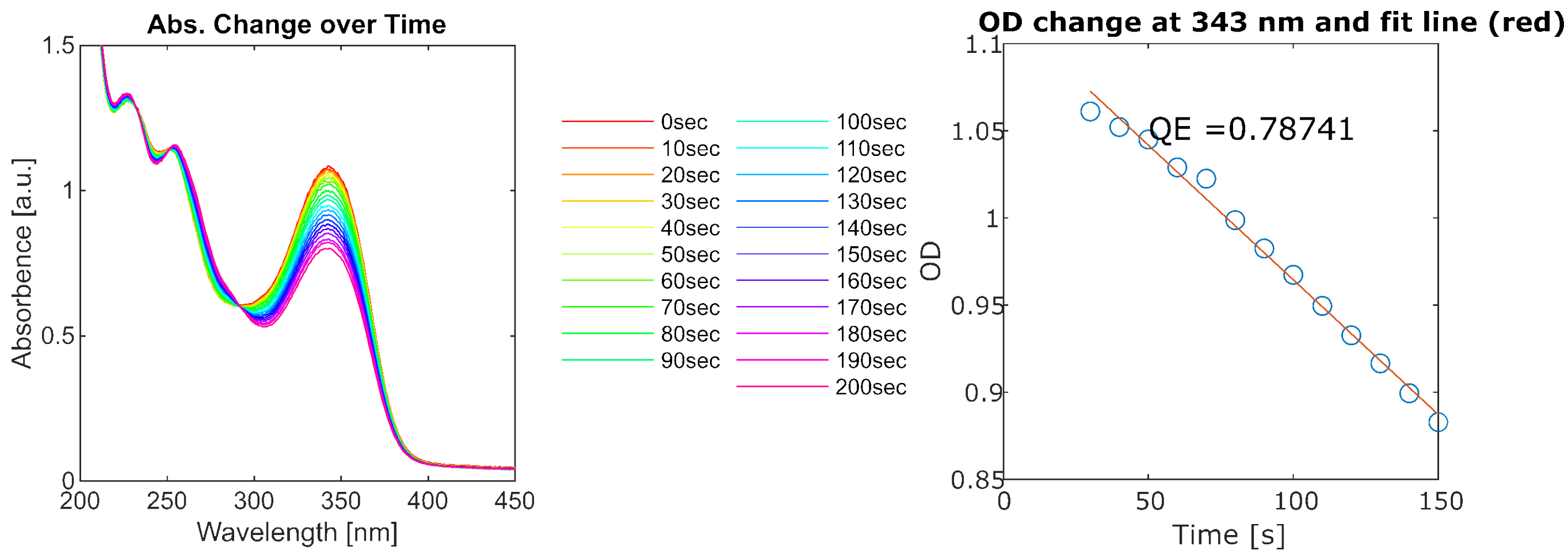

Figure S8: Absorbence change for quantum yield measurement for NBD-Ts to QC-Ts isomerization with illumination power of 0.128 mW at 340 nm. The first 9 points were chosen for linear regression.

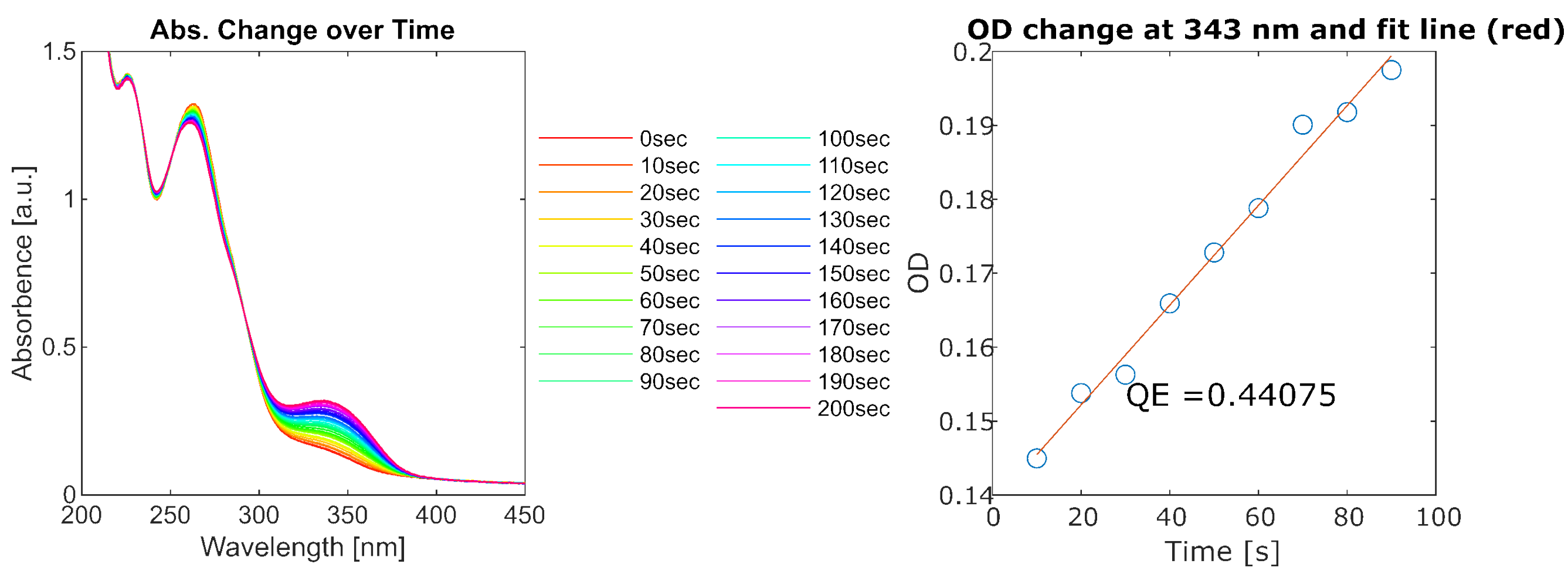

Figure S9: Absorbence change for quantum yield measurement for QC-Ts to NBD-Ts isomerization with illumination power of 0.134 mW at 265 nm. The first 9 points were chosen for linear regression.

1NBDpy

The quantum yield for the isomerization from 1NBDpy to 1QCpy were measured by irradiating the sample with 300 nm LED (Figure S10, left). The isosbestic points were well observed, so the the formation of QC was equivalent to the absence of NBD. The decrese of the population of the NBD form is monitored by the change of the absorption at 304 nm(extinction coefficient $\varepsilon = 43100\text{M}^{-1} \cdot \text{cm}^{-1}$) according to the Lambert-Beer law. Only the early 9 points after irradiation

were applied to fit Eq.1 to ensure little absorption from QC molecules, thus a linear fit could be used to estimate the quantum yields (Figure S10, right).

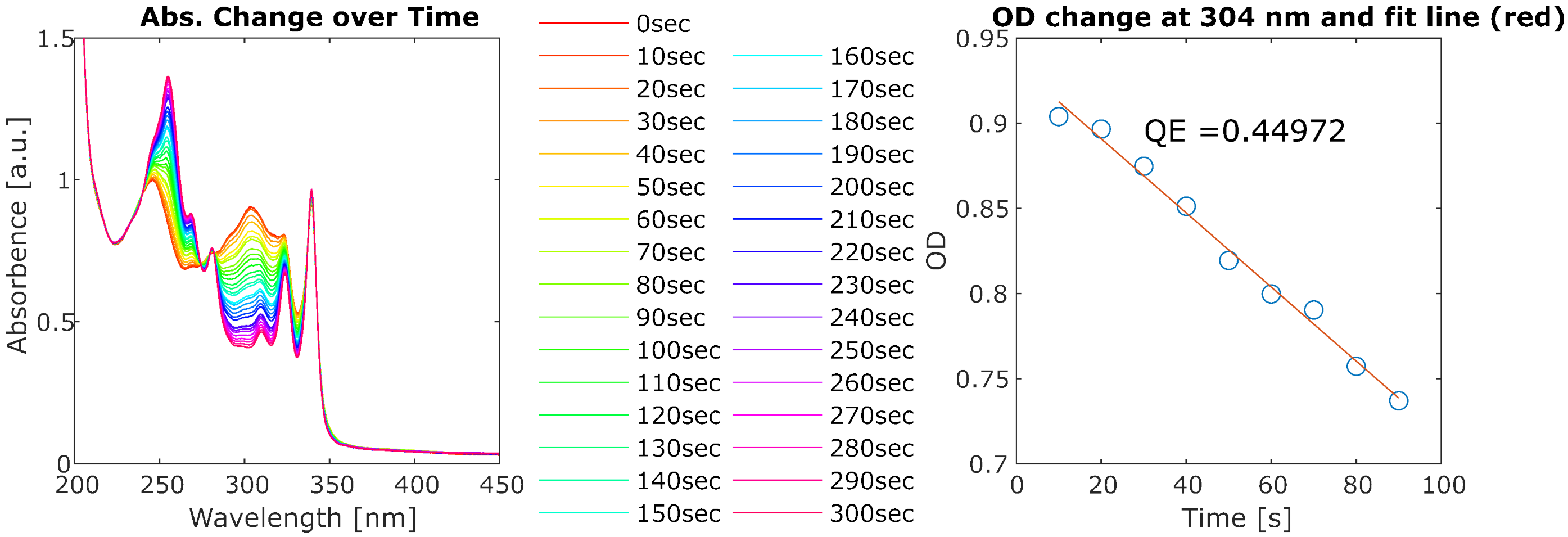

Figure S10: Absorbence change for quantum yield measurement for 1NBDpy to 1QCpy isomerization with illumination power of 0.135 mW at 300 nm. The first 9 points were chosen for linear regression.

The quantum yields for the backsiomerization were investigated with irradiation wavelength of 340 nm (Figure S11)and 265 nm (Figure S12). 1NBDpy was first irradiated with 300 nm LED to reach the photostationary state to maximize the population of the QC form and minimize the NBD absorption. Again the begingning 9 points were taken to calculated for quantum yield. The gain of NBD population is monitored through absorpton at 304 nm again.

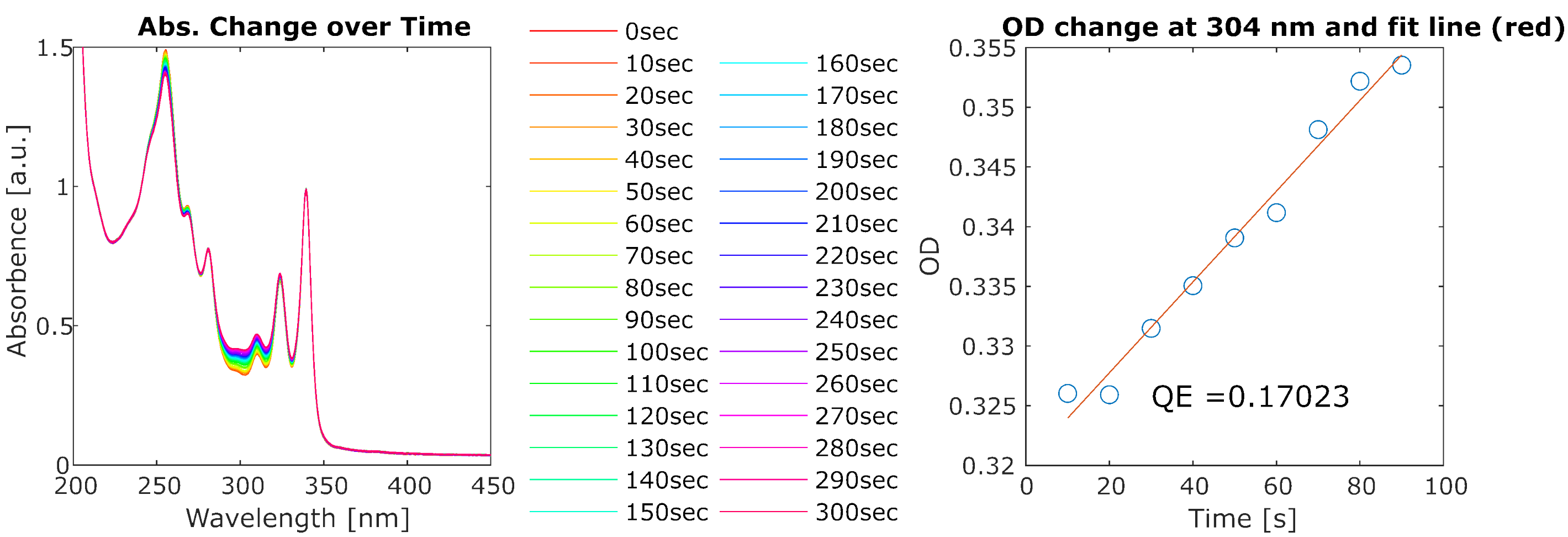

Figure S11: Absorbence change for quantum yield measurement for 1QCpy to 1NBDpy isomerization with illumination power of 0.05 mW at 340 nm. The first 9 points were chosen for linear regression.

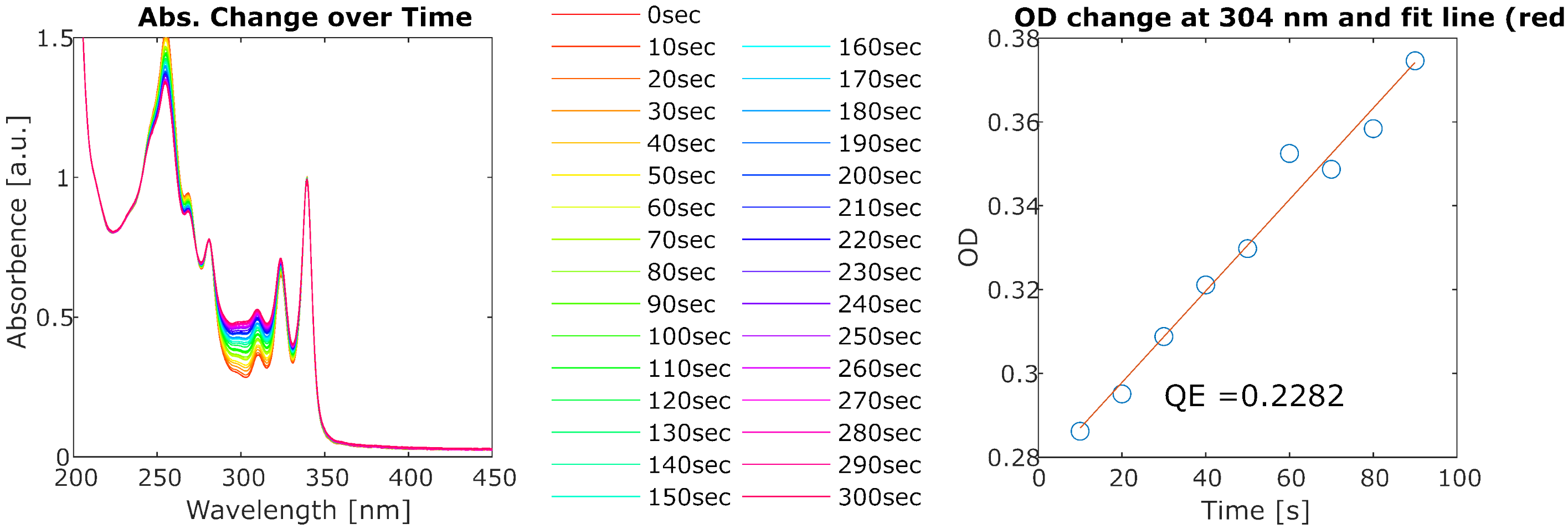

Figure S12: Absorbence change for quantum yield measurement for 1QCpy to 1NBDpy isomerization with illumination power of 0.55 mW at 265 nm. The first 9 points were chosen for linear regression.

2NBDpy

The quantum yield measurements for the forward (NBD to QC) and back (QC to NBD) isomerization were measured with the same manner as for 1NBDPy. As no clean isosbestic points were observed (Figure S13) due to i) 2 possible QC forms (NBD-py-QC and QC-py-QC) ii) possible degradation/precipitation as observed in NMR experiments with long exposure. Hence, the quantum yield measurements were using as purely reference assuming one-to-one transition is achieved. The results show that, in every situation the quantum yields are for both forward and backward isomerization lower than the case of the 1NBDpy, which is to be expected as the CT productive pathway is restricted due to the symmetrical geometry of 2NBDpy. The increase in quantum yield upon 265 nm excitation (compared to 340 nm excitation) is similar compared to 1NBDpy, which is in good agrement that the addtional quantum yields are both originated from the QC charge transfer pathway. Since excitation on pyrene (340 nm) in 2QCpy barely induced the CT pathway, the wavelength selectivity between 340 nm (pyrene) and 265 nm (QC) for 2QCpy is more pronounced.

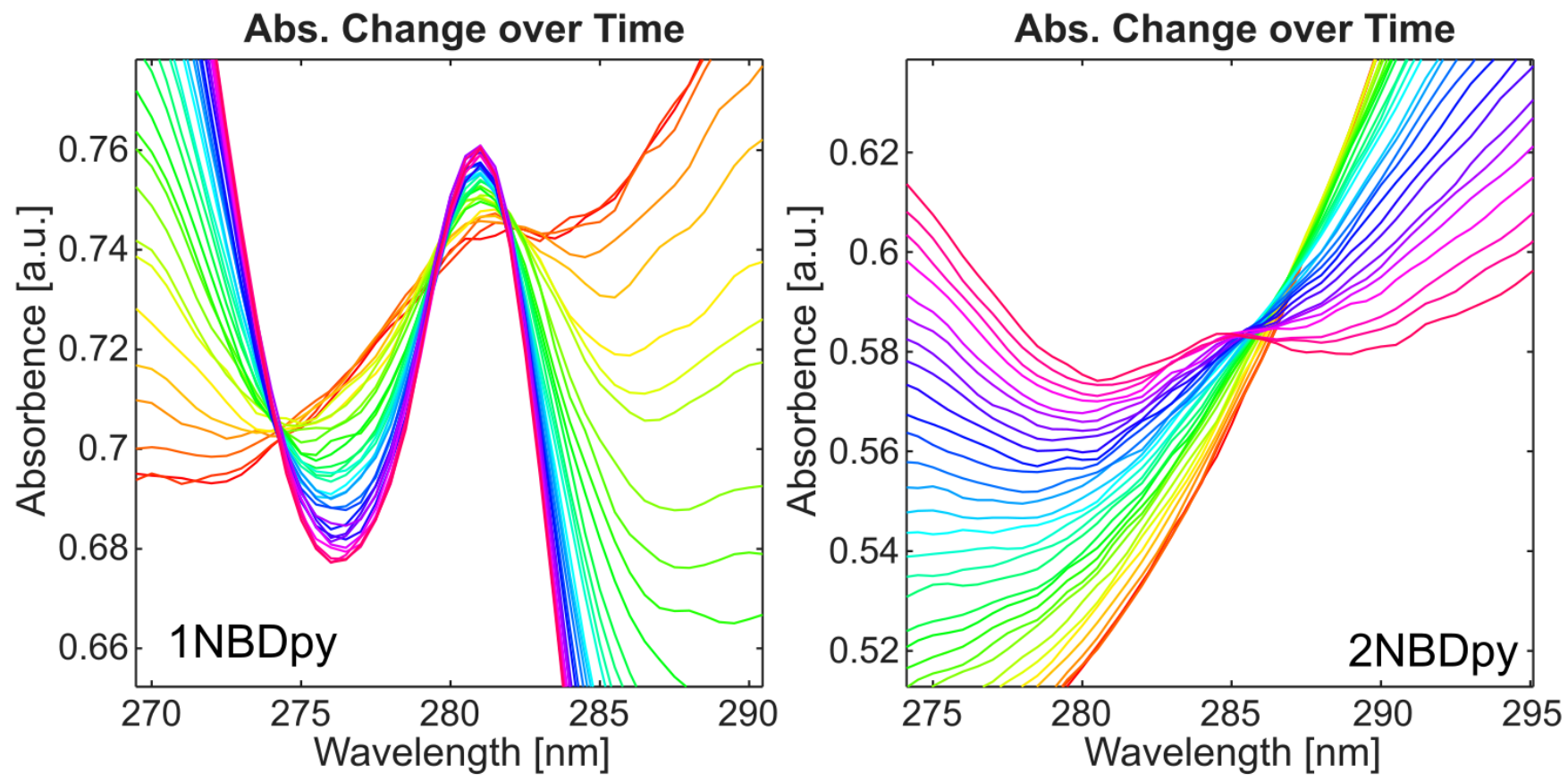

Figure S13: Absorbance changes of 1NBDpy and 2NBDpy with 300 nm irradiation(zoom-in).

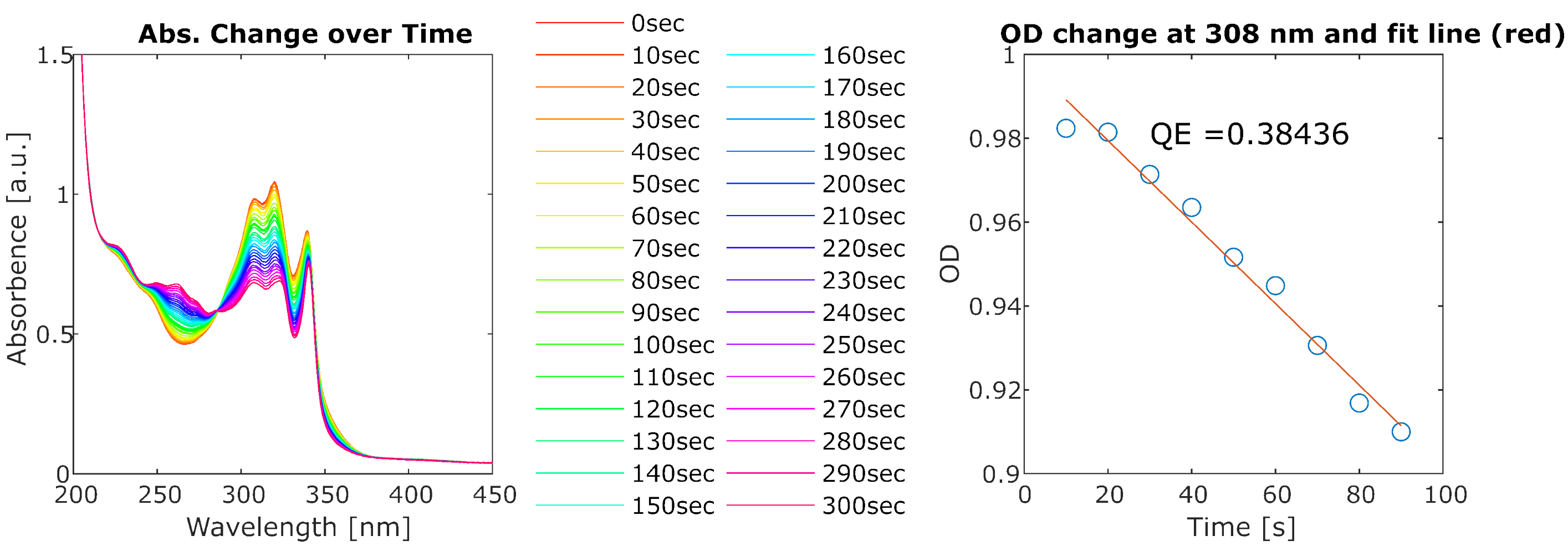

Figure S14: Absorbence change for quantum yield measurement for 2NBDpy to 2QCpy isomerization with illumination power of 0.081 mW at 300 nm. The first 9 points were chosen for linear regression.

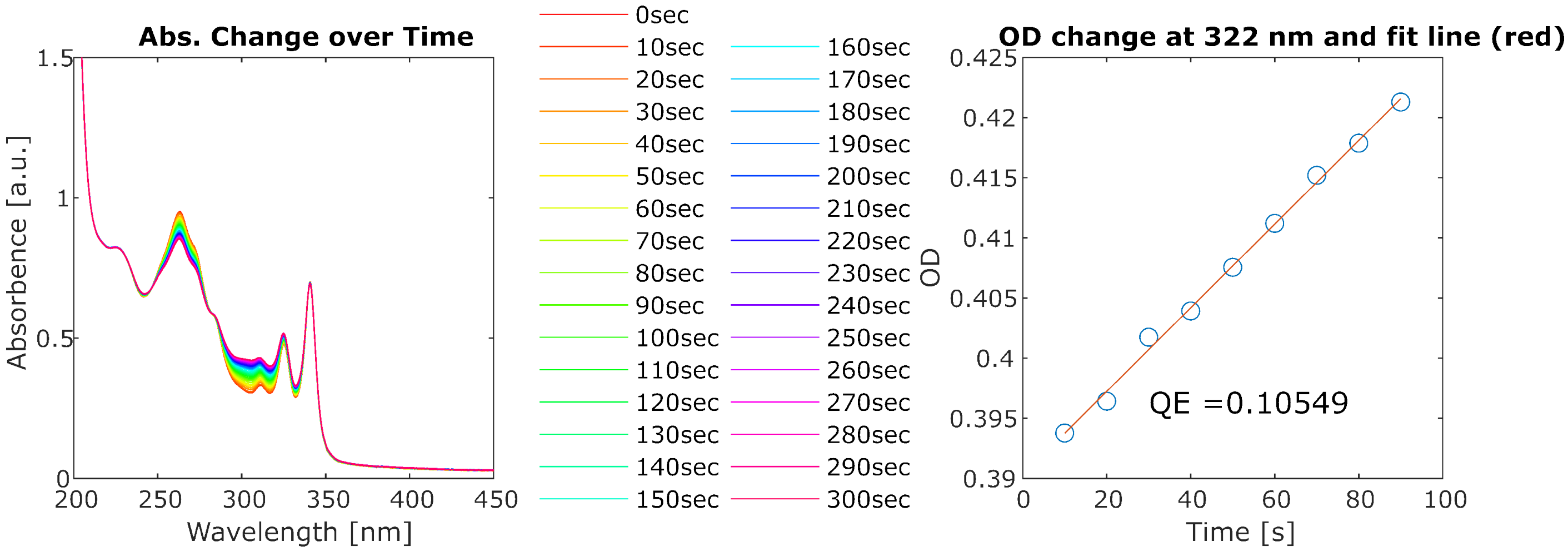

Figure S15: Absorbence change for quantum yield measurement for 2QCpy to 2NBDpy isomerization with illumination power of 0.092 mW at 340 nm. The first 9 points were chosen for linear regression.

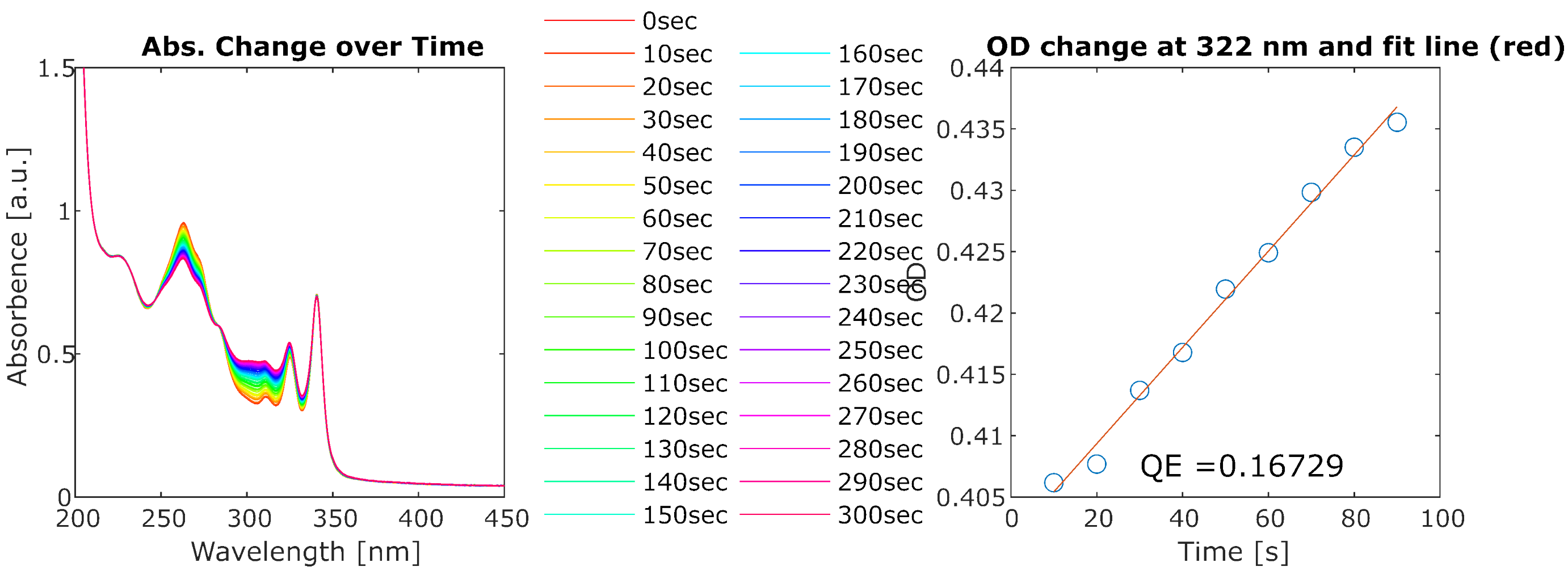

Figure S16: Absorbence change for quantum yield measurement for 2QCpy to 2NBDpy isomerization with illumination power of 0.084 mW at 265 nm. The first 9 points were chosen for linear regression.

Table S4: Summary of excitation-dependent productive channels for 1NBDpy and 2NBDpy. The number of plus sign gives a overall understanding how much the product formation relys on certain channel but does not represents absolute values. CT: charge transfer; RIP: radical ion pair.

1NBDpy	2NBDpy	NBD to QC		QC to NBD			
		308 nm		340 nm		265 nm	
Electronic promotion		CT-absorption		Pyrene absorption		CT absorption	
Quantum yield		45%	38%	17%	11%	23%	17%
productive channel	CT (fs to ps)	++++	+	++++	+	+++++	++
	RIP-triplet (ns)	++	++++	+	+++	+	+++

Emission of Pyrene-lined NBDs

The emission spectra of 1NBDpy primarily present 3 iconic features: i) very weak emission from NBD-CT state(300-350 nm); ii) noticeable emission from pyrene (around 400 nm); iii) strong excimer emssion from pyrene around 460 nm (Figure S17, C). However, for 2NBDpy, the excimer emission dominants the fluorescence spectrum.

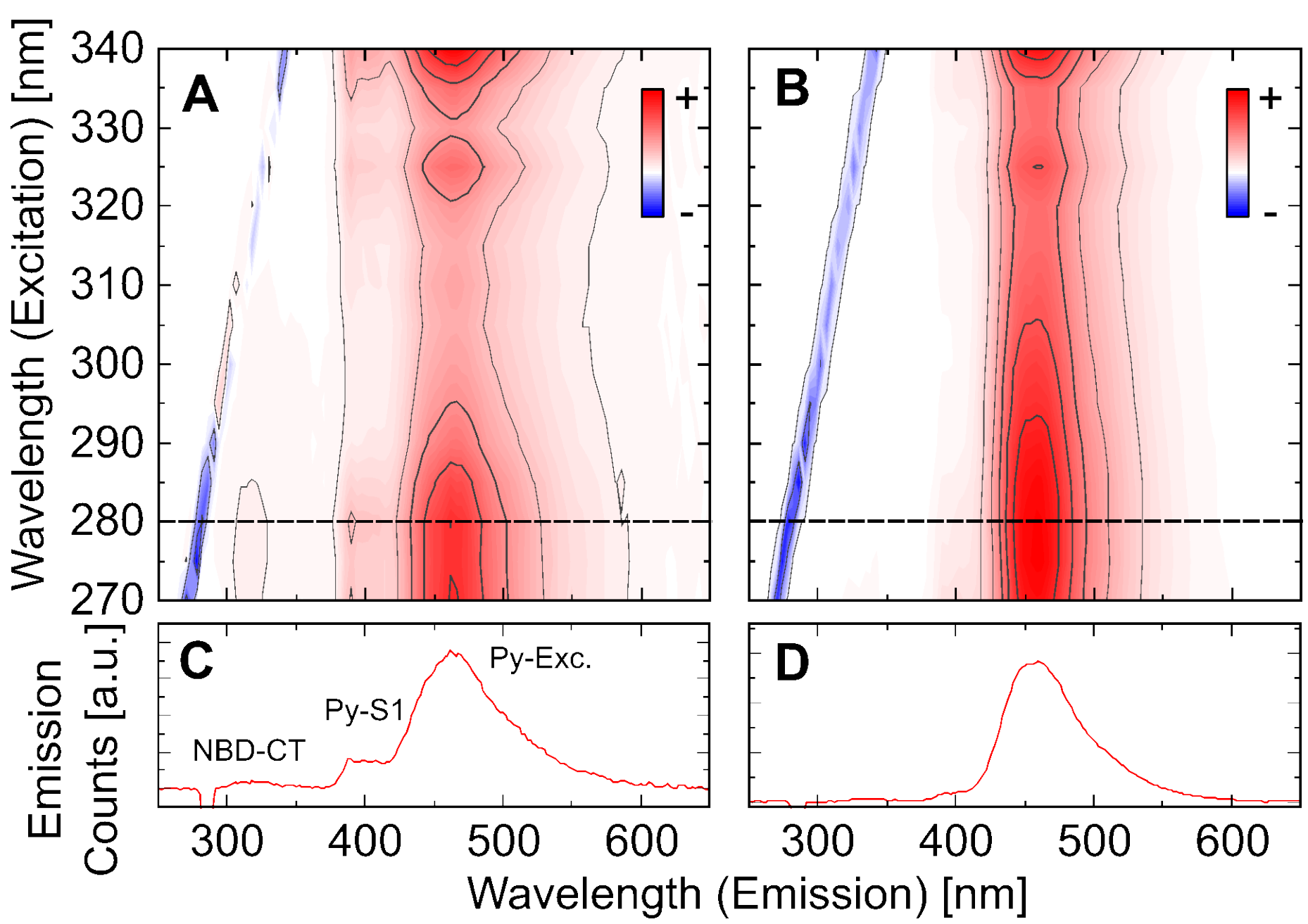

Figure S17: Emission profiles of 1NBDpy and 2NBDpy for different excitation wavelengths. A: 1NBDpy; B: 2NBDpy; C,D: Emssion spectra at the excitation wavelength of 280 nm

Concentration-dependent measurements were also conducted for 1NBDpy, as the stronger S1

emission from pyrene could serve as reference for the excimer behavior. The concentration started as $3.5 * 10^{-6} M$ and was diluted down to $1/16$ of the starting concentration (Figure S18). However, both the pyrene singlet emission and the excimer emission decrease proportionally, whilst the excimer emission persists even at very low concentrations. These results indicated that ground state dimers or undissolved clusters might be present in the solution. We have further diluted the sample to $1 * 10^{-9} M$ for steady state absorption measurements, but could not detect any significant shift of the absorption peaks. Further investigations are therefore required to test the dimer hypothesis.

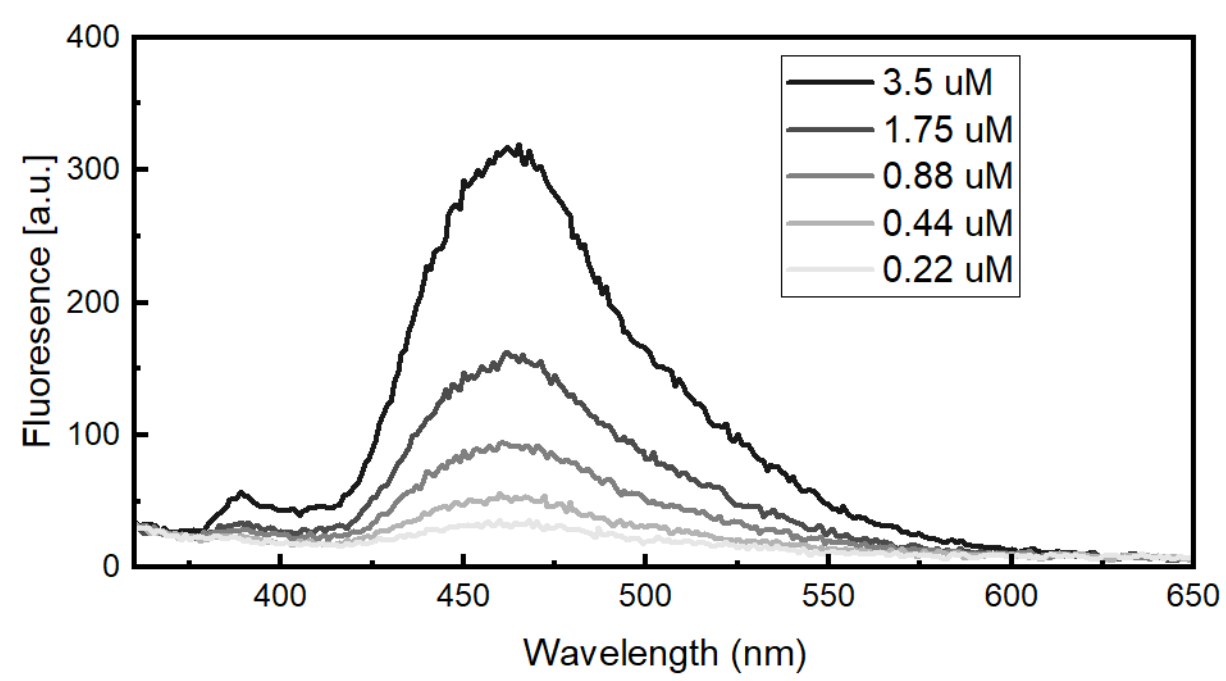

Figure S18: Fluorescence measurements of 1NBDpy at different concentrations. The emission from the starting concentration is presented as a solid black line whilst the diluted ones are presented with increased opacity.

Ultrafast Pump-probe Measurements

The Ultrafast transient absorption measurements were conducted on a home-built pump-probe system powered by Ti: sapphire laser offered by Spectra Physics (Spitfire Ace-100F-1K; Mai Tai SP-NSI; Empower 45, Spectra-Physics), which provides the fundamental wavelength (FW) centered at 800 nm with around 100 fs full-width-half-maximum (FWHM) and 1 kHz repetition rate. A fraction of the FW is used to both seed and pump a self-made nonlinear optical parametric amplification, which is later up-converted into near-UV region by sum-frequency-generation in a BBO crystal. 3 of the applied excitation pulses were generated in this method (267 nm, 308, 340 nm). All the pulses which are applied to excite the samples possess FWHMs of 3-6 nm, to ensure the selectiviaty of the orthogonal switching, while the durations of the pulses had the FWHM from 50 fs to 60 fs as a

trade-off. The probing pulses were generated from the same FW by focusing into a CaF2 crystal, which provide stable probing wavelengths from 315 nm to 650 nm. The deep ultraviolet (DUV) suppercontimuum for probing pulse was generated in the same matter but with an upconverted FW at 400 nm. The DUV pulses had a spectral range from 250 nm to 390 nm after filter the upconverted FW.

The samples were prepared in a 1 mm quartz cuvette in both acetonitrile. The concentrations were adjusted to aroud 0.7 OD for the peak of maximum absorption within the probing range (around $1.5 * 10^{-4} M$). The excitation laser pulses (267, 308 nm and 340 nm) had power in the range of 10-30 nJ. For the experiments for backisomerization from QC to NBD, the cuvette was exposed to 300 nm LED (M300L3, Thorlabs) to populte the QC form of the samples. The transmittence were captured with a customized spectrometer from Tec5. The femto- to 2 ns measurements were conducted with a optical delay from a piezo stage. The nano-to microsecond measurements were recorded with electronic controlled delay.

Nanosecond Transient Absorption Measurements of NBD-Ts

The early nanosecond dynamics is well matched to the radical ion pair difference spectrum in the main manuscript (Figure S19). The triplet state of NBD-Ts presents 2 features: i) braod absorption band extended up to 550 nm;[2] ii) ground state bleach below 345 nm. Both signals decays with a lifetime of 345 ns, which agrees well with literature.

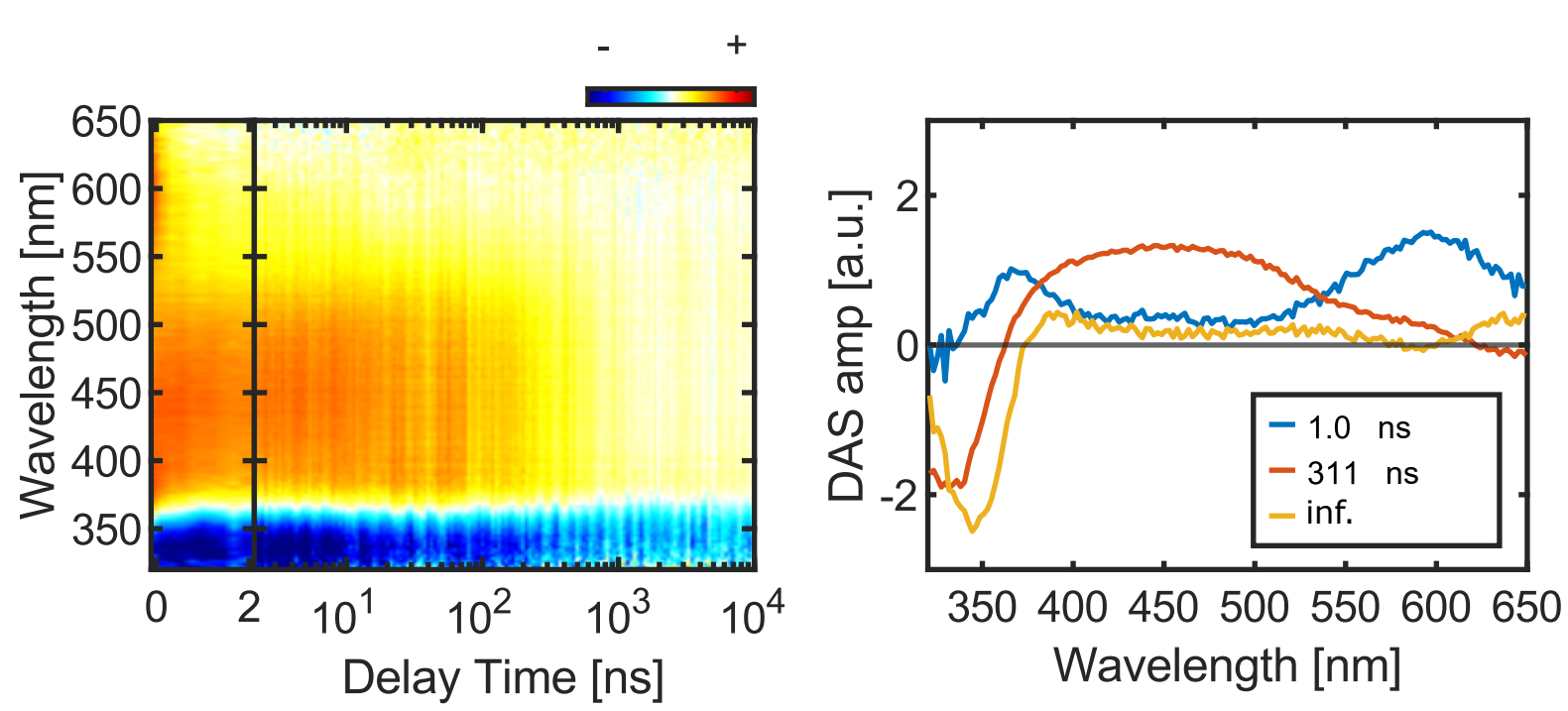

Figure S19: Transient absorption data (left) and the corresponding decay associated spectra (right) of NBD-Ts excited by 340 nm pulses in nanosecond range. The positive signals present the excited state absorption or product formation, negative signals present the ground state bleach.

Picosecond Transient Absorption Measurements of Pyrene

The excited state dynamics of pyrene shows a iconic S2 ESA at 575 nm on a 100 fs time scale. The later ESA from S1 absorption is fit to 2 lifetimes of 11.3 ps and over nanosecond (15.4 ns in manuscript).

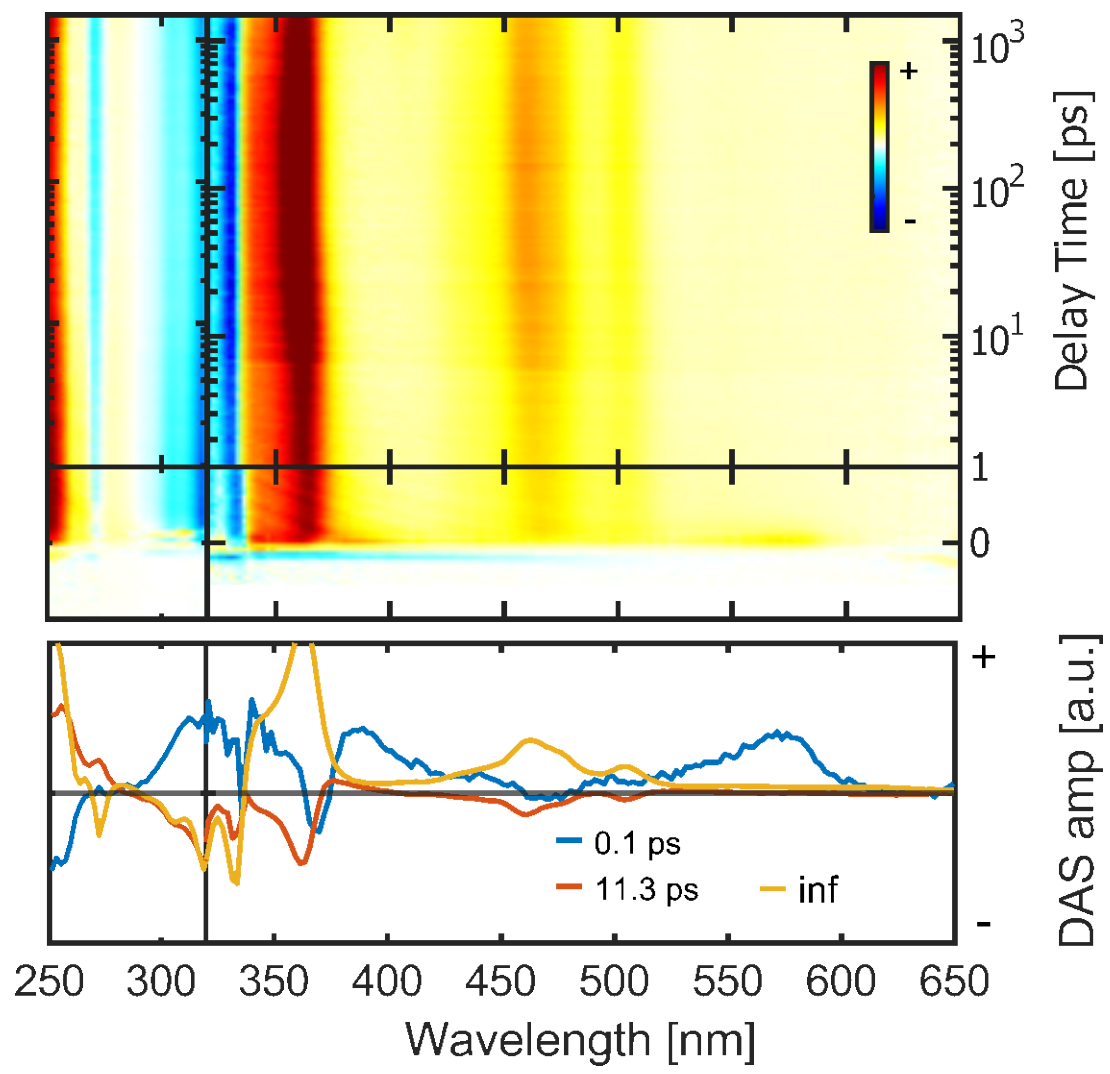

Figure S20: Transient absorption data (top) and the corresponding decay associated spectra (bottom) of pyrene excited by 340 nm pulses in picosecond range.

State-associated Spectra for Pyrene-linked NBDs in picosecond Scale

The excited state dynamics of 1NBDpy and 2NBDpy present complicated reaction sequences, so a complete model-based target anaysis would be difficult. However, a sequential model was applied to fit the transient data of all measurements on the picosecond scale for pyrene-linked NBDs to resolve the major components for the reactions. The analysis below served as a reliable reference and made it possible to easily identify the absorption spectra of species in the excited state.

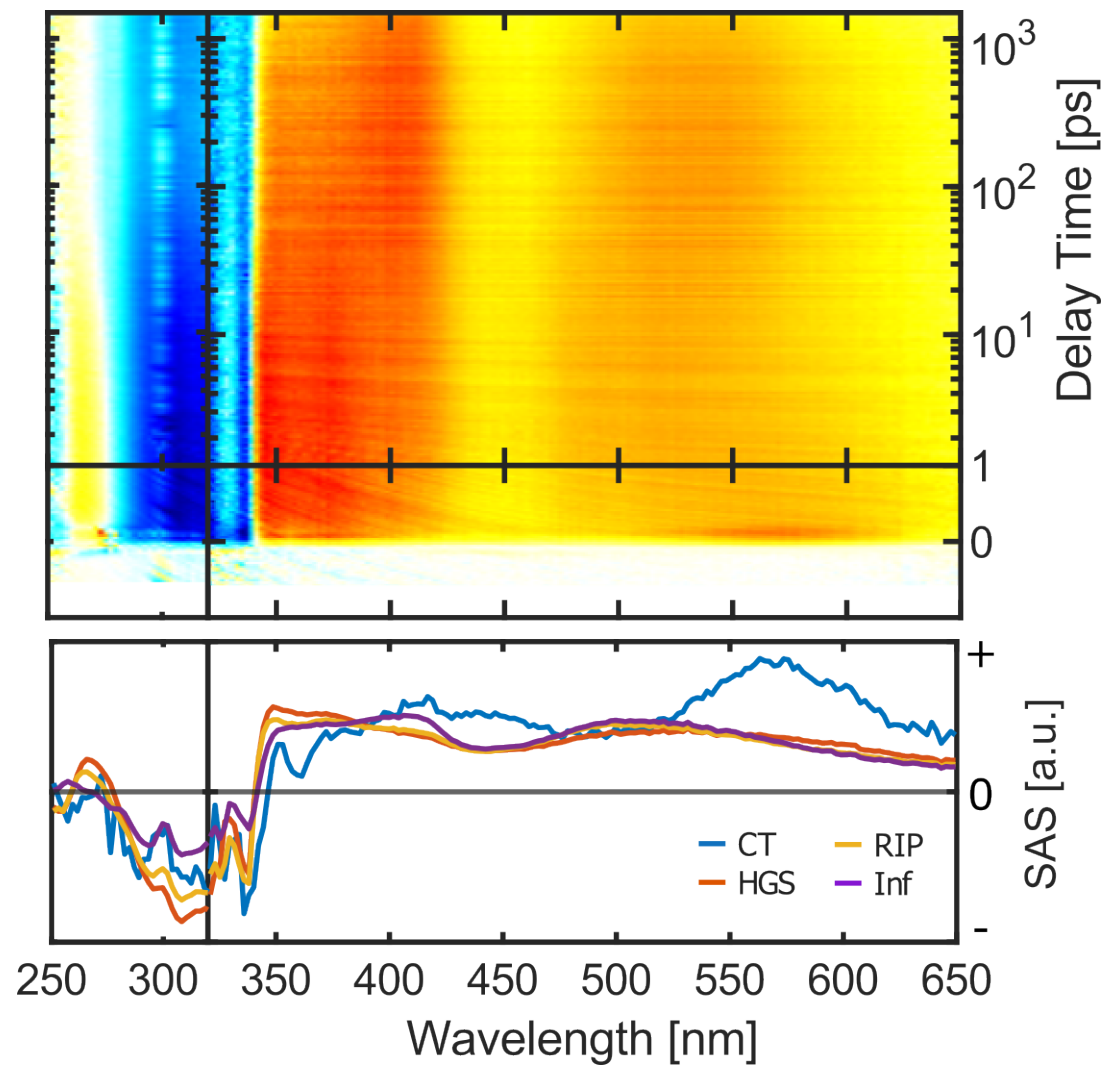

Figure S21: Transient absorption data (top) and the corresponding state-associated spectra (bottom) of 1NBDpy excited by 308 nm pulses in picosecond range.

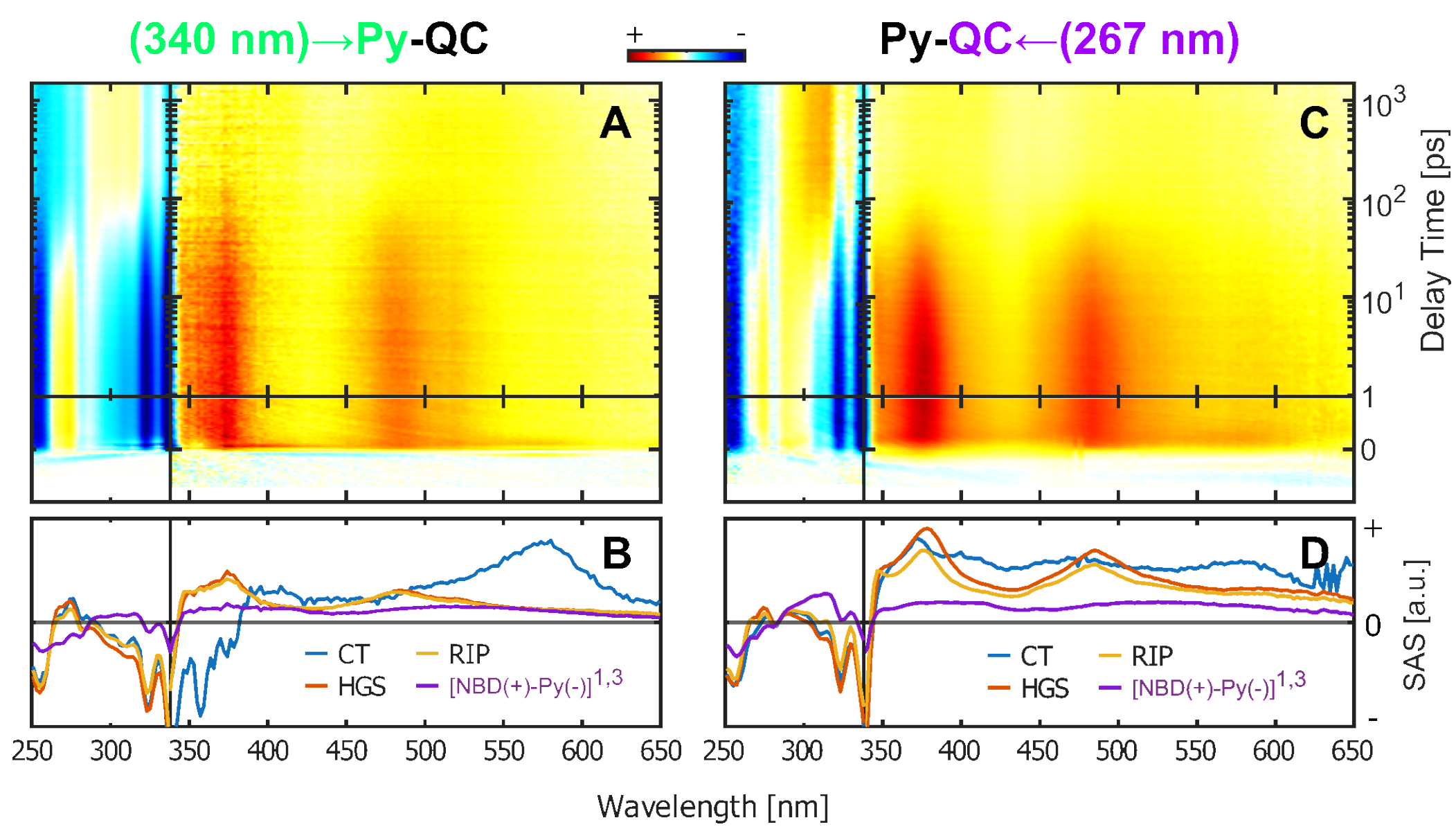

Figure S22: Transient absorption data (top) and the corresponding state-associated spectra (bottom) of 1QCpy excited by 340 nm and 267 pulses in picosecond range.

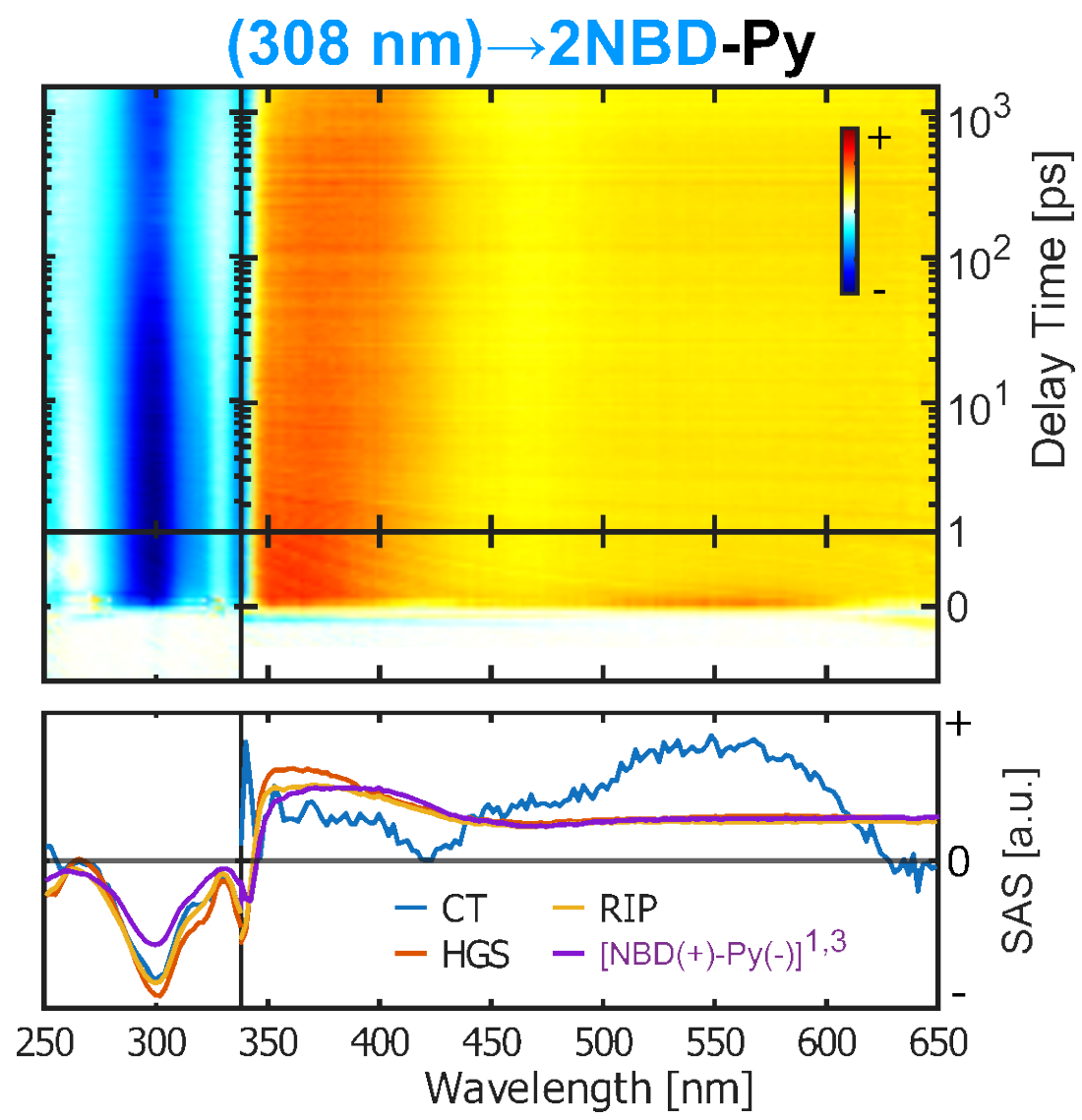

Figure S23: Transient absorption data (top) and the corresponding state-associated spectra (bottom) of 2NBDpy excited by 308 nm pulses in picosecond range.

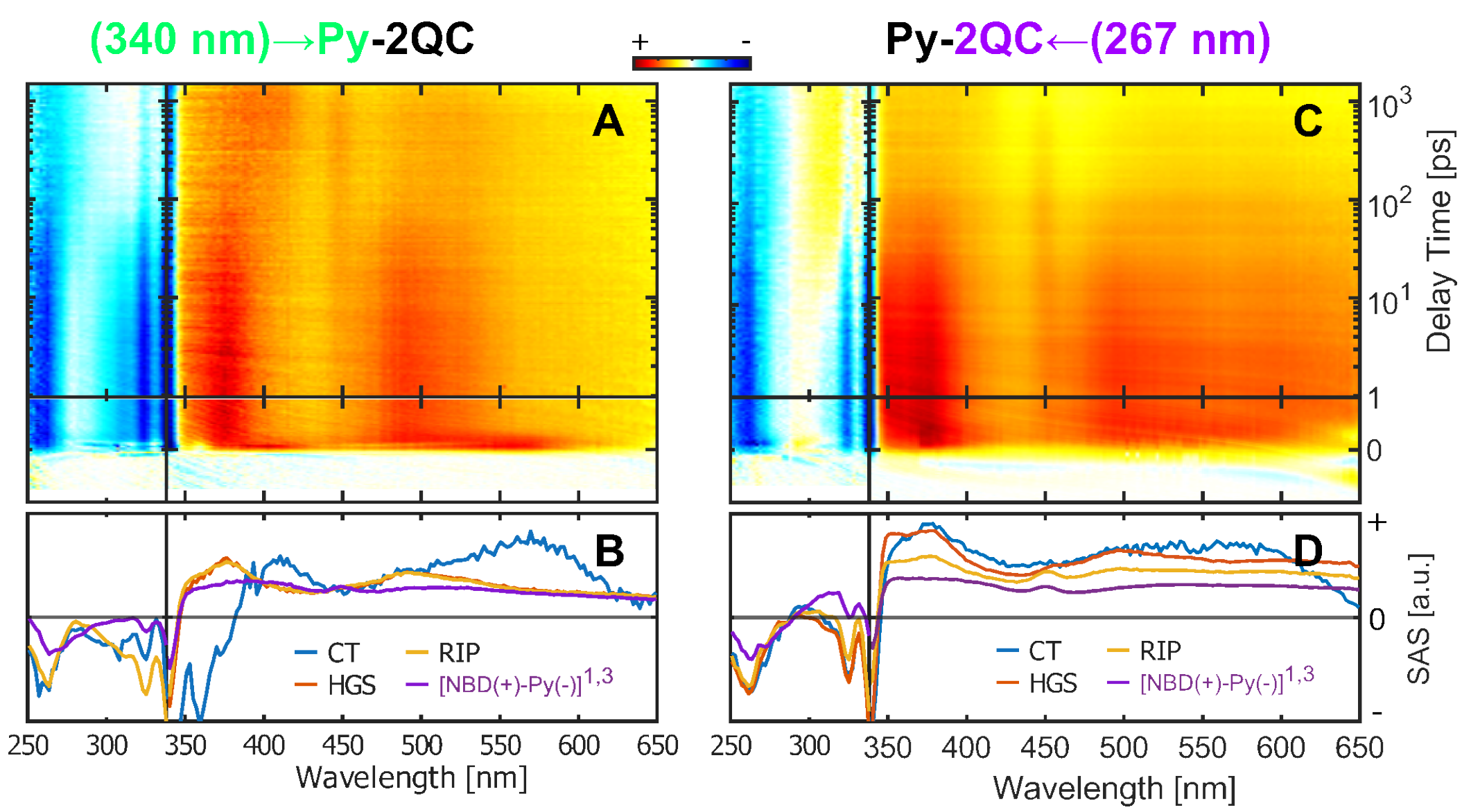

Figure S24: Transient absorption data (top) and the corresponding state-associated spectra (bottom) of 2QCpy excited by 340 nm and 267 pulses in picosecond range.

Table S5: Summary of transient absorption assignments and apparent rate constants. CT: charge transfer state; HGS: hot ground state; NBD^3: triplet of NBD; Py^1: excited singlet state of pyrene; $HRIP^1$: vibronic hot radical ion pair; $[NBD^+–Py^-]^3$: NBD-pyrene radical ion pair; Py^3: triplet state of pyrene; $QC^+–Py^-$: QC-pyrene radical ion pair.

Sample	Excited State	Absorption features	Lifetimes	Apparent k
NBD-Ts	CT	broad abs. in detection range	< 0.11 ps	k_1
	HGS	blue-shifting abs. at 270nm and 360 nm	~ 1.7 ps	k_2
	NBD^+	narrowing abs. at 605 nm, red-shifted 350 nm absorption	$\sim$ 60 ps (Hot), 343 ps	$k_3, k_{3'}$
	NBD^3	broad abs. from 360nm to 550 nm	311 ns	k_4
QC-Ts	CT	broad abs. in detection range	< 0.1 ps	k_5
	HGS	narrowing abs. at 350 nm	4.6 ps, 44.2	$k_6, k_{6'}$
1NBDpy	CT, Py^1	broad abs. in detection range, 575 nm	< 0.3 ps	k_7
	HGS, $HRIP^1$	blue-shifting abs. at 270nm and 350 nm, weak abs. at 275 nm 375 nm and broad abs. at 550 nm	8.5 ps, 73 ps	$k_8, k_{8'}$
	$[NBD^+–Py^-]^3$	410 nm and 525 nm	5-6 ns	k_9
	Py^3,NBD^3	390, 410, 480, and 515 nm, 350-550 nm	133 ns	k_{10}
1QCpy	CT, Py^1	broad abs. in detection range, 575 nm	< 0.3 ps	k_{11}
	HGS	red-shifting at 275 nm and blue-shifting at 350nm (Narrowing)	7-8 ps	k_{12}
	$QC^+–Py^-$	275, 375 and 485 nm	40-60 ps	k_{13}
	$[NBD^+–Py^-]^3$	410 nm and 525 nm	5-7 ns	k_{14}
	Py^3,NBD^3	390, 410, 480, and 515 nm, 350-550 nm	117-159 ns	k_{15}
2NBDpy	CT, Py^1	broad abs. in detection range, 575 nm	< 0.1 ps	k_7
	HGS, $HRIP^1$	blue-shifting abs. at 270nm and 350 nm, braod abs. from 470-650 nm	1.7 ps, 55,3 ps	k_8
	$[NBD^+–Py^-]^3$	broad abs. around 410 nm and braod abs. from 470-650 nm	6-8 ns	k_9
	Py^3,NBD^3	390, 410, 480, and 515 nm, 350-550 nm	116 ns	k_{10}
2QCpy	CT, Py^1	broad abs. in detection range, 575 nm	< 0.2 ps	k_{11}
	HGS	weak red-shifting at 275 nm and blue-shifting at 350nm (Narrowing)	1-7 ps	k_{12}
	$QC^+–Py^-$	275, 375 and 485 nm	41-103 ps	k_{13}
	$[NBD^+–Py^-]^3$	broad abs. around 410 nm and braod abs. from 470-650 nm	5-7 ns	k_{14}
	Py^3,NBD^3	390, 410, 480, and 515 nm, 350-550 nm	152 ns	k_{15}

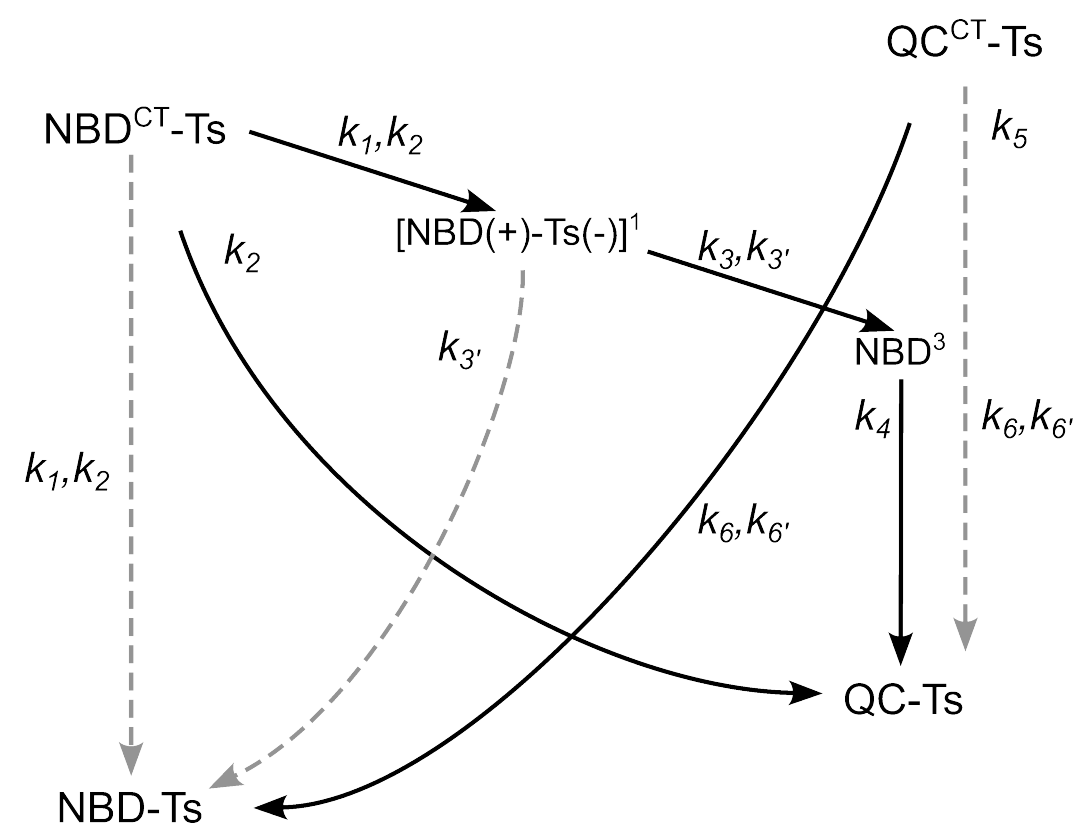

Figure S25: Relaxation pathways of NBD-Ts and QC-Ts with the apparent k values in Table S5. The solid arrows indicate the productive pathways while the dashed arrows indicate the nonproductive pathways.

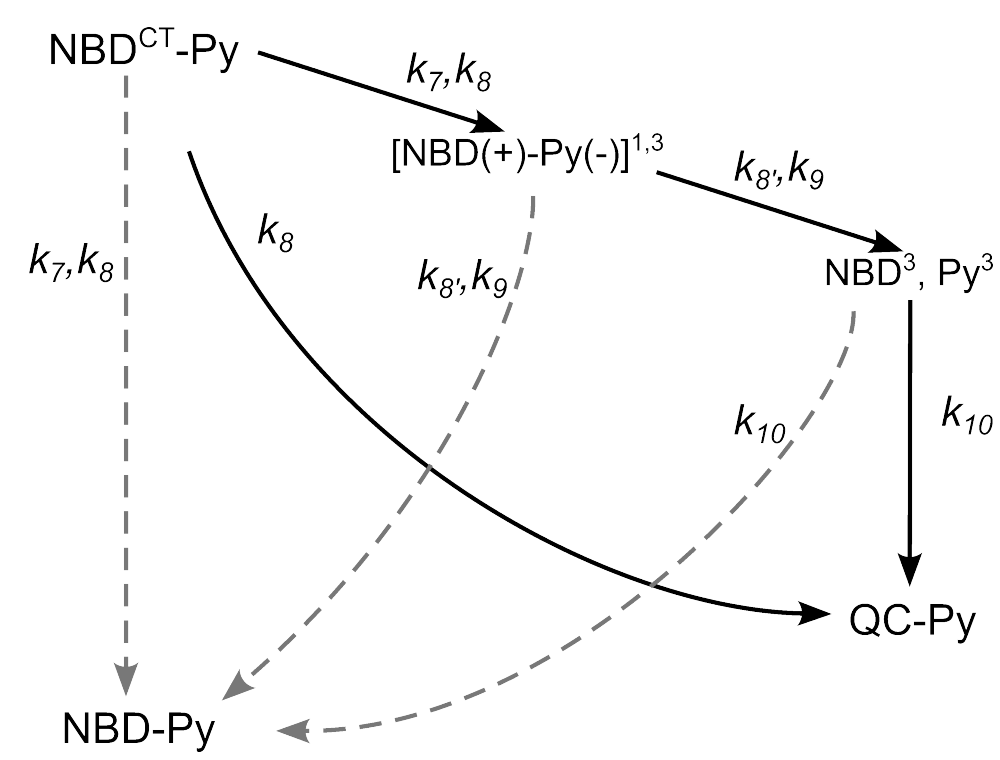

Figure S26: Relaxation pathways of excited pyrene-linked NBDs (1NBDpy and 2NBDpy) with the apparent k values in Table S5. The solid arrows indicate the productive pathways while the dashed arrows indicate the nonproductive pathways.

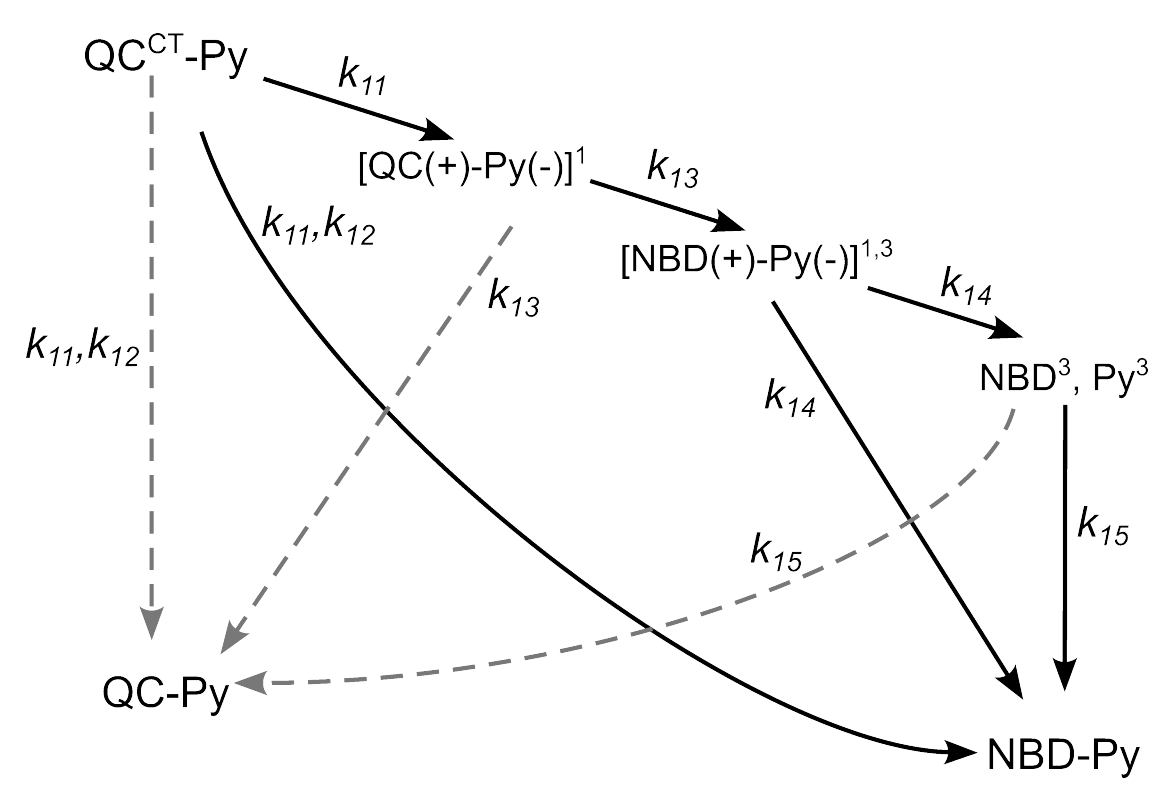

Figure S27: Relaxation pathways of excited pyrene-linked QCs (1QCpy and 2QCpy) with the apparent k values in Table S5. The solid arrows indicate the productive pathways while the dashed arrows indicate the nonproductive pathways.

Quantum Chemical Calculations

Geometry optimizations for all molecules were performed with Gaussian 16 employing the CAM-B3LYP density functional together with the def2-SVP basis set. The optimized structures were confirmed to be stationary points by vibrational analysis without any imaginary modes. Excited state energies as well as attachment/detachment densities were calculated with QChem 5.1 using the same functional and basis set combination.

Aside from the energy levels from each excited states, the charge transfer features could be observed in the attachment/detachment maps. As for pyrene-linked NBDs, the delocalization of the excitation is indicated by the extension of the attachment map to the NBD cores (Figure S28,S30, right lower corner). And for pyrene-linked QC, the pyren to tolylsulfonyl charge transfer features are presented in higher energy levels (Figure S29,S31). Both are in good agreement with the steady state absorption spectra and the proposed reaction mechanism.

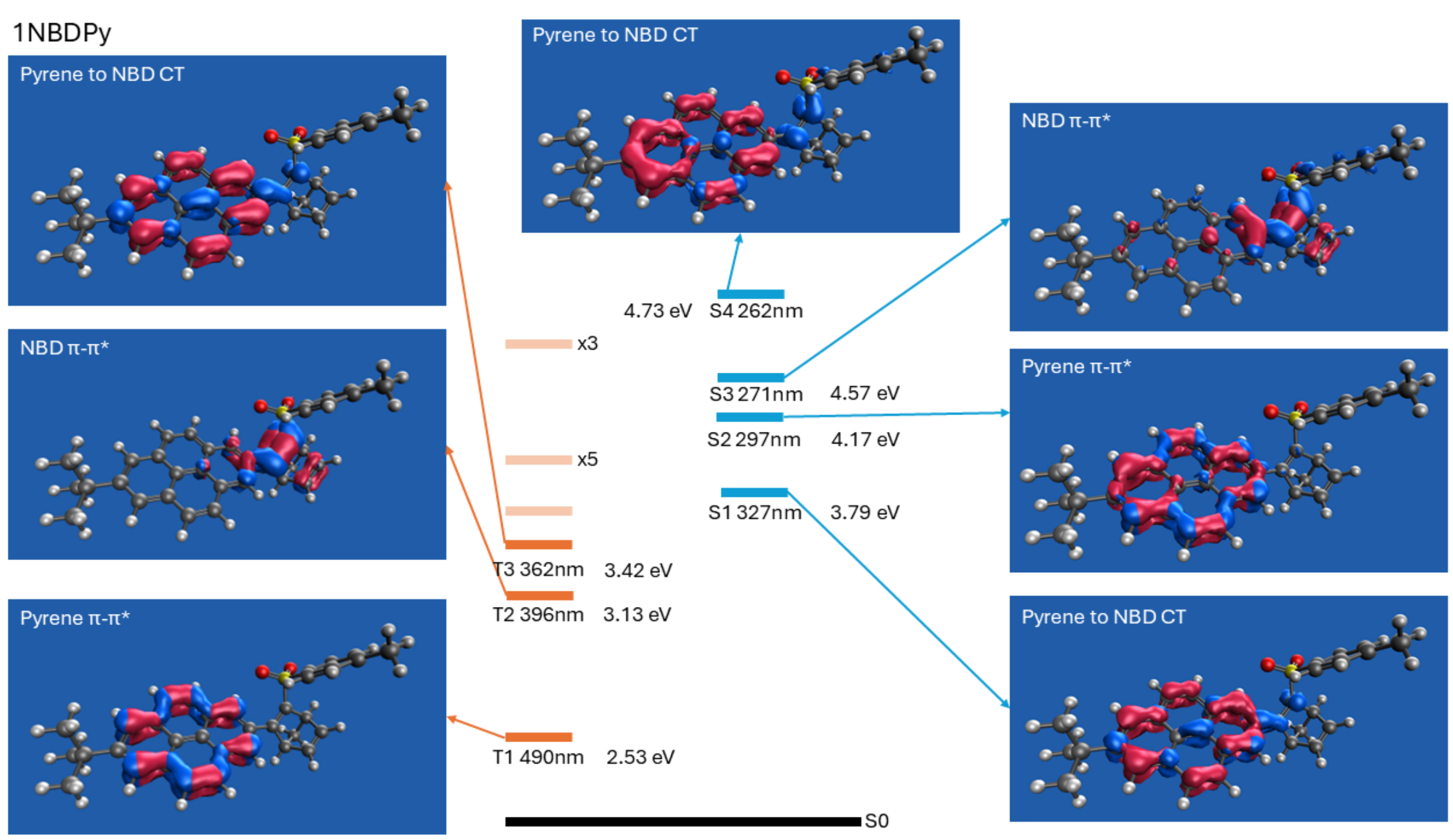

Figure S28: Detachment (red) and attachment (blue) densities of the excited states of 1NBDpy for triplet (left) and singlet (right) state.

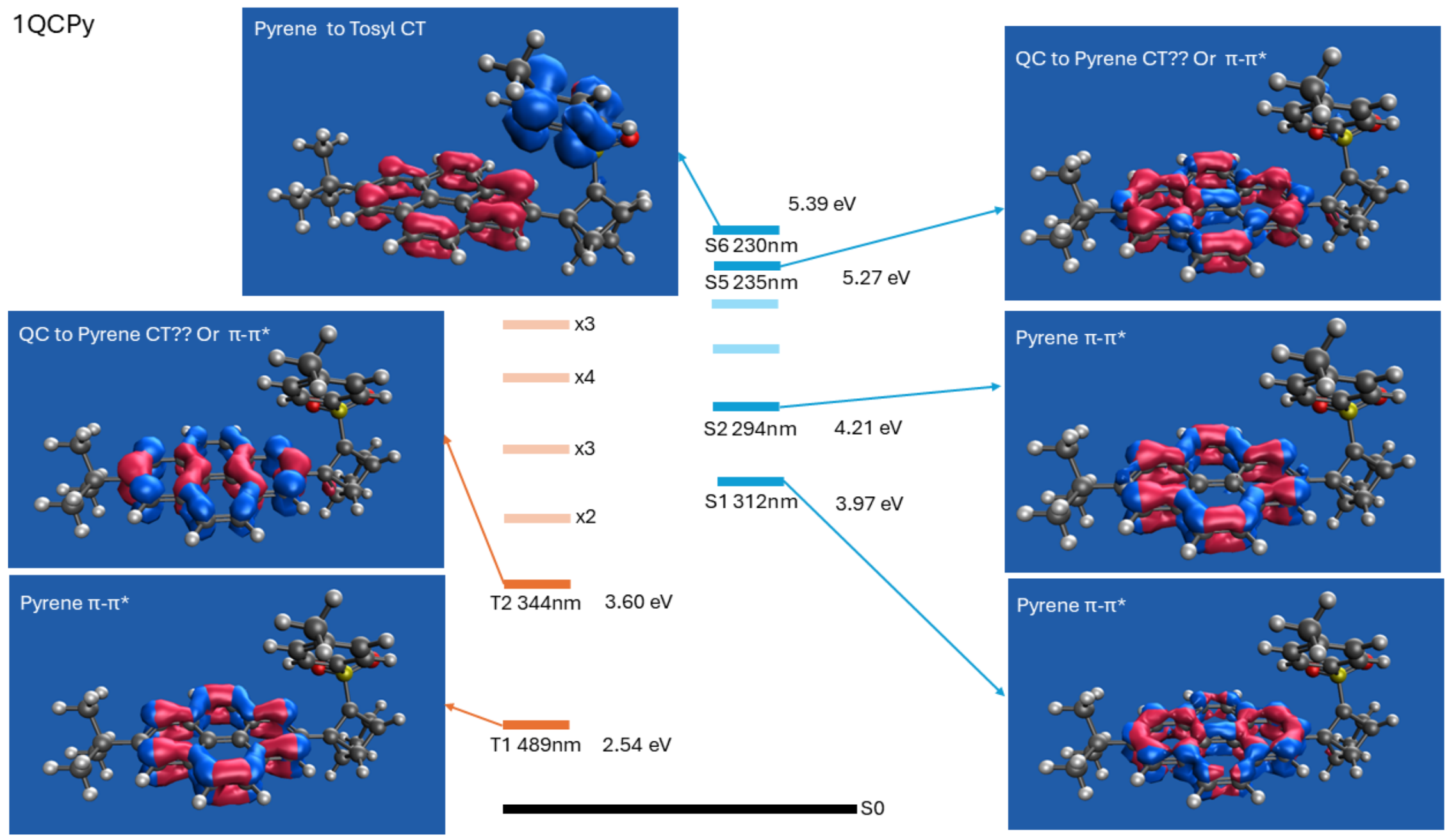

Figure S29: Detachment (red) and attachment (blue) densities of the excited states of 1QCpy for triplet (left) and singlet (right) state.

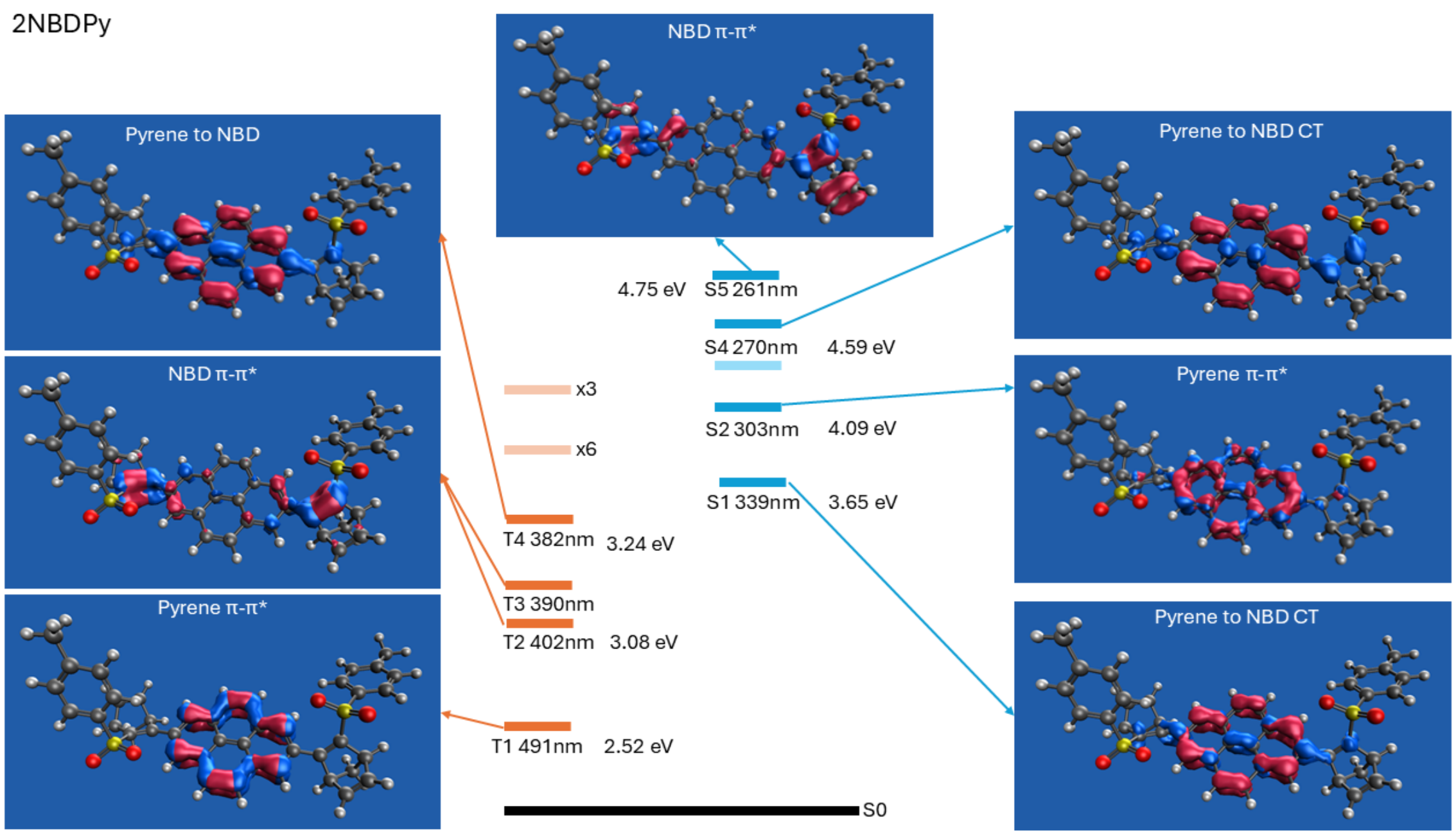

Figure S30: Detachment (red) and attachment (blue) densities of the excited states of 2NBDpy for triplet (left) and singlet (right) state.

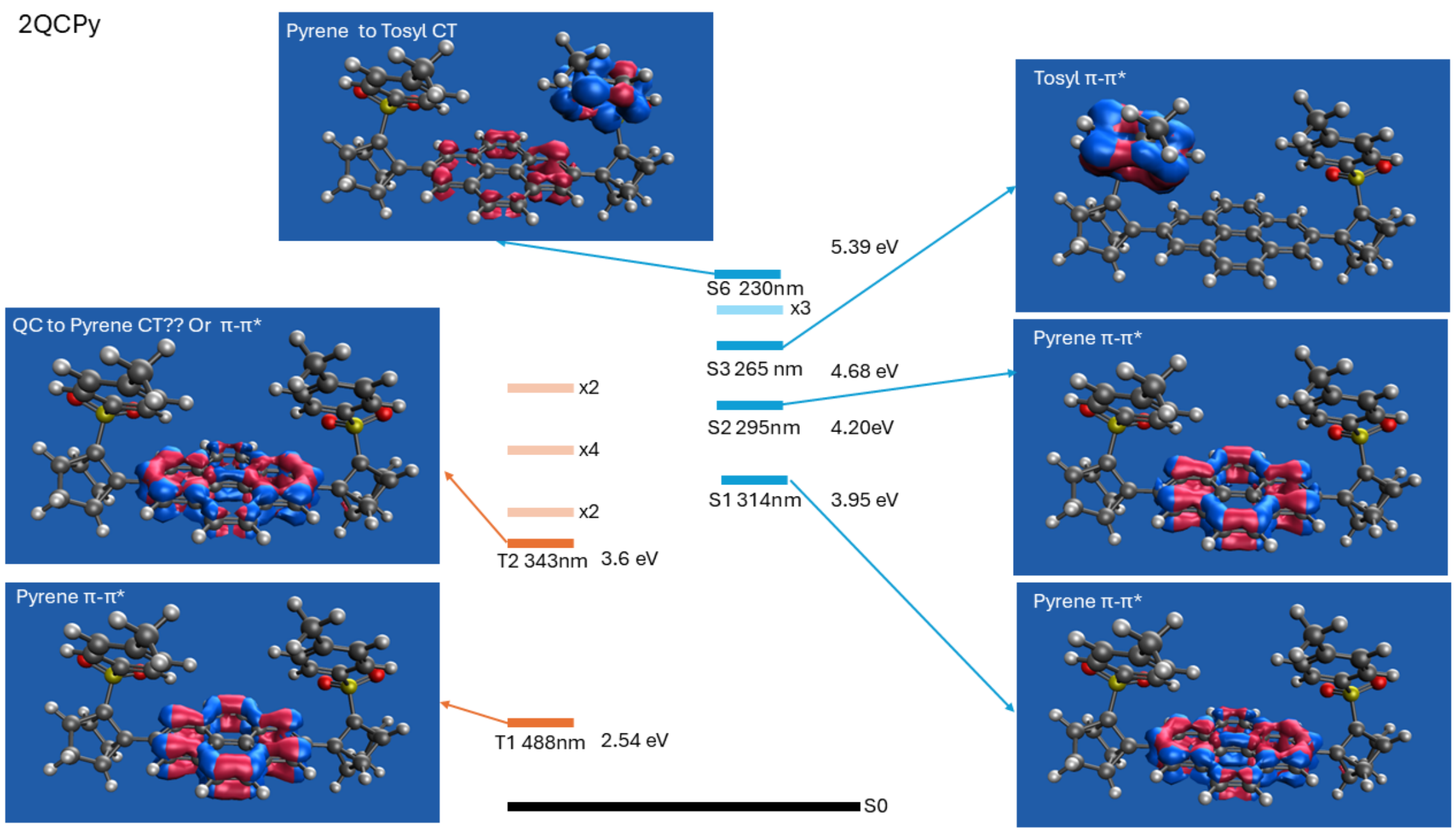

Figure S31: Detachment (red) and attachment (blue) densities of the excited states of 2QCpy for triplet (left) and singlet (right) state.

References

(1) Stranius, K.; Börjesson, K. Determining the Photoisomerization Quantum Yield of Photoswitchable Molecules in Solution and in the Solid State. *Sci Rep* **2017**, *7*, 41145.

(2) Alex, W.; Lorenz, P.; Henkel, C.; Clark, T.; Hirsch, A.; Guldi, D. M. Solar Energy Storage: Competition between Delocalized Charge Transfer and Localized Excited States in the Norbornadiene to Quadricyclane Photoisomerization. *J. Am. Chem. Soc.* **2022**, *144*, 153–162.
